# LncRNA 1700020I14Rik promotes AKR1B10 expression and activates Erk pathway to induce hepatocyte damage in alcoholic hepatitis

**DOI:** 10.1038/s41420-022-01135-w

**Published:** 2022-08-26

**Authors:** Yue Wu, Yabin Qi, Yangqiu Bai, Haihui Zhang, Wenliang Zhu, Shengli Zhou, Yanrui Zhang

**Affiliations:** 1 Department of Gastroenterology, Henan Provincial People’s Hospital, Zhengzhou University People’s Hospital, Zhengzhou, Henan China; 2 Department of Critical Care Medicine, Henan Provincial People’s Hospital, Zhengzhou University People’s Hospital, Zhengzhou, Henan China; 3 Department of Pathology, Henan Provincial People’s Hospital, Zhengzhou University People’s Hospital, Zhengzhou, Henan China

**Keywords:** Alcoholic liver disease, Cell biology

## Abstract

Alcoholic hepatitis (AH), a kind of alcoholic liver disease, shows poor prognosis. Long noncoding RNAs (lncRNAs) exert critical role in liver diseases. Here, we intended to investigate the possible molecular mechanism that 1700020I14Rik-based regulation of microRNA (miR)-137/Aldo-keto reductase family 1 member B10 (AKR1B10) affecting the inflammatory response and hepatocyte damage in AH. AH-related genes and the down-stream regulatory pathway were screnned by bioinformatics. Mouse normal hepatocyte cell line AML12 was selected to construct an ethanol-induced hepatocyte injury model for in vitro mechanistic validation, while we also established an AH mouse model using the ethanol with gradually increased concentration of 2–4% (v/v) for in vivo study. Specific role of 1700020I14Rik/miR-137/AKR1B10 in AML12 cell viability, proliferation and apoptotic capacity as well as inflammation and liver damage in mice were analyzed following ectopic and depletion approaches. We found elevated AKR1B10 and 1700020I14Rik but reduced miR-137 in AH. 1700020I14Rik was able to elevated miR-137-mediated AKR1B10. In vitro cell experiments and in vivo animal experiments validated that 1700020I14Rik reduced ethanol-induced hepatocyte damage and inflammation in AH mice through regulation of miR-137–mediated AKR1B10/Erk axis. The current study underlied that 1700020I14Rik could activate AKR1B10/Erk signaling through inhibition of miR-137, thereby promoting the hepatocyte damage in AH mice.

## Introduction

Alcoholic hepatitis (AH), the severest clinical presentation of alcoholic liver disease, is featured by liver failure under recent and heavy alcohol intake [1]. Importantly, the pathogenesis of alcoholic-related liver disease shares correlation with multiple factors, including alcohol-induced hepatocyte damage, steatosis caused by gut-derived microbial components, recruitment and activation of inflammatory cells in liver, reactive oxygen species as well as genetic factors, which increases the difficulty of treating AH [2]. It has been documented that AH sufferers show poor prognosis and the recovery of AH largely depends on abstinence due to the shortage of an effective pharmacologic treatment [3]. Of note, activation of inflammasome has been identified as a key contributor to hepatocyte damage and deterioration of liver inflammation [4]. Therefore, to get an in-depth understanding of the inflammatory reaction and hepatocyte damage is of critical to be clearer about the target of treating AH.

Long noncoding RNAs (lncRNAs) are well-characerized gene mediators engaging in different functional activities by diverse mechanisms [5]. Significance of lncRNAs in human liver disease has been highly documented [6]. Notably, the promising candidates for lncRNAs as biomarkers in modulating inflammation have been well-studied [7]. It has been reported that overexpression or knockdown of 1700020I14Rik (ENSMUST00000147425) was able to affect cell proliferation and fibrosis in mouse mesangial cells [8]. However, the role of 1700020I14Rik in AH is hardly known, which warrants further exploration. Moreover, the lncRNAs-microRNANs (miRNAs) interaction shows great potential for developing new therapeutic way and biomarkers in liver disease [9]. Our bioinformatics prediction results showed that 1700020I14Rik may regulate microRNA-137 (miR-137) expression. It has been shown that miR-137 is poorly expressed in hepatocellular carcinoma (HCC), and is capable of repressing HCC cell malignant properties [10, 11]. We further predicted that miR-137 was correlated with the expression of Aldo-keto reductase family 1 member B10 (AKR1B10). AKR1B10 possesses potentials in cancer development and progression, which has emerged as a diagnostic target for tumors [12]. Furthermore, elevated AKR1B10 is detected in steatohepatitis [13], yet the effect of AKR1B10 on AH deserved explored in-depth. Additionally, AKR1B10 can activate the Erk signaling pathway to affect cancer development [14, 15]. Activation of the Erk signaling pathway exerts carcinogenic role in HCC, accompanied with poor prognosis [16]. Considering the above evidence, the present study tried to elucidate the specific role of 1700020I14Rik-miR-137-AKR1B10 axis in AH.

## Results

### AKR1B10 is significantly highly expressed in the liver tissues of AH mice

**Fig. 1:**
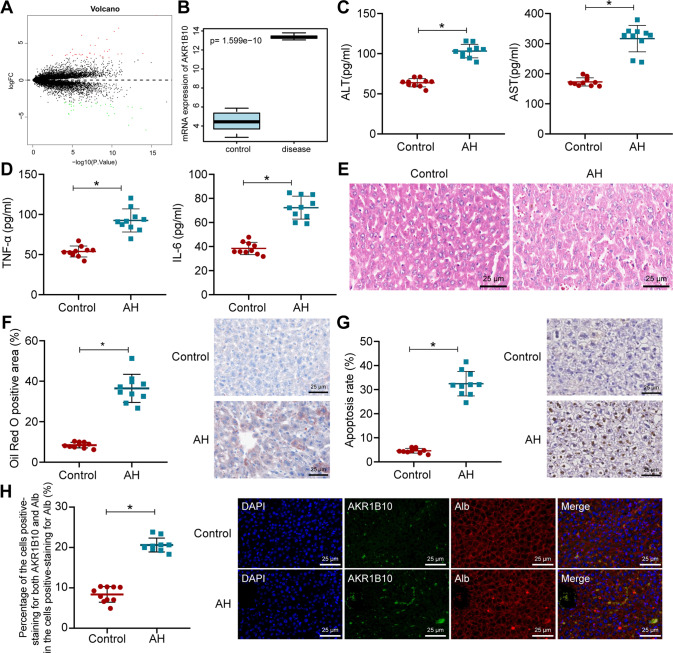
AKR1B10 expression is highly expressed in the liver tissues of AH mice. **A** Volcano map of the gene expression obtained from the GSE28619. The│logFC│ > 0 indicates an upregulated gene; │logFC│ < 0 indicates downregulated gene. Red dots represent the significantly upregulated genes; green points represent the significantly downregulated genes. **B**, Boxplot of AKR1B10 mRNA expression in microarray GSE28619. The left blue box shows the expression of the normal samples (*n* = 7); the right red box shows the expression of AH patient samples (*n* = 15). **C**, **D** Serum levels of ALT, AST, TNF-α, and IL-6 as ELISA detected. **E** Liver damage in liver tissues of AH mice as H&E staining measured (400×). **F** Lipid drops in liver tissues of AH mice by oil red O staining (400×). **G** Number of apoptotic cells in AH mice determined by TUNEL staining (400×). **H** AKR1B10 expression in hepatocytes of liver tissues from AH mice by IF (400×). For panel **C**–**H**, ten mice in each treatment. *p < 0.05 compared to the control group. Data are shown as the mean ± standard deviation of three technical replicates. Data between two groups were compared by unpaired *t*-test.

Analysis of AH-related microarray GSE28619 yielded 67 DEGs, including 34 upregulated genes and 33 downregulated genes (Fig. 1A), and AKR1B10 showed the largest │logFC│. Through boxplots, we found significantly high expression of AKR1B10 in liver tissues of AH patients (Fig. 1B).

For validation of the established AH mouse model, enzyme-linked immunoassay (ELISA) was carried out with results showing that serum levels of alanine transaminase (ALT), aspartate aminotransferase (AST), TNF-α, and IL-6 were increased in the AH mice (Fig. 1C, D). Additionally, AH mice showed severe liver damage, significant inflammatory cell infiltration, and increased lipid droplets as well as induced apoptosis of mouse hepatocytes (Fig. 1E–G). The above results validated the successful establishment of AH model. Further immunofluorescence (IF) also identified a significant increase in AKR1B10 expression in hepatocytes of live tissues from AH mice (Fig. 1H).

The above results depicts that AKR1B10 is elevated in the liver tissues of AH mice.

### Silencing of AKR1B10 attenuates ethanol-induced hepatocyte damage

**Fig. 2:**
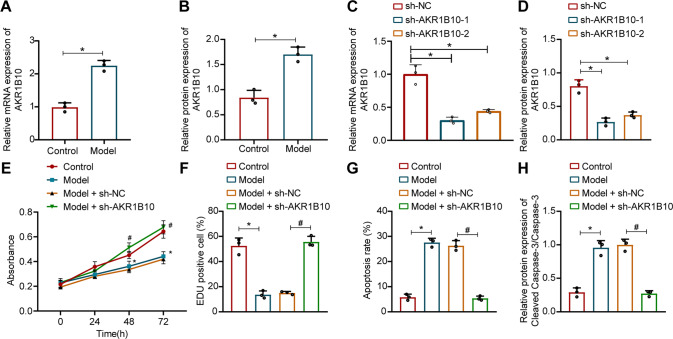
Downregulation of AKR1B10 reduces ethanol-induced damage to AML12 cells. **A**, **B** AKR1B10 expression in ethanol-treated AML12 cells measured by RT-qPCR and Western blot assay (*p < 0.05 compared with the control). **C**, **D** AKR1B10 silencing efficiency in ethanol-treated AML12 cells transduced with sh-AKR1B10-1/2 measured by RT-qPCR and Western blot assay (*p < 0.05 com*p*ared to the sh-NC group). **E** Ethanol-treated AML12 cell viability upon sh-AKR1B10 treatment determined by the CCK-8 experiments. **F** Ethanol-treated AML12 cell proliferation upon sh-AKR1B10 treatment determined by EdU staining. **G** Apoptosis rate of ethanol-treated AML12 cells upon sh-AKR1B10 treatment assayed by flow cytometry. **H** Protein expression of Cleaved Caspase-3 and Caspase-3 in ethanol-treated AML12 cells upon sh-AKR1B10 treatment analyzed by Western blot assay. For panel **E**–**H**, *p < 0.05 compared with the control group; #*p* < 0.05 compared with the Model + sh-NC group. Data are shown as the mean ± standard deviation of three technical replicates. The unpaired *t*-test was conducted for two group comparison, and one-way ANOVA and Tukey’s post-hoc tests for muti-group comparison. Comparison among groups at different time points was completed using two-way ANOVA followed by Bonferroni’s multiple comparison test.

We further validated AKR1B10 function at the cellular level. AML12 cells were treated with 50 mM ethanol for 24 h to construct ethanol-induced cell damage model. As reflected by RT-qPCR and Western blot assay, a significant increase of AKR1B10 expression was detected in the ethanol-induced AML12 cells (Fig. 2A, B, Supplementary Fig. 1A). Following construction of two AKR1B10 silencing seuqnece, we found that AKR1B10 expression was diminished in cells transfected with sh-AKR1B10-1 or sh-AKR1B10-2 (Fig. 2C, D, Supplementary Fig. 1B), and sh-AKR1B10-1 showed higher silencing efficiency and were used for subsequent experimentations.

Functional assays desmontrated that the cell viability and proliferation were limited but the apoptosis rate was increased under ethanol treatment in AML12 cells; however, opposing tedndecy was witnessed upon silencing of AKR1B10 (Fig. 2E–G, Supplementary Fig. 1C, D). Also, in ethanol-induced AML12 cells, elevated cleaved Caspase-3 expression was detected, but downregulation of AKR1B10 caused a reduction in cleaved Caspase-3 expression; while no significant difference was seen in Caspase-3 expression in each treatment (Fig. 2H, Supplementary Fig. 1E).

Thus, AKR1B10 is highly expressed in ethanol-induced hepatocytes, and silencing of AKR1B10 attenuates ethanol-induced hepatocyte damage.

### Silencing of AKR1B10 relieves liver injury in AH mice

**Fig. 3:**
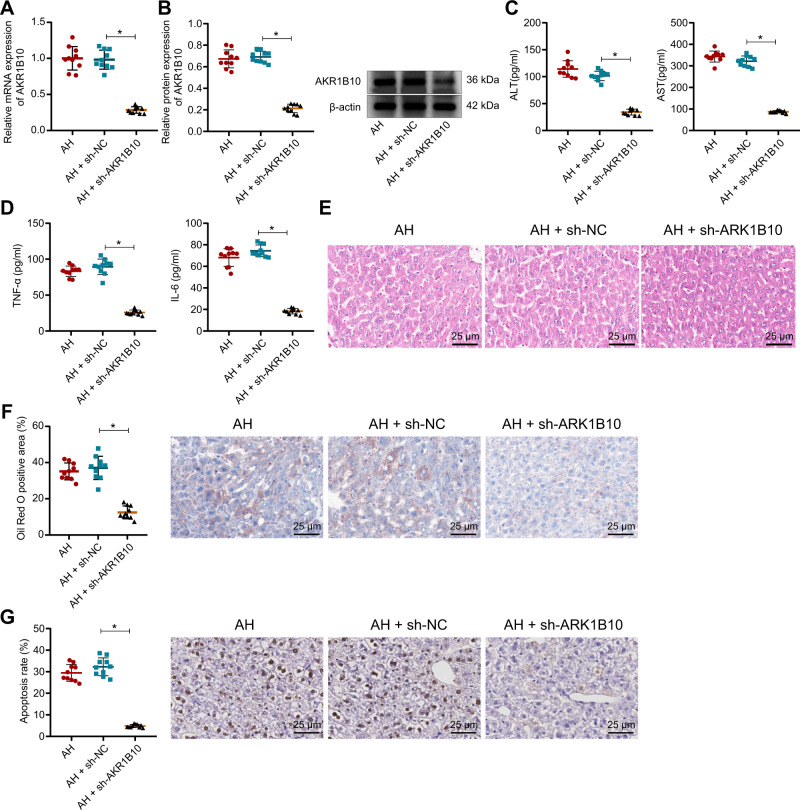
Downregulation of AKR1B10 reduces liver injury in AH mice. **A**, **B** RT-qPCR and Western blot assay detected the AKR1B10 expression in AH mice upon sh-AKR1B10 treatment. **C**, **D** ELISA for detection of serum ALT, AST, TNF-α, and IL-6 levels in AH mice upon sh-AKR1B10 treatment. **E** Liver damage in AH mice upon sh-AKR1B10 treatment as H&E staining measured (400×). **F** Lipid drops in liver tissues of AH mice upon sh-AKR1B10 treatment by oil red O staining (400×). **G** Number of apoptotic cells in AH mice upon sh-AKR1B10 treatment determined by TUNEL staining (400×). Ten mice in each treatment. *p < 0.05 compared to the AH + sh-NC group. Data are shown as the mean ± standard deviation of three technical replicates. Data between two groups were compared by unpaired *t*-test.

In vivo effect of AKR1B10 was our next focus. It was evident that AKR1B10 expression was distinctly reduced in liver tissues of AH mice injected with sh-AKR1B10 (Fig. 3A, B). Furthermore, the serum ALT, AST, TNF-α, and IL-6 levels were also decreased in the serum of AH mice injected with sh-AKR1B10 (Fig. 3C, D).

Pathological observation revealed that silencing of AKR1B10 further reduced liver damage, infiltration of inflammatory cells, and lipid droplets as well as the number of apoptotic cells in AH mice (Fig. 3E–G).

The above findings note that silencing of AKR1B10 attenuates liver injury in AH mice.

### miR-137 targets and inhibits AKR1B10 expression

**Fig. 4:**
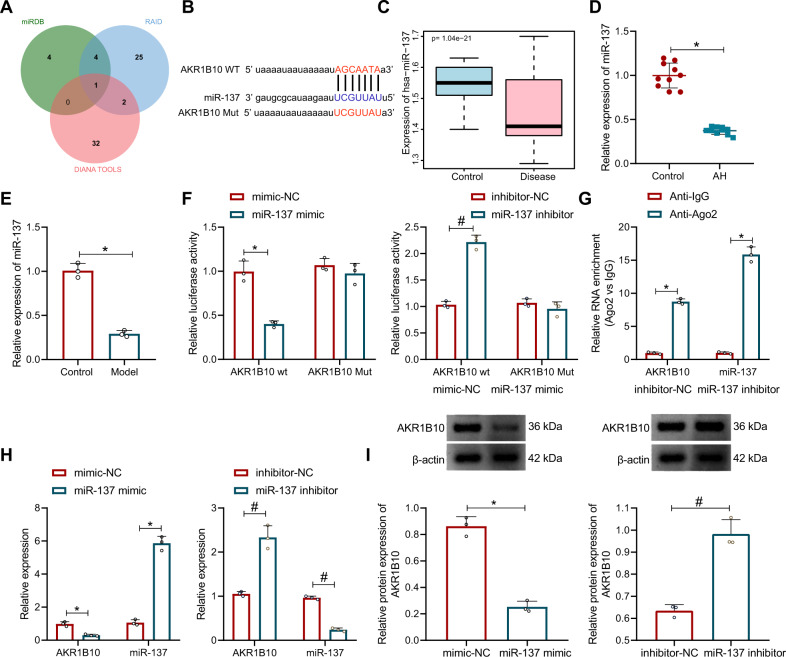
miR-137 targets AKR1B10. **A** Venn plot of the upstream miRNA of AKR1B10 obtained from the online prediction website miRDB, DIANA TOOLS, and RAID. **B** Plot of the binding site of AKR1B10 to miR-137 in RAID. **C** Box plot of miR-137 expression in GSE59492. The left blue box shows the expression of the normal samples (*n* = 6); the right red box shows the expression of AH patient samples (*n* = 13). **D** miR-137 levels in AH mice (*n* = 10) analyzed by RT-qPCR. *p < 0.05 compared with the control group. **E** miR-137 expression in the ethanol-induced AML12 cells measured by RT-qPCR. *p < 0.05 compared to the control group. **F** Dual-luciferase reporter assay detected the relationship of miR-137 and AKR1B10. *p < 0.05 compared with mimic-NC group; *p < 0.05 compared with inhibitor-NC group. **G** Interaction between AKR1B10 and miR-137 detected by RIP experiments. *p < 0.05 compared to the Anti-lgG group. **H** Protein expression of miR-137 and AKR1B10 in cells upon miR-137 mimic treatment determined by RT-qPCR. *p < 0.05 compared to the mimic-NC group. **I** AKR1B10 expression in cells upon miR-137 mimic or miR-137 inhibitor treatment measured by Western blot assay. *p < 0.05 compared to the mimic-NC group; *p < 0.05 compared with inhibitor-NC group. Data are shown as the mean ± standard deviation of three technical replicates. Data between two groups were compared by unpaired *t*-test.

To further investigate the regulatory mechanism upstream of AKR1B10, the upstream miRNA of AKR1B10 were predicted by the online prediction websites, obtaining 9, 35, and 32 miRNAs, respectively. Through Venn map, we obtained only one miRNA (miR-137) in the intersection of the three databases (Fig. 4A), and miR-137 had binding site with AKR1B10 (Fig. 4B).

Anlysis of the microarray data GSE59492 from the gene expression omnibus (GEO) database indicated that miR-137 was significantly poorly expressed in AH (Fig. 4C). Further expression determination in our work also confirmed a reduction in miR-137 expression in liver tissues of AH mice and ethanol-induced AML12 cells (Fig. 4D, E).

For further validation in HEK 293 T cells, luciferase activity assay clarified that the luciferase activity of co-transduction of miR-137 mimic with AKR1B10 wild type (Wt) was reduced, but no significant difference in the AKR1B10 mutant type (Mut) group; meanwhile, the luciferase activity of co-transduction of miR-137 inhibitor with AKR1B10 Wt was enhanced, but no significant difference in the AKR1B10 Mut group (Fig. 4F). The results of the RNA Immunoprecipitation (RIP) experiments depicted that the AKR1B10 and miR-137 expression increased significantly in the Anti-Ago2 group compared with the Anti-lgG group (Fig. 4G). Moreover, miR-137 expression was enhanced while AKR1B10 expression was decreased in cells transduced with miR-137 mimic, while opposing tendency was witnessed in cells transduced with miR-137 inhibitor (Fig. 4H, I).

Conclusively, miR-137 targetes and inhibits AKR1B10 expression.

### miR-137 alleviates ethanol-induced hepatocyte damage by inhibiting AKR1B10

**Fig. 5:**
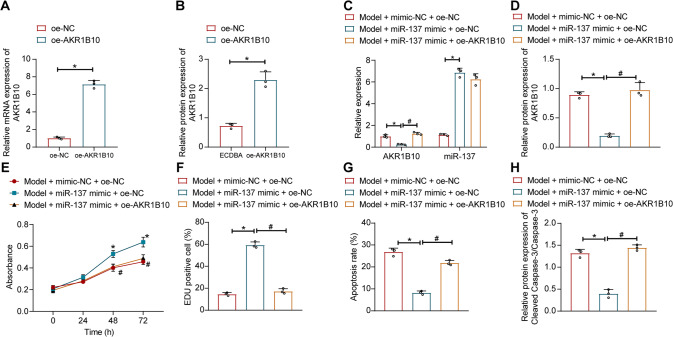
miR-137 reduces ethanol-induced hepatocyte damage by AKR1B10. **A**, **B** AKR1B10 overexpression efficiency in ethanol-treated AML12 cells measured by RT-qPCR and Western blot assay. *p < 0.05 compared to the oe-NC group. **C**, **D** Expression of miR-137 and AKR1B10 in ethanol-treated AML12 cells upon miR-137 mimic and oe-AKR1B10 treatment by RT-qPCR or Western blot assay. *p < 0.05 compared to Model + mimic-NC + oe-NC group; #*p* < 0.05 compared to the Model + miR-137 mimic + oe-NC group. **E** Ethanol-treated AML12 cell viability upon miR-137 mimic and oe-AKR1B10 treatment determined by the CCK-8 experiments. **F** Ethanol-treated AML12 cell proliferation upon miR-137 mimic and oe-AKR1B10 treatment determined by EdU staining. **G** Apoptosis rate in ethanol-treated AML12 cells upon miR-137 mimic and oe-AKR1B10 treatment determined by flow cytometry. **H** Protein expression of Cleaved Caspase-3 and Caspase-3 in ethanol-treated AML12 cells upon miR-137 mimic and oe-AKR1B10 treatment determined by Western blot assay. For **E**–**H**, *p < 0.05 compared to the Model + mimic-NC + oe-NC group; #*p* < 0.05 compared to the Model + miR-137 mimic + oe-NC group. Data are shown as the mean ± standard deviation of three technical replicates. The unpaired *t*-test was conducted for two group comparison, and one-way ANOVA and Tukey’s post-hoc tests for muti-group comparison. Comparison among groups at different time points was completed using two-way ANOVA followed by Bonferroni’s multiple comparison test.

We found above that miR-137 targeted AKR1B10 expression. Here, we further validated that whether miR-137 attenuated ethanol-induced hepatocyte damage by inhibiting AKR1B10 expression. The transfection efficiency of AKR1B10 overexpression was verified by RT-qPCR and Western blot assay (Fig. 5A, B, Supplementary Fig. 2A). Moreover, ethanol-induced AML12 cells were transduced with miR-137 mimic alone or combined with oe-AKR1B10. We found an increase in miR-137 expression and a decline in AKR1B10 expression in ethanol-induced AML12 cells transduced with miR-137 mimic, while further oe-AKR1B10 elevated AKR1B10 expression (Fig. 5C, D, Supplementary Fig. 2B).

Functionally, overexpression of miR-137 enhanced ethanol-induced AML12 cell viability and proliferation, but lowered apoptosis rate, accompanied with reduced expression of Cleaved Caspase-3. However, simultaneous overexpression of miR-137 and AKR1B10 curtailed ethanol-induced AML12 cell viability and proliferation, but facilitated cell apoptosis, along with increased expression of Cleaved Caspase-3 (Fig. 5E–H, Supplementary Fig. 2C–E). No significant difference was witnessed in caspase-3 expression among all groups.

The above results unveil that miR-137 attenuates ethanol-induced hepatocyte damage by limiting AKR1B10 expression.

### 1700020I14Rik suppresses miR-137 expression and in turn upregulates AKR1B10

**Fig. 6:**
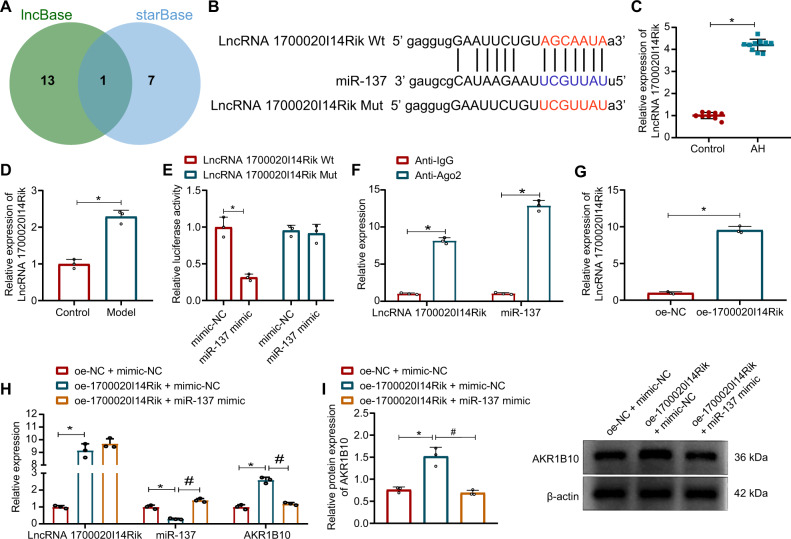
1700020I14Rik promotes AKR1B10 expression by competitive binding of miR-137. **A** Venn plot of the upstream LncRNA of miR-137 obtained by lncBase and starBase prediction. **B** Plot of the binding site of 1700020I14Rik to miR-137 in starBase. **C** 1700020I14Rik expression in the liver tissues of AH mice (*n* = 10) determined by RT-qPCR. *p < 0.05 compared to the control group. **D** 1700020I14Rik expression in the ethanol-treated AML12 cells determined by RT-qPCR. *p < 0.05 compared to the control group. **E** Dual-luciferase reporter assay detected the target relation between 1700020I14Rik and miR-137. *p < 0.05 compared to the mimic-NC group. **F** Interation between 1700020I14Rik and miR-137 detected by RIP. *p < 0.05 compared to the Anti-lgG group. **G** 1700020I14Rik overexpression efficiency measured by RT-qPCR. *p < 0.05 compared to the oe-NC group. **H**, **I** 1700020I14Rik, miR-137 and AKR1B10 expression in AML12 cells upon oe-1700020I14Rik or miR-137 mimic treatment by RT-qPCR or Western blot assay. *p < 0.05 compared to the oe-NC + mimic-NC group; #*p* < 0.05 compared to the oe-1700020I14Rik + mimic-NC group. Data are shown as the mean ± standard deviation of three technical replicates. The unpaired *t*-test was conducted for two group comparison, and one-way analysis of variance and Tukey’s post-hoc tests for muti-group comparison.

To further investigate the upstream regulatory mechanism of miR-137, the upstream lncRNAs of miR-137 were predicted by lncBase and starBase, obtaining 14 and 8 lncRNAs, respectively, and through the Venn diagram, we found that the intersection of the two results showed only one lncRNA, lncRNA 1700020I14Rik (Fig. 6A). Figure 6B displays the binding site. We confirmed that 1700020I14Rik expression was increased in the liver tissues of AH mice and ethanol-induced AML12 cells (Fig. 6C, D).

Also, the luciferase activity of co-transduction of miR-137 mimic and 1700020I14Rik Wt was significantly reduced, while no significant difference was seen upon co-transduction of miR-137 mimic and 1700020I14Rik Mut, as revealed by luciferase assay (Fig. 6E). RIP assay noted that 1700020I14Rik and miR-137 expression also increased in the Anti-Ago2 group compared with the Anti-lgG group (Fig. 6F).

We then constructed the 1700020I14Rik overexpression sequence, and 1700020I14Rik expression was enhanced by the treatment of oe-1700020I14Rik (Fig. 6G). Overexpression of 1700020I14Rik also increased expression of both 1700020I14Rik and AKR1B10 but decreased miR-137 expression. In contrast, the simultaneous overexpression of 1700020I14Rik and miR-137 elevated expression of miR-137 but reduced AKR1B10 expression (Fig. 6H, I).

The above results demonstrate that 1700020I14Rik inhibits miR-137 and in turn promotes AKR1B10 expression.

### 1700020I14Rik increases ethanol-induced hepatocyte damage by promoting the AKR1B10 expression and Erk pathway

**Fig. 7:**
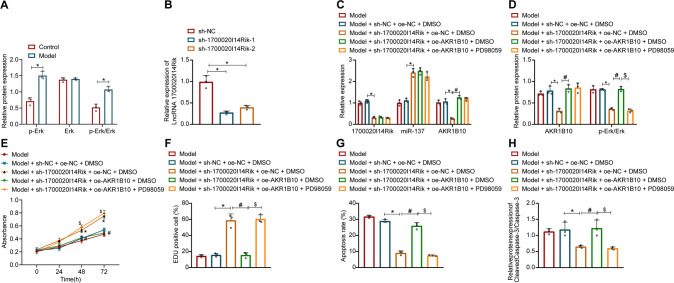
1700020I14Rik induces ethanol-induced hepatocyte damage *via* the AKR1B10 and Erk pathways. **A** Western blot assay was used to detect the expression of Erk and p-Erk in liver tissues. *p < 0.05 compared with the control group. **B** Silencing efficiency of 1700020I14Rik measured by RT-qPCR. *p < 0.05 compared with the sh-NC group. **C**, **D** Expression of 1700020I14Rik, AKR1B10, Erk, and p-Erk in ethanol-induced AML12 cells upon sh-1700020I14Rik, oe-AKR1B10 or PD98059 treatment determined by RT-qPCR or Western blot assay. **E** Ethanol-induced AML12 cell viability upon sh-1700020I14Rik, oe-AKR1B10 or PD98059 treatment determined by the CCK-8 experiments. **F** Ethanol-induced AML12 cell proliferation upon sh-1700020I14Rik, oe-AKR1B10 or PD98059 treatment was determined by EdU staining. **G** Apoptosis rate in ethanol-induced AML12 cells upon sh-1700020I14Rik, oe-AKR1B10 or PD98059 treatment determined by flow cytometry. **H** Expression of Cleaved Caspase-3 and Caspase-3 in ethanol-induced AML12 cells upon sh-1700020I14Rik, oe-AKR1B10 or PD98059 treatment determined by Western blot assay. For **C**–**H**, *p < 0.05 compared to the Model + sh-NC + oe-NC + DMSO group; #*p* < 0.05 compared to the Model + sh-1700020I14Rik + oe-NC + DMSO group; $*p* < 0.05 compared to the Model + sh-1700020I14Rik + oe-AKR1B10 + DMSO group. Data are shown as the mean ± standard deviation of three technical replicates. The unpaired *t*-test was conducted for two group comparison, and one-way analysis of variance and Tukey’s post-hoc tests for muti-group comparison.

Further validation of the 1700020I14Rik/miR-137/AKR1B10/Erk axis on ethanol-induced hepatocyte damage was performed. In ethanol-treated AML12 cells, p-Erk expression was increased, yet Erk expression exerted no alteration (Fig. 7A, Supplementary Fig. 3A).

Both treatment of the established sh-1700020I14Rik-1 and sh-1700020I14Rik-2 reduced expression of 1700020I14Rik in ethanol-induced AML12 cells (Fig. 7B), and the sh-1700020I14Rik-1 was more efficient in silencing effects and was used for subsequent experiments. After silencing of 1700020I14Rik, expression of 1700020I14Rik, AKR1B10, and p-Erk was significantly reduced in ethanol-induced AML12 cells. However, the further overexpression of AKR1B10 increased miR-137 expression and p-Erk level in ethanol-induced AML12 cells. Relative to sh-1700020I14Rik + oe-AKR1B10 + DMSO treatment, sh-1700020I14Rik + oe-AKR1B10 + PD98059 treatment brought about a decline in p-Erk level in ethanol-induced AML12 cells (Fig. 7C, D, Supplementary Fig. 3B). There was no significant difference in Erk expression following all treatment in ethanol-induced AML12 cells.

Further, ethanol-induced AML12 cell viability and proliferation were significantly increased and cell apoptosis was reduced, as well as Cleaved Caspase-3 expression was downregulated by sh-1700020I14Rik, while further transduction of oe-AKR1B10 caused opposite findings. Relative to sh-1700020I14Rik + oe-AKR1B10 + DMSO treatment, sh-1700020I14Rik + oe-AKR1B10 + PD98059 treatment brought about an enhancement in ethanol-induced AML12 cell viability and proliferation but a reduction in apoptosis and Cleaved Caspase-3 expression (Fig. 7E–H, Supplementary Fig. 3C–E).

Therefore, 1700020I14Rik increases ethanol-induced hepatocyte damage by promoting the AKR1B10/Erk axis.

### 1700020I14Rik promotes liver injury in AH mice by promoting the AKR1B10/Erk pathway

**Fig. 8:**
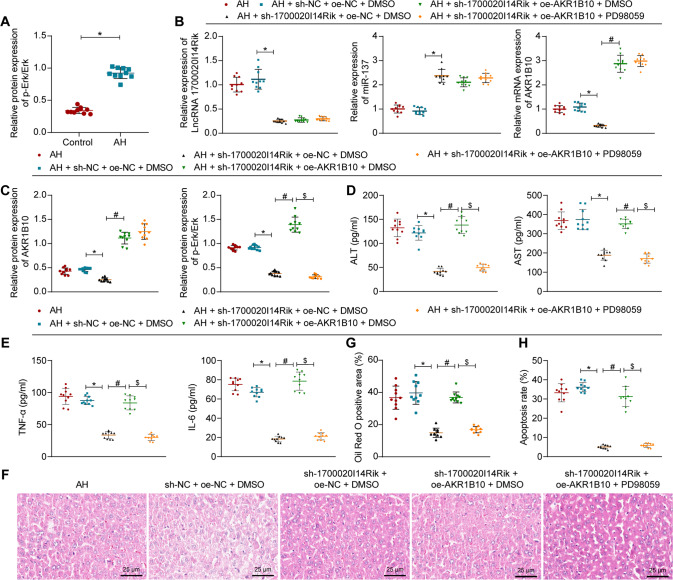
1700020I14Rik induces liver injury in AH mice *via* the AKR1B10/Erk pathway. **A** Western blot assay was used to measure the expression of Erk and p-Erk in liver tissues of AH mice (*n* = 10). *p < 0.05 compared to the control group. **B**, **C** Expression of 1700020I14Rik, AKR1B10, Erk, and p-Erk in liver tissues of AH mice upon sh-1700020I14Rik, oe-AKR1B10 or PD98059 treatment determined by RT-qPCR or Western blot assay. **D**, **E** Serum levels of ALT, AST, TNF-α, and IL-6 in serum of AH mice ice upon sh-1700020I14Rik, oe-AKR1B10 or PD98059 treatment as ELISA detected. **F** Liver damage in mice upon sh-1700020I14Rik, oe-AKR1B10 or PD98059 treatment as H&E staining measured (400×). **G** Lipid drops in liver tissues of AH mice upon sh-1700020I14Rik, oe-AKR1B10 or PD98059 treatment by oil red O staining. **H** Number of apoptotic cells in liver tissues of AH mice upon sh-1700020I14Rik, oe-AKR1B10 or PD98059 treatment determined by TUNEL staining (*n* = 10). For **B**–**I**, *p < 0.05 compared to the AH + sh-NC + oe + NC-DMSO group; #*p* < 0.05 compared to the AH + sh-1700020I14Rik + oe-NC + DMSO group; $*p* < 0.05 compared with the AH + sh-1700020I14Rik + oe-AKR1B10 + DMSO group. Data are shown as the mean ± standard deviation. The unpaired *t*-test was conducted for two group comparison, and one-way analysis of variance and Tukey’s post-hoc tests for muti-group comparison.

Finally, in vivo study concerning on the effect of 1700020I14Rik/AKR1B10/Erk axis on AH mice was carried out. AH mice showed elevated p-Erk level and no alteration in Erk expression in liver tissues (Fig. 8A, Supplementary Fig. 4A). Expression of 1700020I14Rik, AKR1B10, and p-Erk level was greatly reduced by sh-1700020I14Rik, along with elevated expression of miR-137 in liver tissues of AH mice. But after simultaneous treatment of sh-1700020I14Rik and oe-AKR1B10, AKR1B10 expression and p-Erk level were increased, while further application of PD98059 lowered p-Erk level in liver tissues of AH mice. There were no significant differences in Erk expression in liver tissues of AH mice following all treatments (Fig. 8B, C, Supplementary Fig. 4B).

Moreover, as ELISA results shown, serum levels of ALT, AST, TNF-α, and IL-6 in serum of AH mice were reduced in response to sh-1700020I14Rik, but opposing trends were seen after further overexpression of AKR1B10. Serum levels of ALT, AST, TNF-α, and IL-6 were then decreased in serum of AH mice after further application of PD98059 in the presence of sh-1700020I14Rik and oe-AKR1B10 (Fig. 8D, E).

Meanwhile, liver injury, infiltration of inflammatory cells, lipid droplets, and number of apoptotic cells were reduced in liver tissues of AH mice after silencing of 1700020I14Rik, which were reversed upon simultaneous sh-1700020I14Rik + oe-AKR1B10 treatment. Less liver injury, infiltration of inflammatory cells, lipid droplets, and number of apoptotic cells occurred in liver tissues of AH mice after further application of PD98059 in the presence of sh-1700020I14Rik and oe-AKR1B10 (Fig. 8F–H, Supplementary Fig. 4C, D).

The aforementioned results suggest that 1700020I14Rik promotes liver injury in AH mice by promoting the AKR1B10/Erk axis.

## Discussion

It has been suggested that lncRNAs play critical roles in tumorigenesis of different diseases, whereas only few of them have been well functionally characterized [17]. Specifically, lncRNAs are proven to be implicated in the progression of AH [18]. In this study, we reported the identification of a novel lncRNA 1700020I14Rik, which was highly expressed in AH tissues and ethanol-induced cells. Bioinformatics analysis using public databases also suggested that 1700020I14Rik might facilitate AH progression by regulating hepatocytes. Accordingly, we proposed oncogenic roles for 1700020I14Rik in AH.

At first, we demonstrated that AKR1B10 was highly expressed in AH mice and ethanol-induced cells, and silenced AKR1B10 relieved liver injury in vivo and reduced ethanol-induced hepatocyte damage in vitro. Increasing evidence unveiled that AKR1B10 was induced in many cancer tissues and shared correlation with tumor progression and some noncanceraous diseases [19]. Similar to our results, overexpression of AKR1B10 induced urinary bladder cancer cell aggressiveness, whereas inhibition of AKR1B10 by siRNA or oleanolic acid could exert the opposite effects [20]. Moreover, AKR1B10 shows close association with the immune cell infiltrations and in non-alcoholic fatty liver disease [21]. AKR1B10 also functions as an oncogene in HCC [22]. As our experimental results shown, silenced AKR1B10 by sh-AKR1B10 increased cell viability and proliferation, but decreased apoptosis, accompanied with reduced expression of Cleaved Caspase-3. AKR1B10 silencing resulted in caspase-3-mediated apoptosis of colon carcinoma cells and lung carcinoma cells [23]. The ethanol-induced cell model is frequently used for simulation of AH in vitro [24]. Also, in AH modeled mice, silenced AKR1B10 decreased serum levels of ALT, AST, TNF-α, and IL-6. It has been found that the concentrations of inflammatory factors TNF-α, IL-1beta, and IL-6 were induced by alcohol treatment [25]. Similarly, pharmacological inhibitors of AKR1B10 were capable of reducing steatosis, fibrosis, and inflammation in mouse with non-alcoholic steatohepatitis [26]. Taken together, we could demonstrate that AKR1B10 played an oncogenic role in AH, and silenced AKR1B10 could relieve AH both in vitro and in vivo.

We defined through multiple assays that AKR1B10 could be targeted by miR-137. AKR1B10P1, an isoform pseudogene of oncogenic AKR1B10, is validated as a down-stream target degraded by tumor-suppressing miR-138, and there existed a positive feedback from AKR1B10P1, by which miR-138 interacts with AKR1B10P1 via a ceRNA approach in HCC [27]. This finding proved the possibility that AKR1B10 was able to target miRNA. As our results shown, miR-137 was poorly expressed in AH. miR-137 shared correlation with hepatic lipid metabolism [28]. In line with our results reporting that miR-137 was able to relieve the hepatocyte damage, overexpression of miR-137 exerted inhibitory function in HCC cell proliferation [29]. We then screened the upstream lncRNA of miR-137, and found that 1700020I14Rik suppressed miR-137 expression. Likewise, 1700020I14Rik could negatively regulate the expression of miR-297a in myocardial cell injury [30]. However, to our best knowledge, little study has reported the relationship between 1700020I14Rik and miR-137 before.

In addition, the AKR1B10-ERK signaling pathway was abnormally expressed in lung adenocarcinoma [15]. AKR1B10 could activate ERK signaling pathway in various cancer cells [14, 20, 31]. ERK1/2 signaling posseses great potential in controlling cell proliferation by phosphorylation of mutiple down-stream factors [32]. Partly in line with our study, IL-20 aggravated AH through activation of ERK/p38MAPK/NRF2 signaling pathways [33]. More importantly, crRNA networks (circRNA-miRNA-mRNA) have been widely constructed for disease and cancer treatment [34]. With the documents discussed above, we believed that 1700020I14Rik could suppress miR-137 expression, which activates AKR1B10-Erk pathway in AH.

**Fig. 9:**
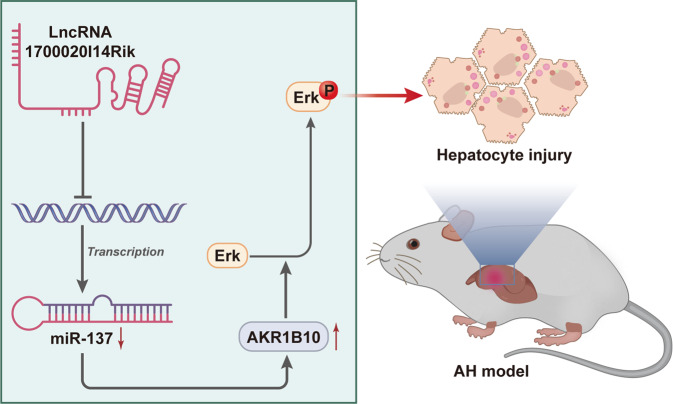
Schematic schematic for 1700020I14Rik-based molecular mechanisms affecting hepatocyte injury in mice with AH via the miR-137/AKR1B10/Erk pathway. 1700020I14Rik can competitively regulate miR-137-mediated AKR1B10 to activate the Erk pathway, thus eventually inducing hepatocyte damage in AH mice.

In summary, we reported deteriorative roles for 1700020I14Rik in AH. Our results indicated that 1700020I14Rik was overexpressed in AH. Moreover, 1700020I14Rik reinforced inflammatory reaction in vivo and enhanced hepatocyte damage in vitro, through functioning as a ceRNA for miR-137 and subsequently activating the AKR1B10-ERK signaling pathway (Fig. 9), therefore emphasizing the potentials of this axis in AH diagnosis and therapy. However, we only explored the single dose of alcohol in AKR1B10 expression in AML12 cells, and our future studies would further investigate whether alcohol could increase AKR1B10 expression in dose-dependent manner and time-dependent manner.

## Materials and methods

### Bioinformatics analysis

Through the GEO database, the AH patient-related microarray GSE28619 (seven normal control samples, 15 AH samples) from the annotation platform GPL570 [HG-U133_Plus_2] Affymetrix Human Genome U133 Plus 2.0 Array was downloaded. Differential expression analysis was implemented for sorting the differential expressed genes (DEGs) of AH employing the R language limma package with │logFoldChange (FC)│ > 3 and *p* < 0.01 as the threshold. Whether the gene was similarly expressed in mice was determined with the Gene retrieval function of NCBI. The genes with the most significant differential expression were selected for determining down-stream regulatory pathways as established by the existing literature. Online prediction websites miRDB (Target Score > 70), DIANA TOOLS, and RAID were selected for predicting upstream miRNAs of mouse genes. The key upstream miRNAs were screened by mapping Venn plots, and the targeted binding relationships were confirmed by binding site maps. AH-related microarray GSE59492 from annotation platform GPL16384 [miRNA-3] Affymetrix Multispecies miRNA-3 Array was downloaded from the GEO database, and the critical miRNA expression was determined by miRNA data from microarray GSE59492. By the online prediction websites lncBase (liver-related) and StarBase, the upstream lncRNAs of key miRNA were predicted respectively. After intersection, the Venn plot was constructed for collection of the upstream lncRNAs.

### Establishment of an AH mouse model

Eighty healthy male C57BL/6 J mice aged 6–8 weeks were commercially available from Vital River (219, Beijing, China). All mice were initially fed *ad libitum* with Lieber-DeCarli feed (Lieber-C, Dyets Biotechnology, Wuxi, China) for 5 days for acclimation. The mice fed with ethanol-free Lieber-DeCarli liquid feed were used as the control. The other mice were subjected to ethanol with gradually increased concentration of 2–4% (v/v) at 2 weeks before modeling, and when the ethanol concentration reached 4%, the modeling was started, during which the change of feeding and body weight were recorded. After 12 weeks, serum ALT and AST levels increased, and severe liver damage was observed, with significant inflammatory cell infiltration, enhanced lipid droplets, and obvious hepatocyte apoptosis detected, then the modeling was ended. Mice were placed in closed euthanasia devices (RC-100, Yuyan Bio, Shanghai, China), and the CO_2_ exchange rate was 30% of the container volume per minute to ensure that the mice were unconscious before pain. Serum and liver tissue specimens were collected when mice were fully euthanized.

Mice were differently treated with 10 mice in each treatment. Except for the mice as control, AH mice were injected with the lentivirus expressing silenced AKR1B10 (sh-AKR1B10) and silenced 1700020I14Rik (sh-1700020I14Rik), the lentivirus overexpressing AKR1B10 (oe-AKR1B10), their relevant negative control (oe-NC and sh-NC), DMSO, and/or PD98059. The lentivirus silencing vector used for mice were packaged using the core plasmid (PLKO.1) inserted into the target gene silencing sequences and auxiliary plasmids (RRE, REV, Vsvg). The lentivirus overexpression vector was packaged by the core plasmid (Fugw-GFP, Plx304) inserted into the complementary DNA (cDNA) of genes to be tested and auxiliary plasmids (RRE, REV, Vsvg). The Erk1/2 inhibitor (PD98059, HY-12028, MedChemExpress) was dissolved in DMSO. Before the modeling, one time of intraperitoneal injection of 10 mg/kg PD98059 was performed, while mice received 100 μL lentivirus (Han Bio, Shanghai) injection with titers of 1 × 10^9^ TU/mL *via* the tail vein. The primer sequences and plasmid constructs are also completed by Han Bio (Supplementary Table 1). The experimental procedures and the animal use protocol were approved by the Animal Ethics Committee of Vital River (Beijing, China).

### IF

The paraffin-embedded sections were blocked with normal goat serum blocking solution (E510009, Sangon Biotechnology, Shanghai, China) for 20 min at room temperature, followed by reaction with diluted primary antibodies of rabbit anti-AKR1B10 (OAGA00776, 1: 1000, Aviva Systems Biology) and chicken anti-albumin (ab106582,1:100, Abcam, Cambridge, UK) at 4 °C overnight. After washing with PBS, the sections were incubated with secondary antibody IgG (goat anti-chicken, ab150176; goat anti-rabbit, ab150077, 1:200, Abcam) with different fluorescent labels at room temperature for 1 h. After stained with 4',6-diamidino-2-phenylindole (DAPI; D9542, Sigma-Aldrich, St. Louis, MO, USA) for 10 min, the sections were observed under an upright microscope (BX63, Olympus, Japan). Five visual fields were taken from each section, and the number of green positive cells and cells co-stained with green and red were calculated respectively, positive rate = number of cells co-stained with green and red/number of green positive cells × 100%.

### ELISA

Serum levels of ALT, AST, TNF-α, and IL-6 were detected in line with the kit instructions of the mouse ALT ELISA kit (ab282882, Abcam), mouse AST ELISA kit (ab263882, Abcam), mouse TNF-α ELISA kit (ab208348, Abcam), and mouse IL-6 ELISA Kit (ab222503, Abcam).

### Observation of pathological changes

The 4% formalin-fixed liver tissues were parraffin-embedded and cut into 5 µm serial sections, which were stained with the hematoxylin-eosin (H&E) staining kit (C0105S, Beyotime) [35]. Frozen mouse liver tissue sections were taken and processed with oil red O staining kit (C0158S, Beyotime Biotechnology) to detect the fat of mouse liver tissues [36]. The pathological changes were estimated under the microscope (BX63, Olympus, Japan).

### TUNEL staining

This experiment was carried out with the apoptosis detection kit (C1098, Beyotime Biotechnology). After treated with DAPI, the sections were observed under the microscope (BX63, Olympus) with apoptosis rates counted.

### Cell culture and transduction

The mouse normal hepatocyte line AML12 was procured from the Biobw China Microbiology Seed Query Network (bio-107450) and cultured in a dedicated medium (bio-54292, Biobw). AML12 cells were seeded in the culture flask with a density of 5 × 10^4^ cells/mL. The next day, the cells for modeling were treated with 50 mM ethanol for 24 h to establish the ethanol-induced liver injury model, followed by the subsequent addition of lentivirus. The cells as control were cultured with basal medium [37]. PD98059 was dissolved in DMSO at a concentration of 1 mM and stored at −80 °C, and diluted to 20 μM to treat cells for 24 h [38].

HEK293T cells (CC-Y1010) were cultured in the Dulbecco’s modified eagle medium (10569044, GIBCO) replenished with 10% fetal bovine serum and 1% penicillin-streptomycin (15070063, GIBCO) in the incubator at 37 °C and 5% CO_2_.

AML12 cells were transduced with lentivirus-based short hairpin RNA against AKR1B10-1 (sh-AKR1B10-1), sh-AKR1B10-2, sh-1700020I14Rik-1, and sh-1700020I14Rik-2, lentivirus overexpressing 1700020I14Rik (oe-1700020I14Rik), oe-AKR1B10-1, plasmids of miR-137 mimic, miR-137 inhibitor, relevant NC, DMSO, and/or PD98059. The virus titer of lentivirus (Han Bio) was 1 × 10^9^ TU/mL. The cells were treated with 100 μL/mL lentivirus for 6 h, and the culture continued after renewal of solution. The primer sequences and plasmid constructs are constructed by Han Bio (Supplementary Table 2). The plasmids of mimic-NC and miR-137 mimic were procured from Ribo Biotechnology (Guangzhou). Puromycin (60210ES25, YEASEN, Shanghai, China) was used for screening of the stably transduced cell lines.

### RT-qPCR

With Trizol (16096020, Thermo Fisher Scientific), total cellular RNA was extracted from AML12 cells and its concentration and purity were assayed by a NanoDrop One/OneC trace nucleic acid protein concentration meter (A260/A280 = 2.0; concentration > 5 μg/μL) from Thermo Scientific. For mRNA, the cDNA first-strand synthesis kit (D7168L, Beyotime) was utilized, for miRNA, the miRNA first-strand cDNA synthesis (plus tail method) kit (B532451, Sangon) was adopted.

RT-qPCR kit (Q511-02, Vazyme Biotech, Nanjing, China) was applied for the RT-qPCR experiments. PCR amplification was made at Bio-rad real time quatitative PCR equipment CFX96. LncRNA1700020I14Rik and AKR1B10 levels were measured with β-actin as normalizer while U6 served as the normalizer for miR-137. Primer sequences are designed and provided by Sangon Biotech (Supplementary Table 2). The 2^−ΔΔCt^ method was applied for analysis.

### Western blot assay

The extracts from AML12 cells and liver tissues were subjected to electrophoresis separation. Then, the extracts on the gel were transferred to the polyvinylidene fluoride membrane (1620177, BIO-RAD) which was blocked with 5% skim milk or 5% BSA for 1 h at ambient temperature. The membrane was incubated with primary antibodies of rabbit anti-β-actin (4970, 1:5000, Cell Signaling Technology), rabbit anti-AKR1B10 (OAGA00776, 1:1000, Aviva Systems Biology), rabbit anti-Caspase-3 (ab184787, 1:1000, Abcam), rabbit anti-Cleaved Caspase-3 (9661, 1:1000, Cell Signaling Technology), rabbit anti-phosphorylated (p)-Erk1/2 (4370, 1:1000, Cell Signaling Technology), and rabbit anti-Erk1/2 (4695, 1:1000, Cell Signaling Technology) at 4 °C overnight. The next day, the membrane was incubated with horseradish peroxide labeled goat anti-rabbit IgG (ab6721, 1:5000, Abcam) secondary antibody for l h at ambient temperature. The membrane was immersed in the enhanced chemiluminescence reaction solution (1705062, BIO-RAD) and imaged utilizing the Image Quant LAS 4000 C Gel Imager (GE, USA).

### Cell counting kit (CCK)-8

AML12 cell proliferation experiments were processed utilizing the CCK-8 kit (C0037, Beyotime). After 0 h, 24 h, 48 h, and 72 h of different treatments, the AML12 cells were added with 10 μL CCK-8 reagent for incubation for 1 h. Absorbance was recorded at 450 nm using Varioskan LUX, a multifunctional microplate reader, followed by construction of cell proliferation curves.

### 5-Ethynyl-2'-deoxyuridine (EdU)

AML12 cell proliferation was assayed using the EdU cell proliferation Kit (C0075S, Beyotime Biotechnology). Following the Hoechst 33342 staining for 10 min, cells were observed and photographed under a microscope (BX63, Olympus) with five fields of view taken randomly from each sample for counting EdU-positive cell rates.

### Flow cytometry

After different treatments, the AML12 cells were detached and centrifuged with supernatant removed. Then, AML12 cell apoptosis was assayed with the Annexin V-fluorescein isothiocyanate (FITC) and propidium iodide (PI) kit (C1062L, Beyotime) with an Attune NxT flow cytometer (Thermo Fisher Science).

### Dual-luciferase reporter gene assay

The AKR1B10 mRNA 3'untranslated region (UTR) gene fragment as AKR1B10 Wt and the mutant fragment as AKR1B10 Mut as well as the 1700020I14Rik gene fragment (1700020I14Rik Wt) and the mutated fragment (1700020I14Rik Mut) specifically bound to miR-137 were synthesized, respectively, and co-transfected into HEK293T cells with 50 nM mimic-NC, miR-137 mimic, inhibitor-NC, miR-137 inhibitor, respectively with Lipofectamine 3000 (L3000001, Thermo Fisher science) transfection reagent. After transfection for 48 h, the Dual-Luciferase^®^ Repor-terAssay System kit (E1910, Promega) was adopted for assaying the luciferase activity on the GloMax^®^ 20/20 Luminometer Tester (E5311, Promega). All vectors were constructed Sangon Biotechnology.

### RIP

The RIP kit (RIP-12RXN, Sigma-Aldrich) was used to detect the binding of miR-137, AKR1B10, and 1700020I14Rik to AGO2 protein according to the instructions [39]. The antibodies used for RIP are: AGO2 (1:100, ab32381, Abcam), and IgG (1:100, ab200699, Abcam, NC).

### Statistical analysis

Statistical analysis was finished using SPSS software (version 21.0, IBM, USA). Measurement data are described as mean ± standard deviation. The unpaired *t*-test was conducted for two group comparison, and one-way analysis of variance (ANOVA) and Tukey’s post-hoc tests for muti-group comparison. Comparison among groups at different time points was completed using two-way ANOVA followed by Bonferroni’s multiple comparison test. Statistical differences were validated by a *p* < 0.05.

## Supplementary information

Supplementary Figures

Supplementary Tables

Original Images

## Data Availability

The datasets generated and/or analysed during the current study are available in the manuscript and supplementary materials.
