# Supplementary material for: LncRNA 1700020I14Rik promotes AKR1B10 expression and activates Erk pathway to induce hepatocyte damage in alcoholic hepatitis
Source: Cell Death Discov. 2022 Aug 26;8:374. doi: 10.1038/s41420-022-01135-w (PMC9418154; doi:10.1038/s41420-022-01135-w)

**Supplementary Figure 1** **Representative images of Figure 2** (A, Figure 2B. B, Figure 2D. C, Figure 2F. D, Figure 2G. E, Figure 2H).


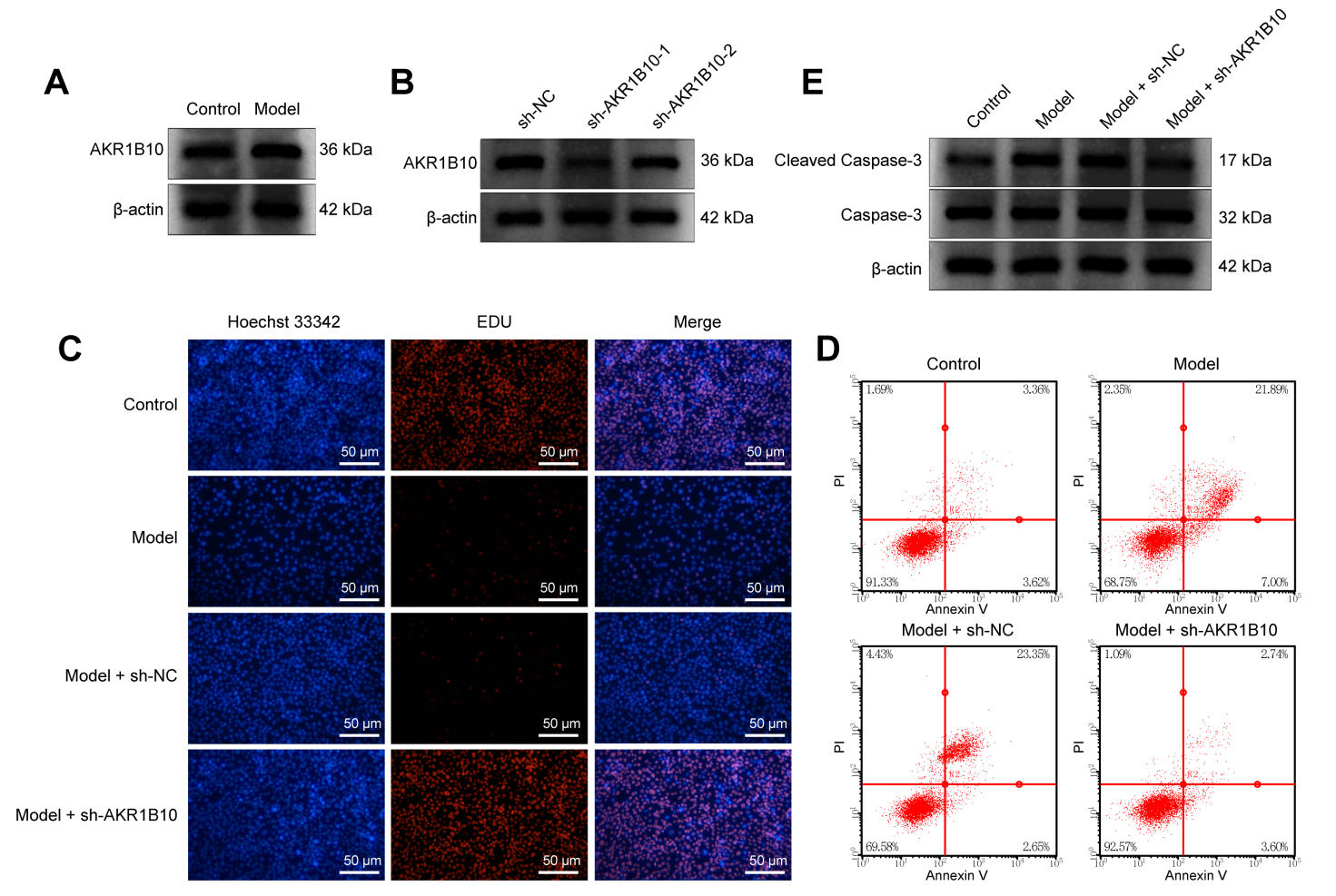


**Supplementary Figure 2** **Representative images of Figure 5** (A, Figure 5B. B, Figure 5D. C, Figure 5F. D, Figure 5G. E, Figure 5H).


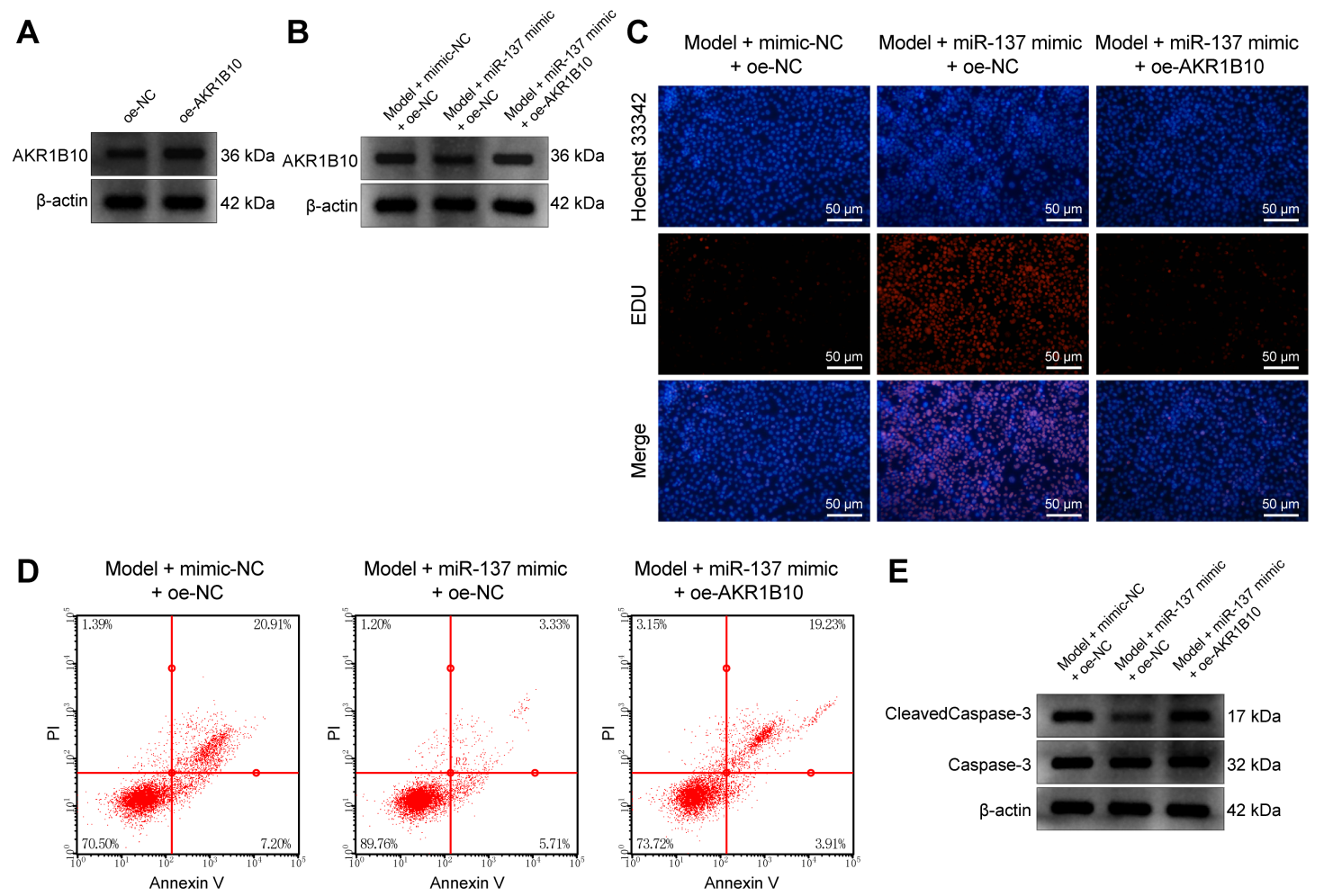


**Supplementary Figure 3** **Representative images of Figure 7** (A, Figure 7A. B, Figure 7D. C, Figure 7F. D, Figure 7G. E, Figure 7H).


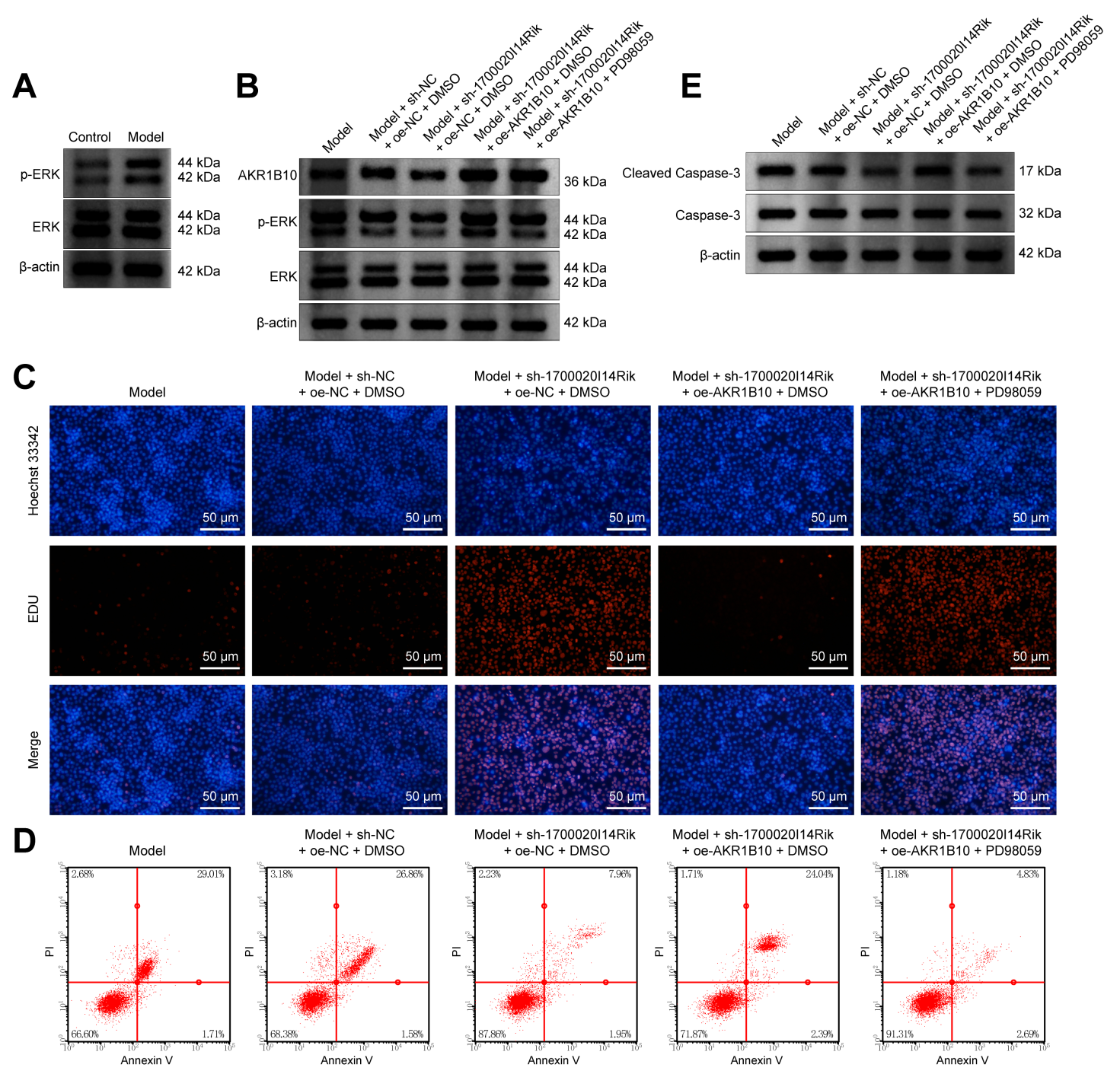


**Supplementary Figure 4** **Representative images of Figure 8** (A, Figure 8A. B, Figure 8C. C, Figure 8G. D, Figure 8H).


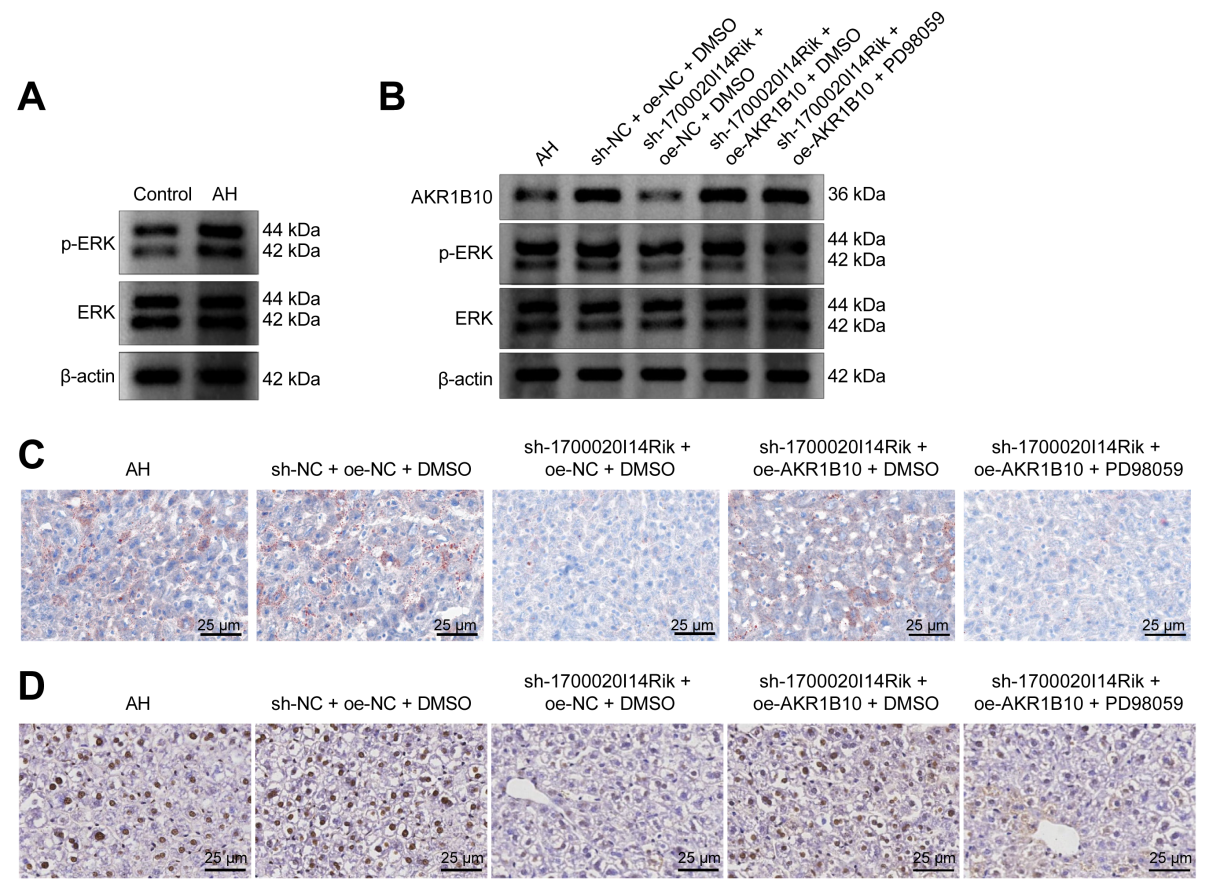

Supplement: Supplementary file 1 — Supplementary Figures [file 41420_2022_1135_MOESM1_ESM.docx]
