# Supplementary material for: LncRNA 1700020I14Rik promotes AKR1B10 expression and activates Erk pathway to induce hepatocyte damage in alcoholic hepatitis
Source: Cell Death Discov. 2022 Aug 26;8:374. doi: 10.1038/s41420-022-01135-w (PMC9418154; doi:10.1038/s41420-022-01135-w)
Supplement: Supplementary file 2 — Supplementary Tables [file 41420_2022_1135_MOESM2_ESM.docx]

**Supplementary Table 1 Silencing sequences for targeted genes**

|  | Primers sequences (5’-3’) |
| --- | --- |
| sh-NC | CCTAAGGTTAAGTCGCCCTCG |
| sh-AKR1B10-1 | CGCTCCTACTGACTCCTATTT |
| sh-AKR1B10-2 | CTTCCCTTTCCATGCGGAATA |
| sh-1700020I14Rik-1 | GAGTACAAGCCAGCTACCAT |
| sh-1700020I14Rik-2 | GCTTGGACATCAAGAGGCTC |

**Supplementary Table 2 Primer sequences for RT-qPCR**

|  | Primer sequences (5’-3’) |
| --- | --- |
| LncRNA1700020I14Rik | F: TAGGCTGGGACAATGCTCAC |
|  | R: CCAACCCAGTCTCACATGCT |
| miR-137 | F: ACGGGTATTCTTGGGTGGATAAT |
|  | R: reverse universal primer |
| AKR1B10 | F: CAAACCAGCCTCAACAGTGC |
|  | R: AACATCCGGCCAGGTCAAAT |
| β-actin | F: GAGCGCAAGTACTCTGTGTG |
|  | R: GGGTGTAAAACGCAGCTCAGTA |
| U6 | F: CTCGCTTCGGCAGCACA |
|  | R: reverse universal primer |

Notes: F, forward; R, reverse
