# Supplementary material for: LncRNA 1700020I14Rik promotes AKR1B10 expression and activates Erk pathway to induce hepatocyte damage in alcoholic hepatitis
Source: Cell Death Discov. 2022 Aug 26;8:374. doi: 10.1038/s41420-022-01135-w (PMC9418154; doi:10.1038/s41420-022-01135-w)
Supplement: Supplementary file 3 — Original Images [file 41420_2022_1135_MOESM3_ESM.docx]

**Original uncropped images of the western blotting**

**Figure 3B**

**
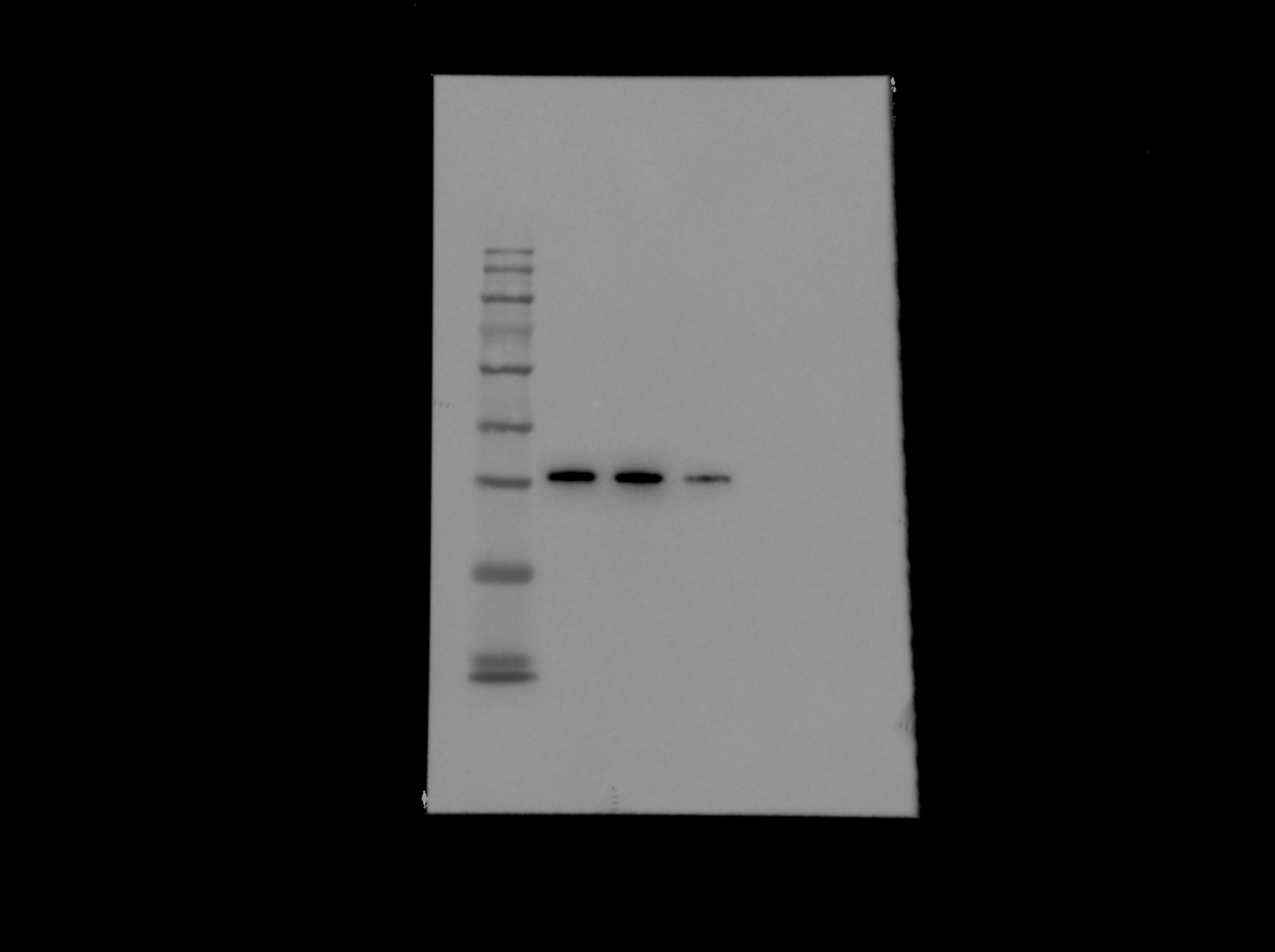
**

**
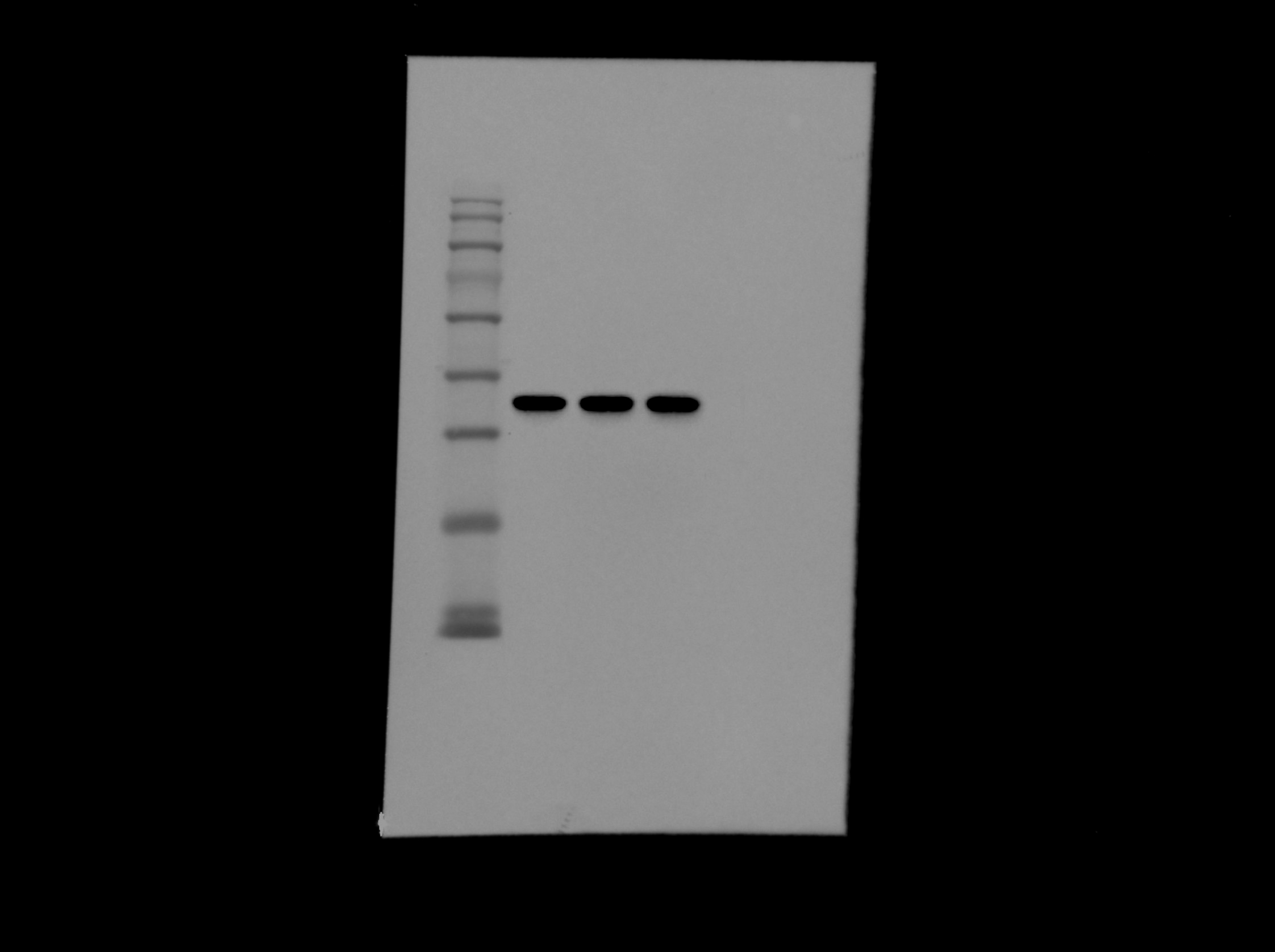
**

**Figure 4I**

**
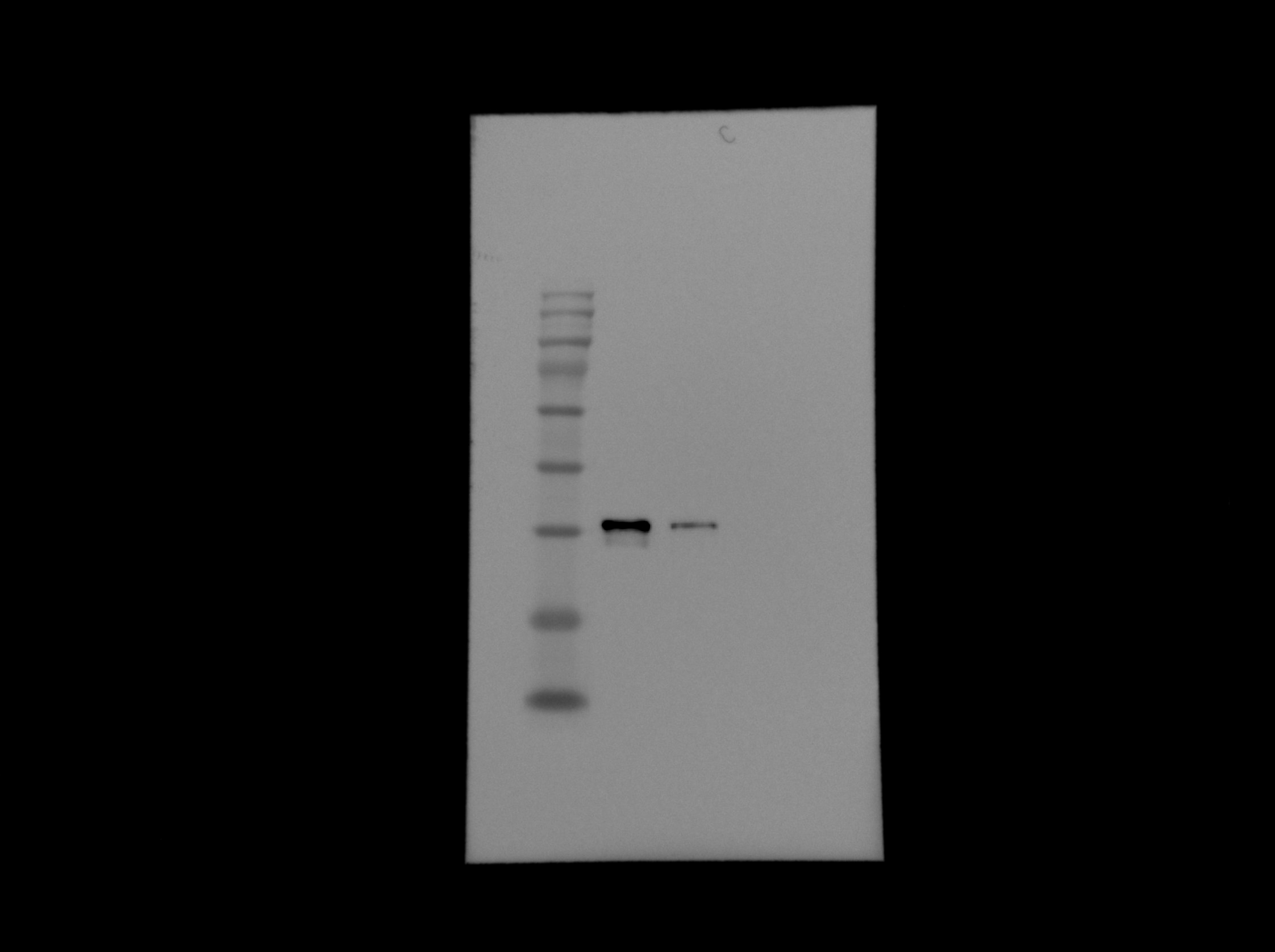
**

**
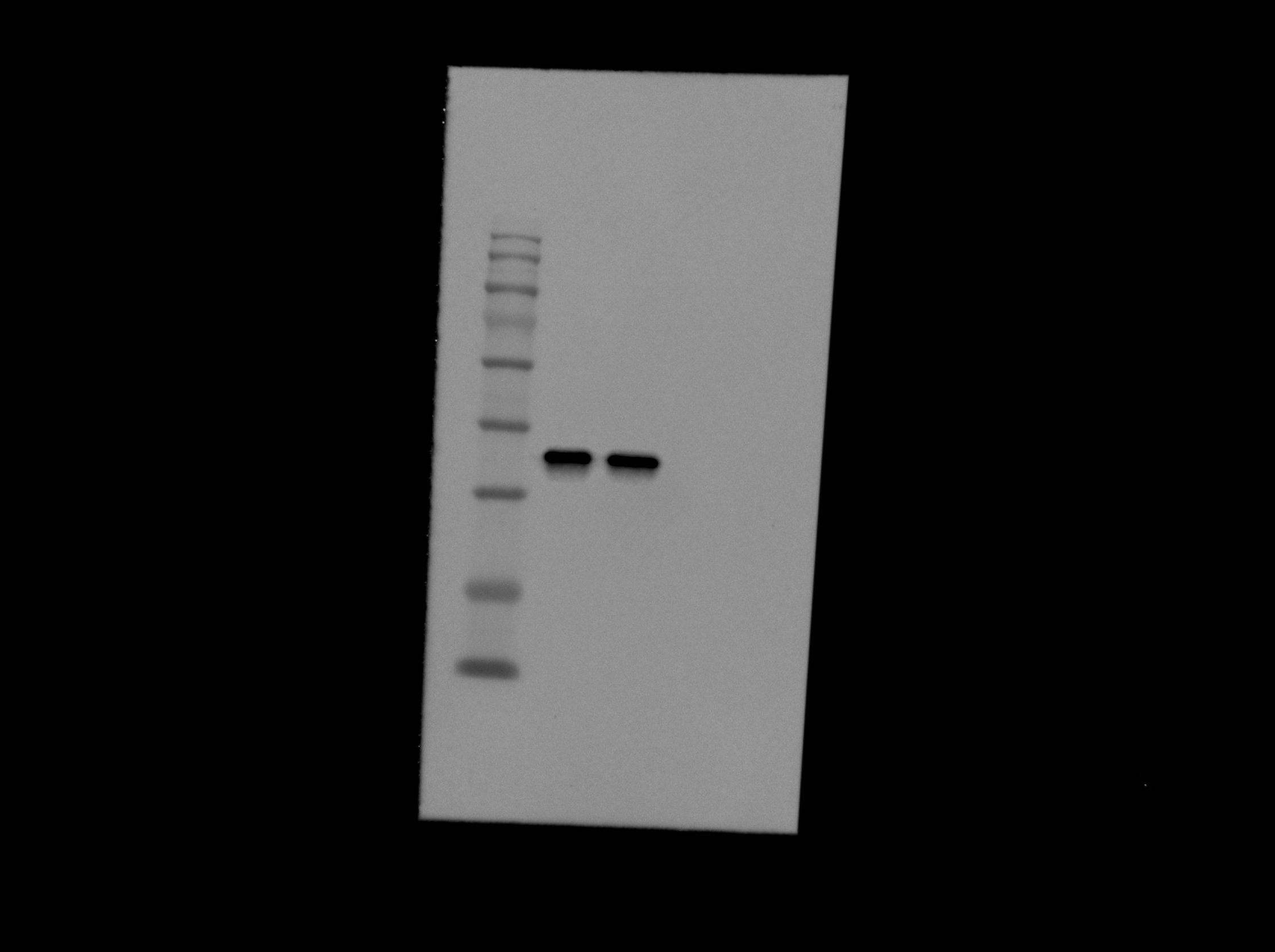
**

**
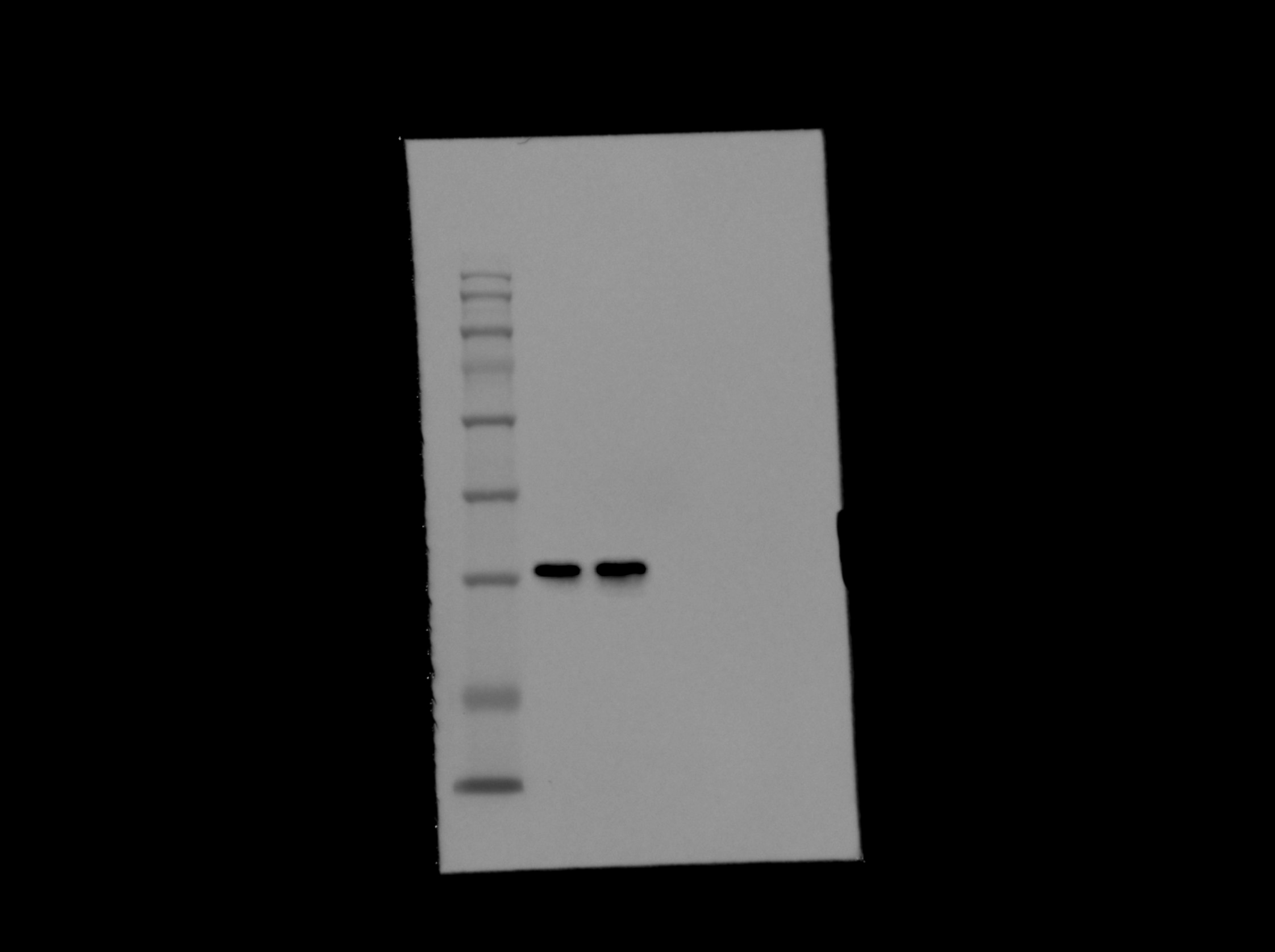
**

**
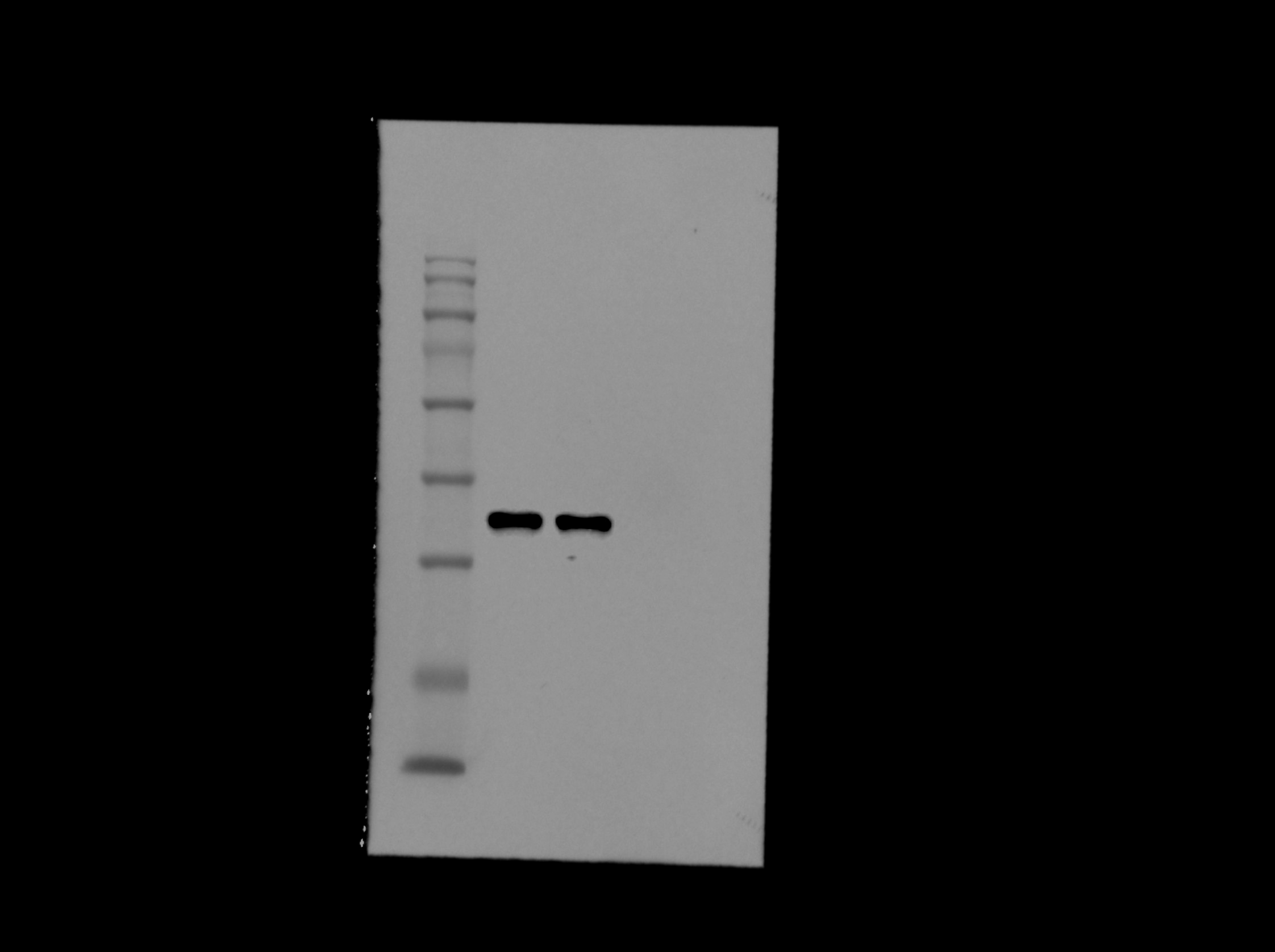
**

**Figure 6I**

**
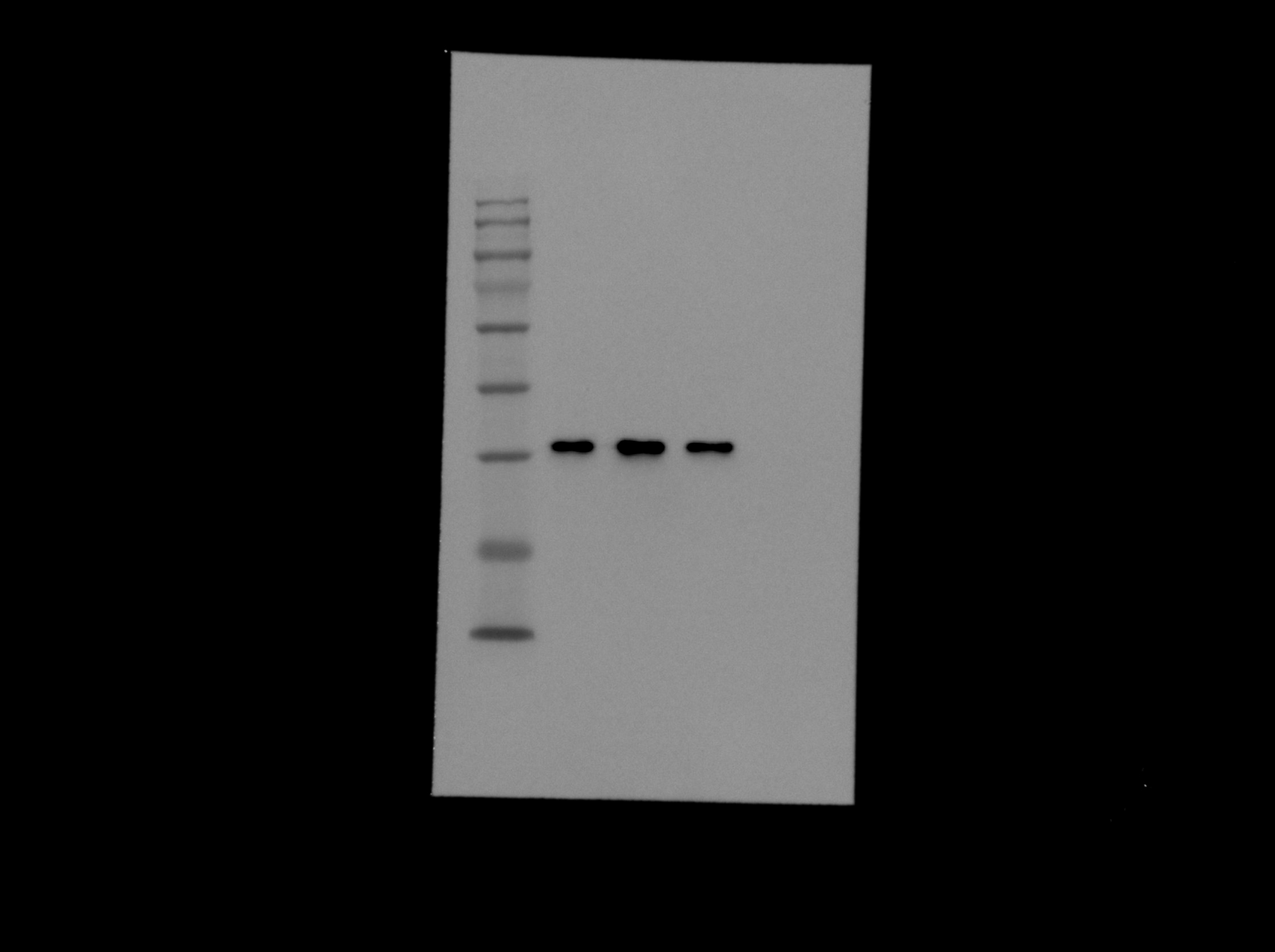
**

**
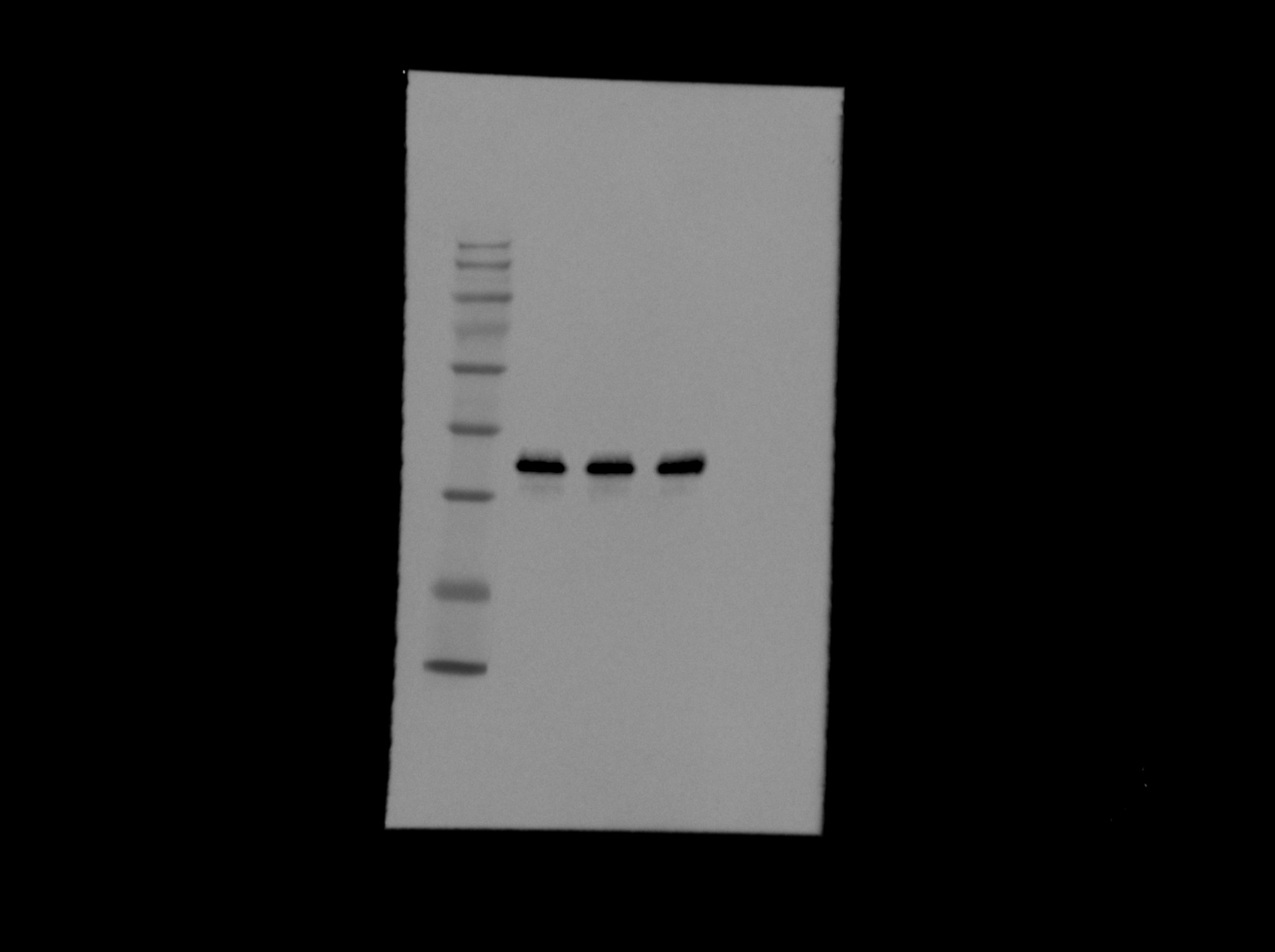
**

**Figure 2B (Supplementary Figure 1A)**

**
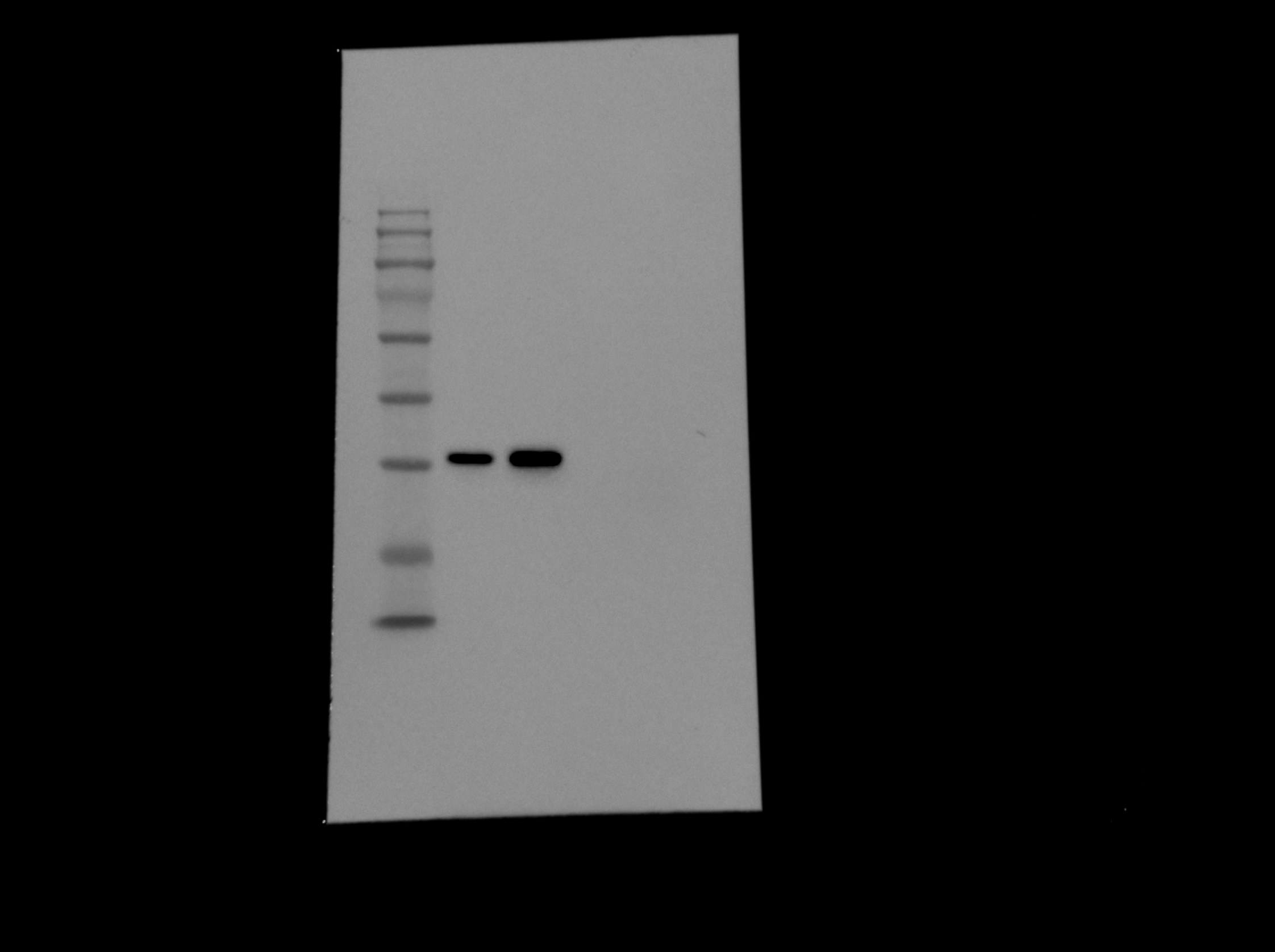
**

**
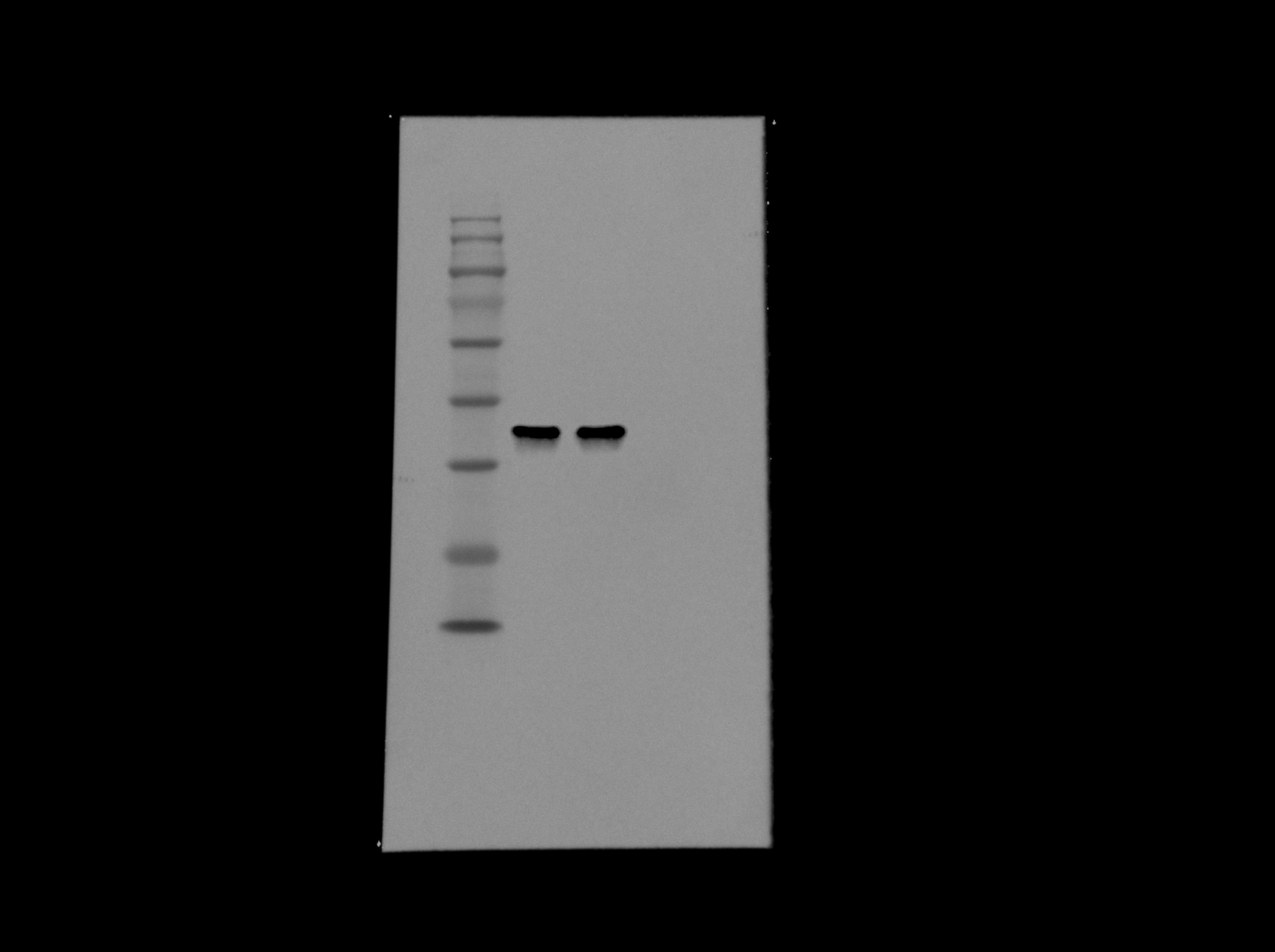
**

**Figure 2D (Supplementary Figure 1B)**

**
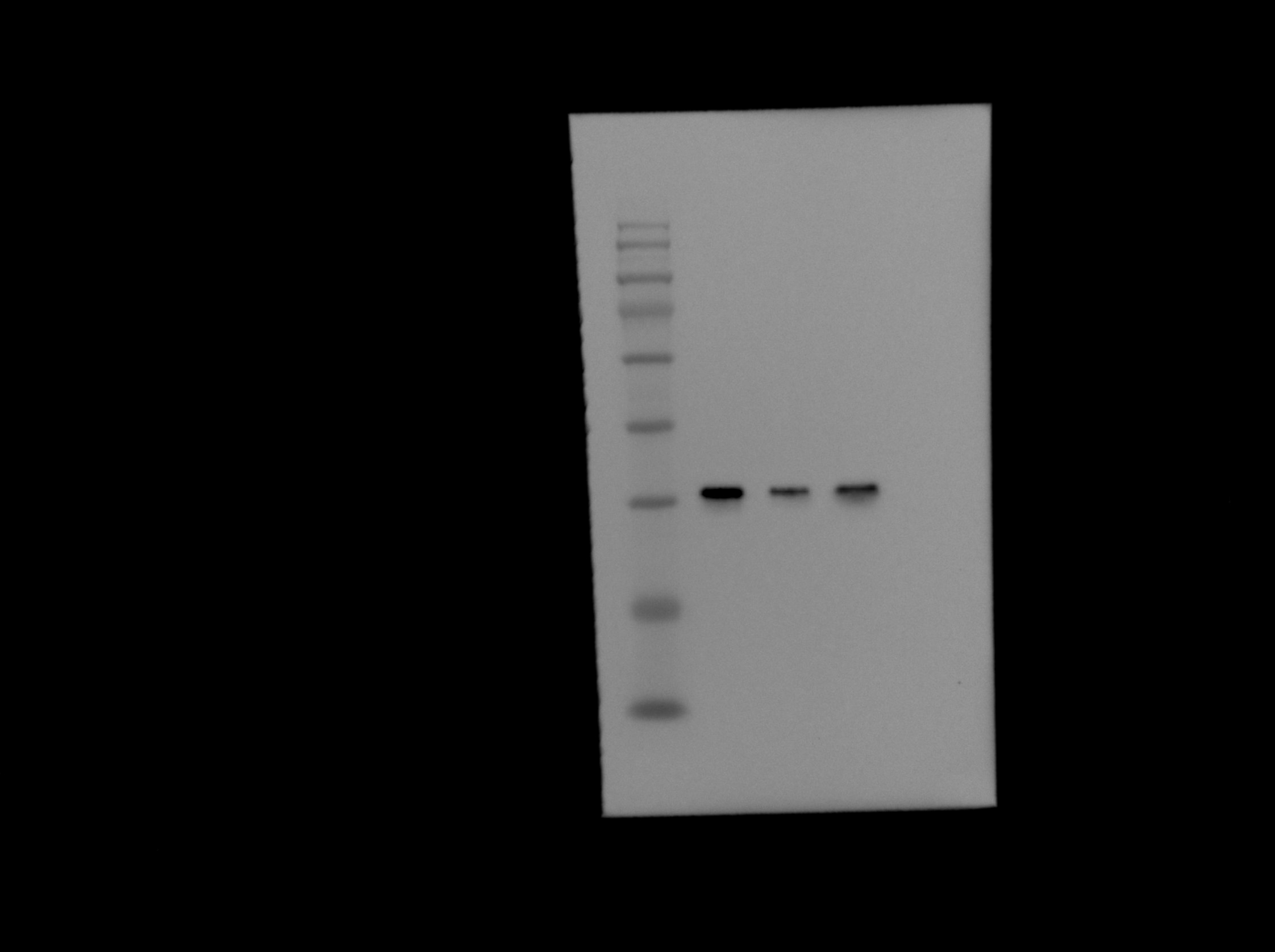
**

**
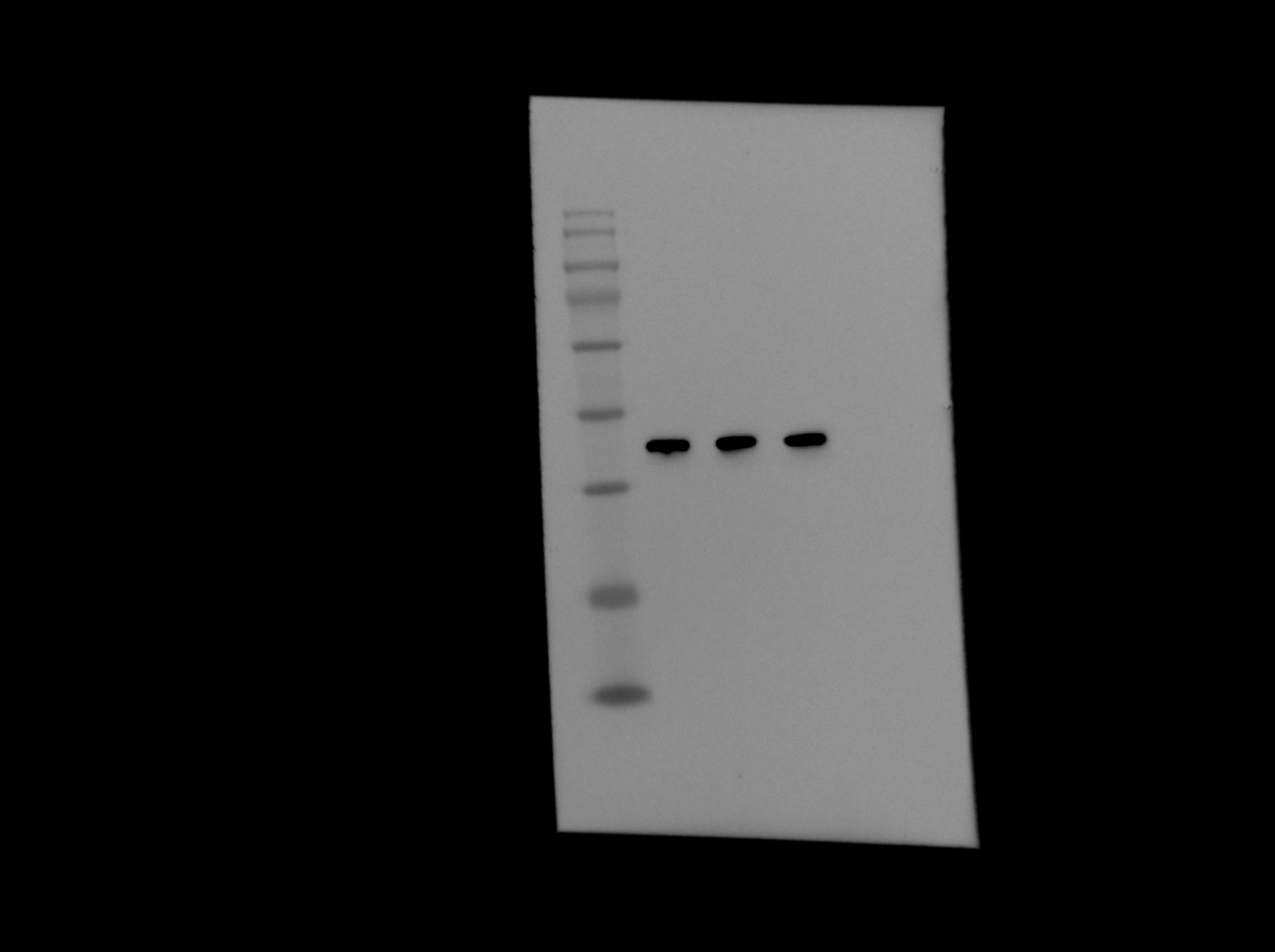
**

**Figure 2H (Supplementary Figure 1E)**

**
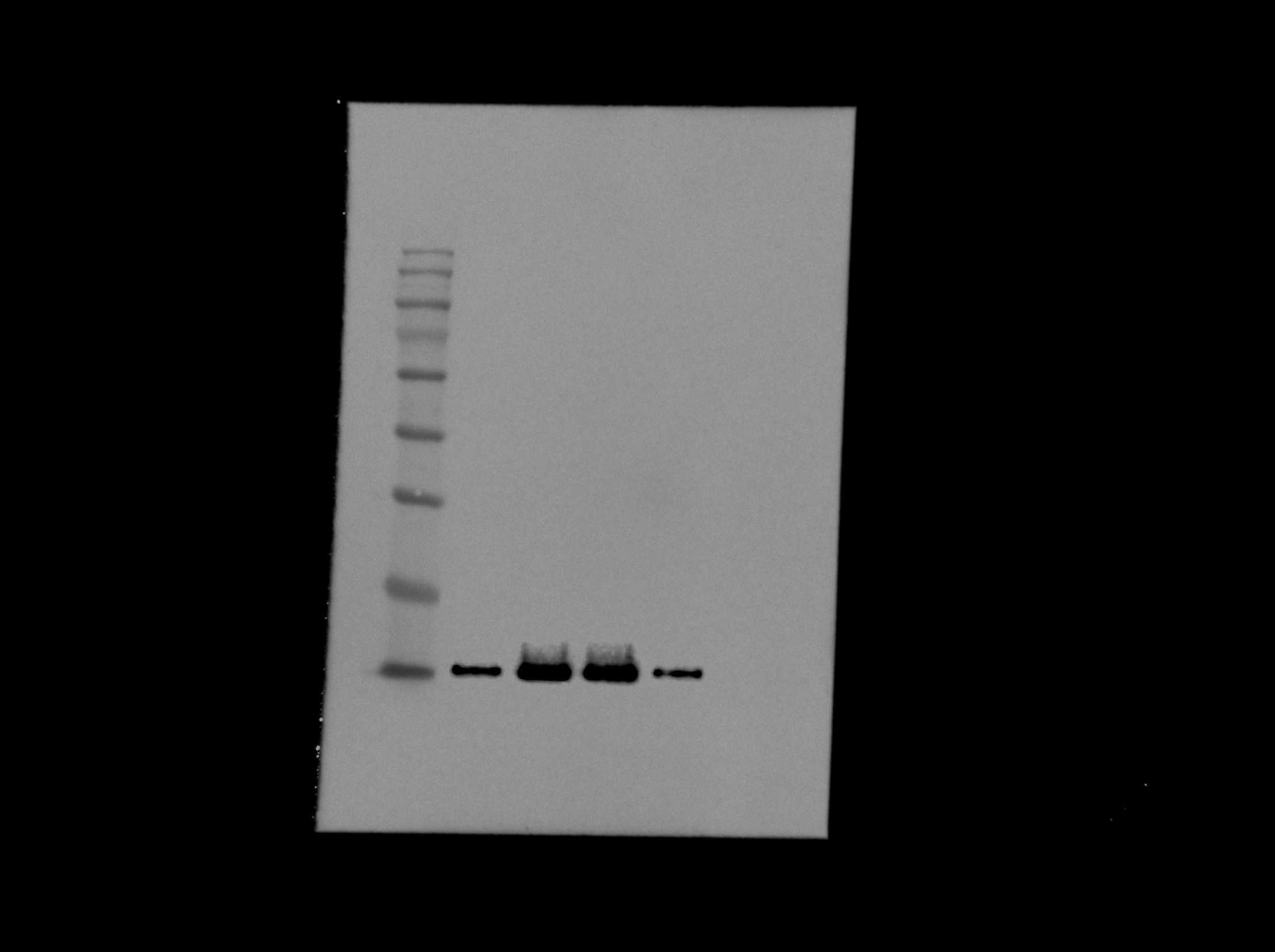
**

**
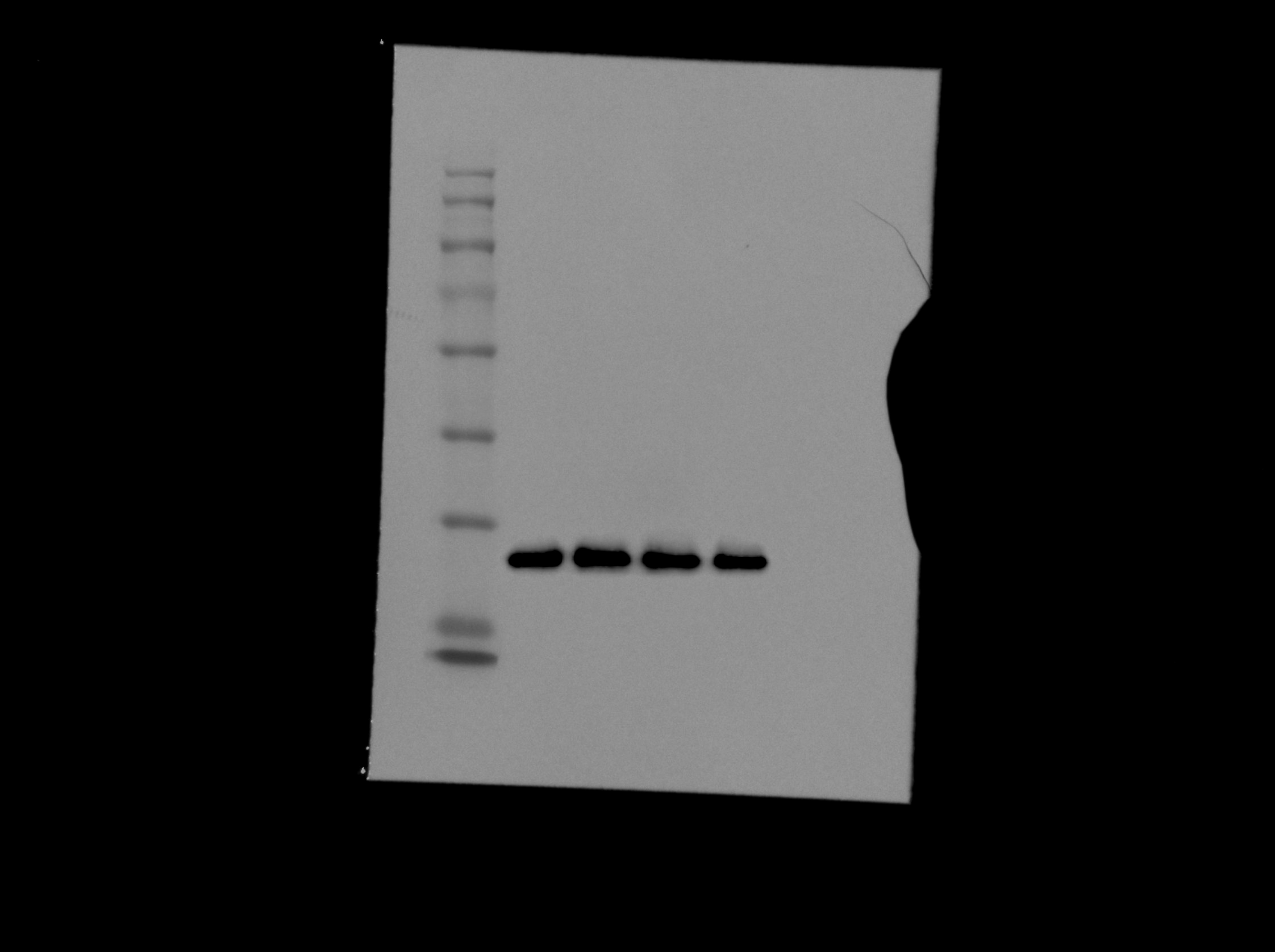
**

**
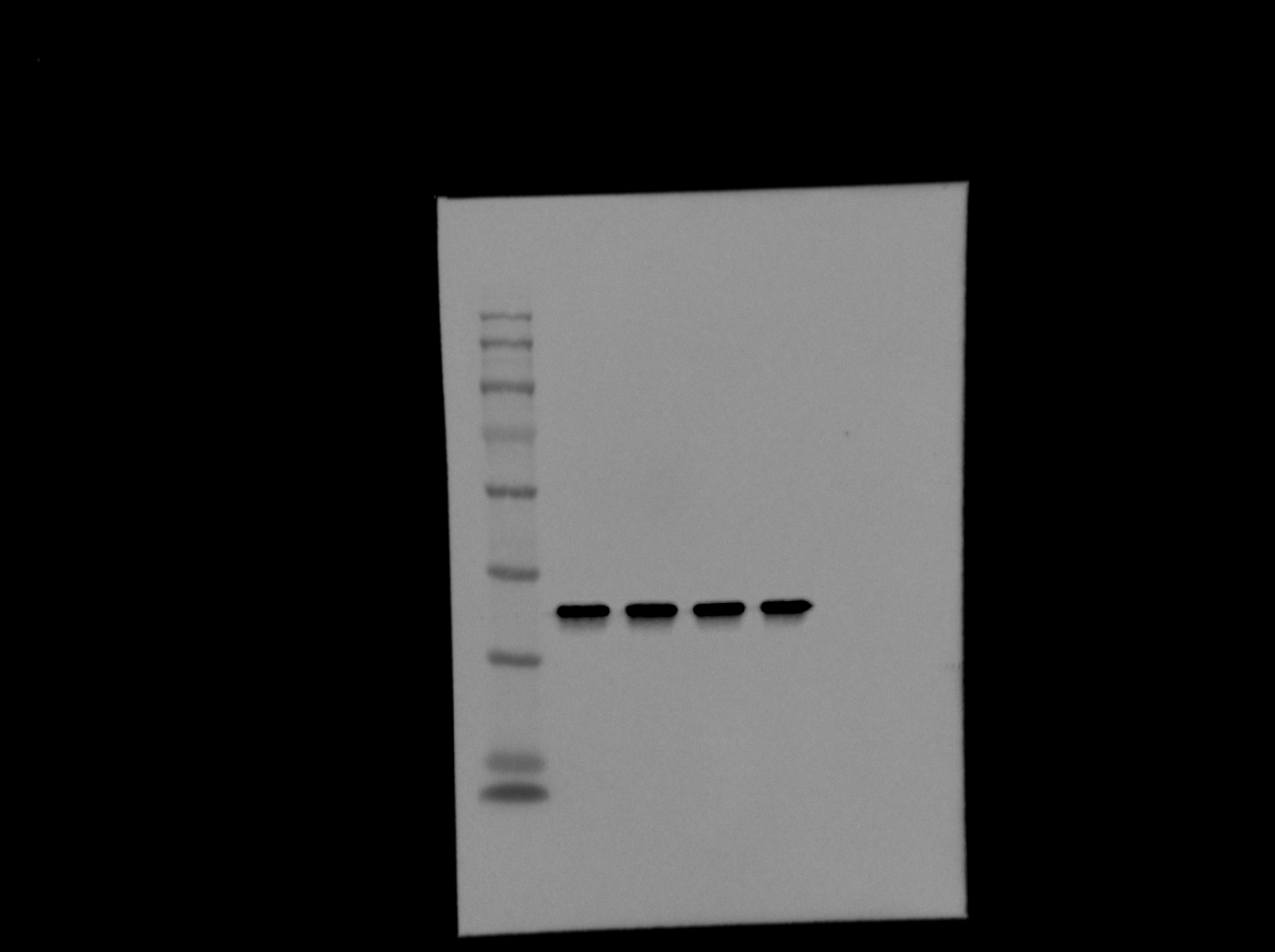
**

**Figure 5B (Supplementary Figure 2A)**

**
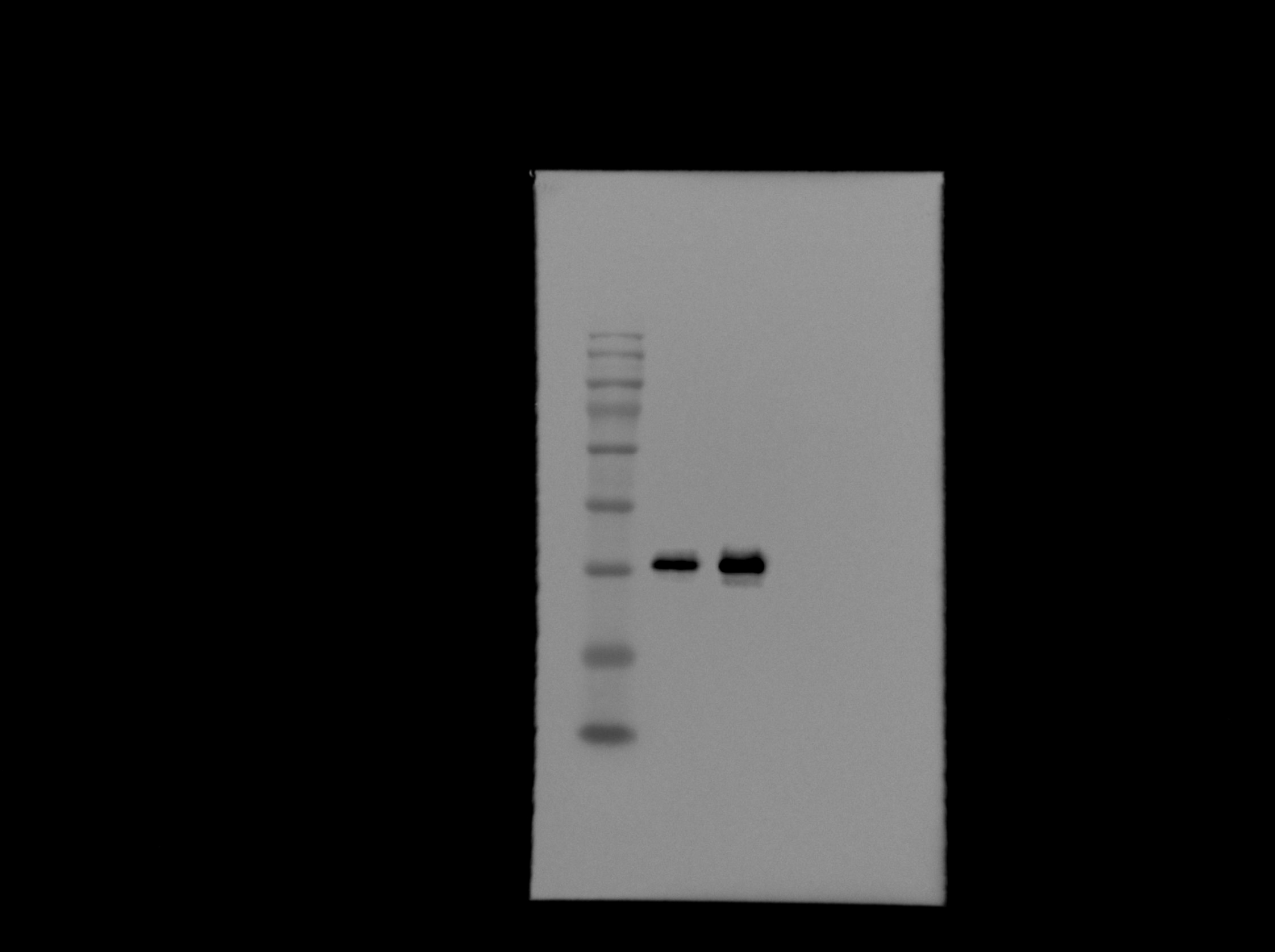
**

**
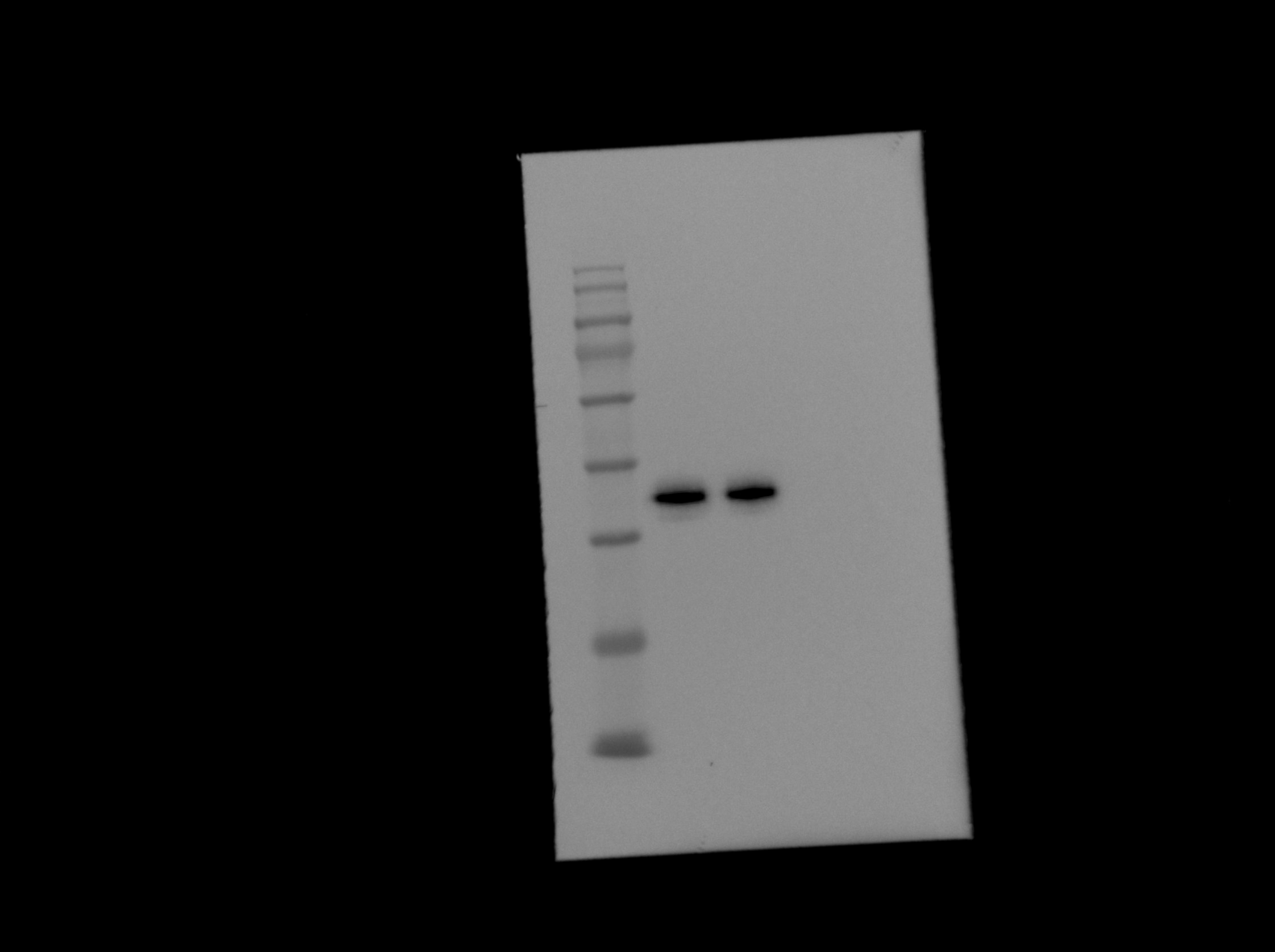
**

**Figure 5D (Supplementary Figure 2B)**

**
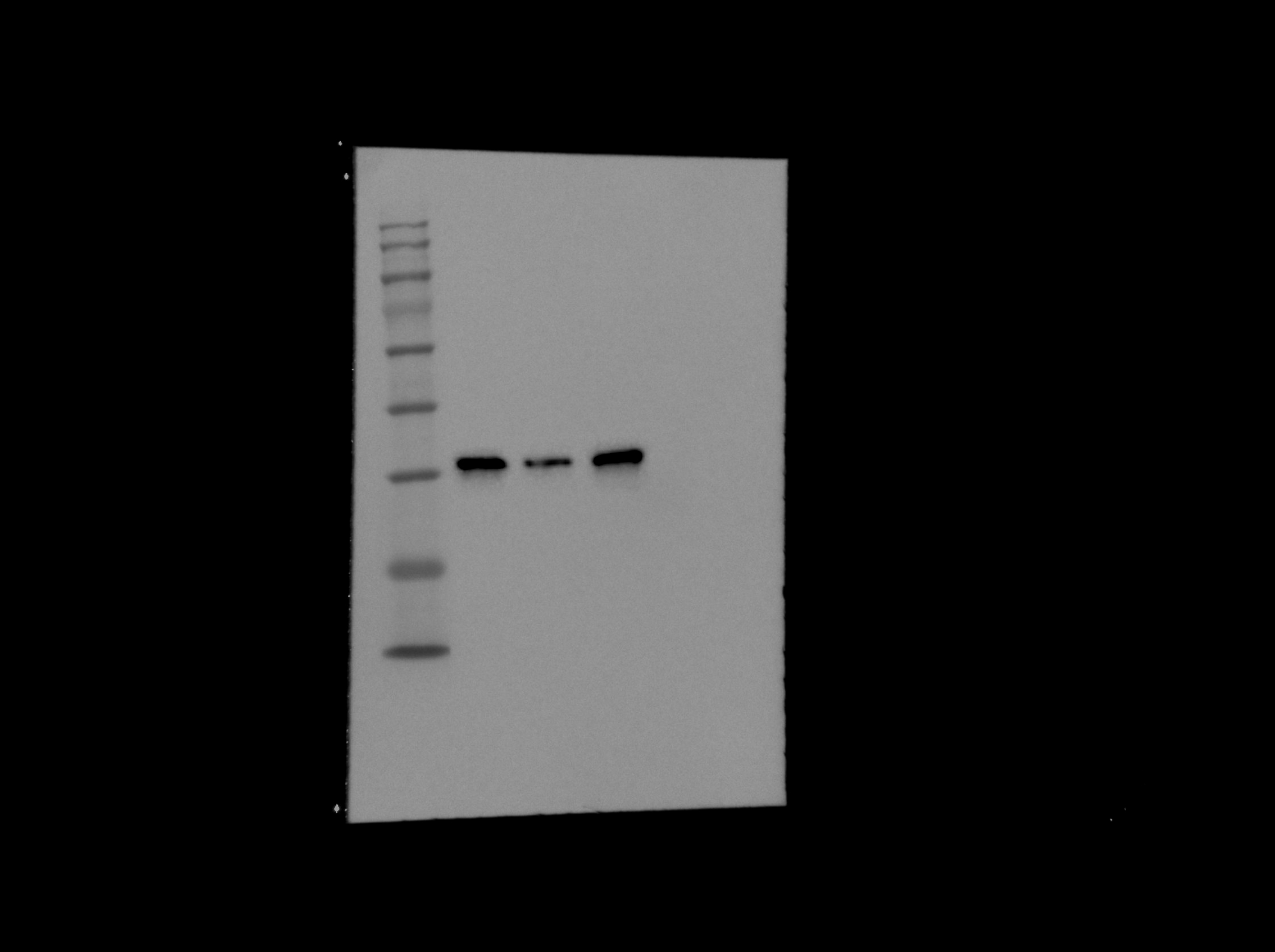
**

**
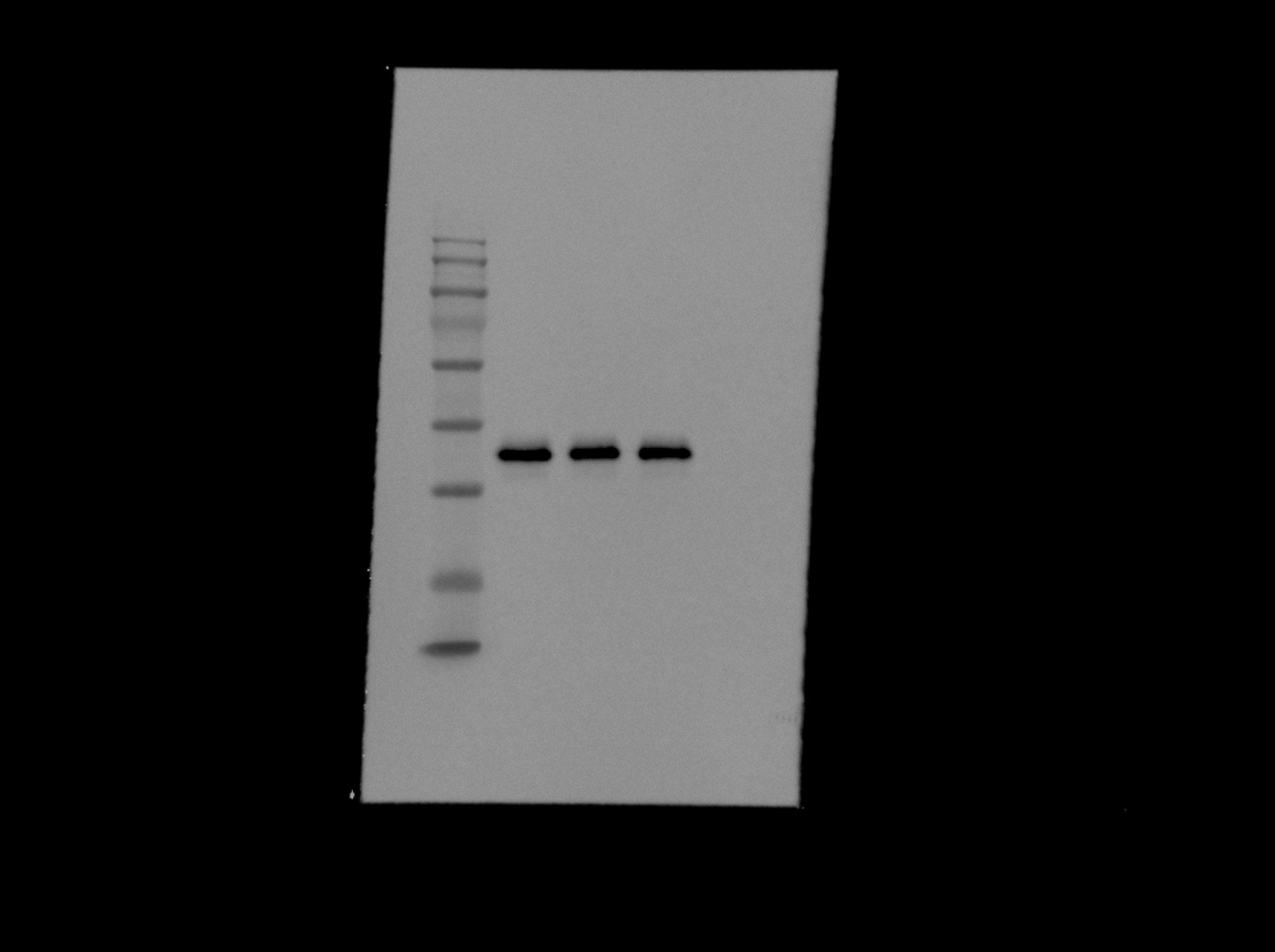
**

**Figure 5H (Supplementary Figure 2E)**

**
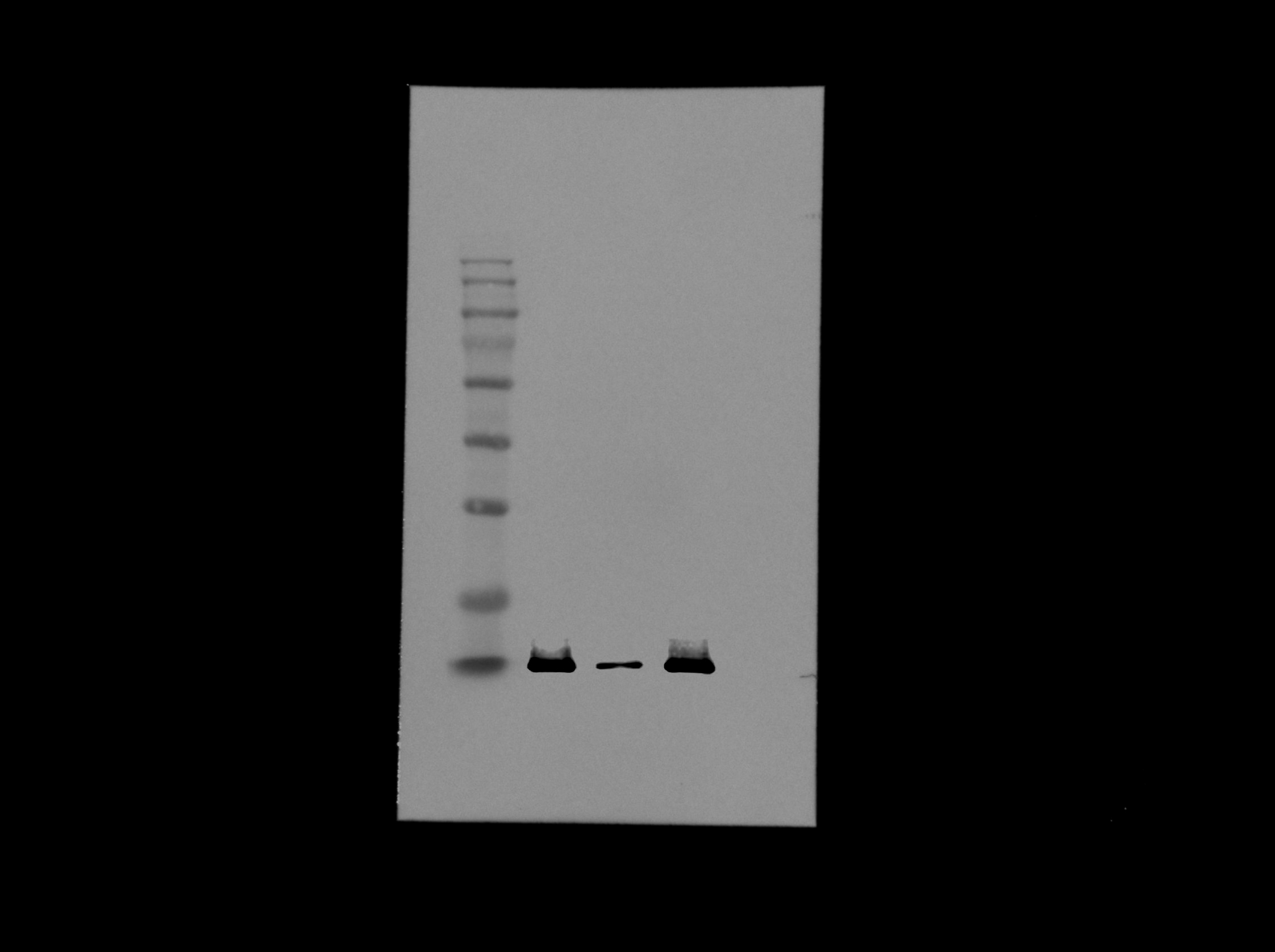
**

**
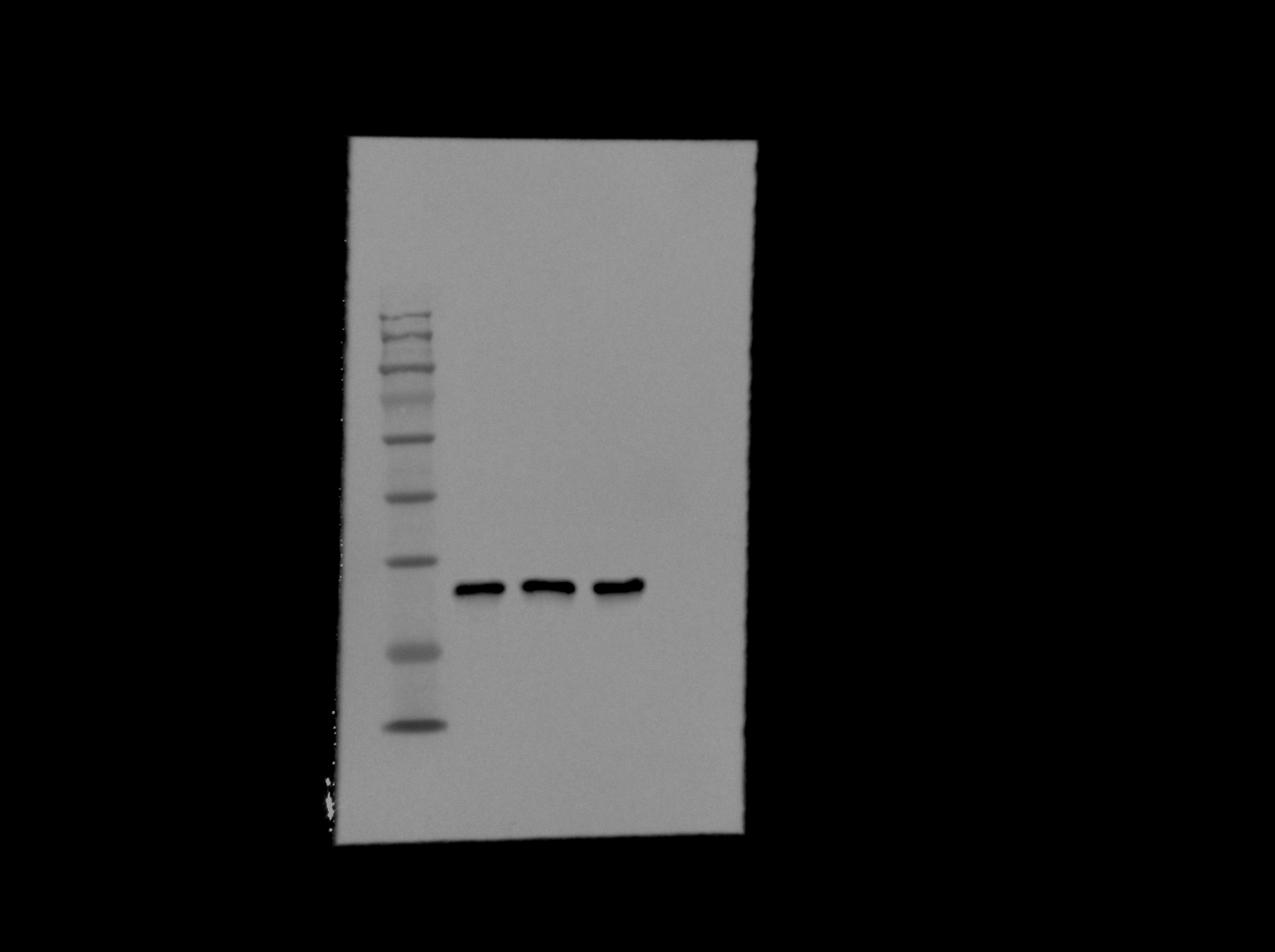
**

**
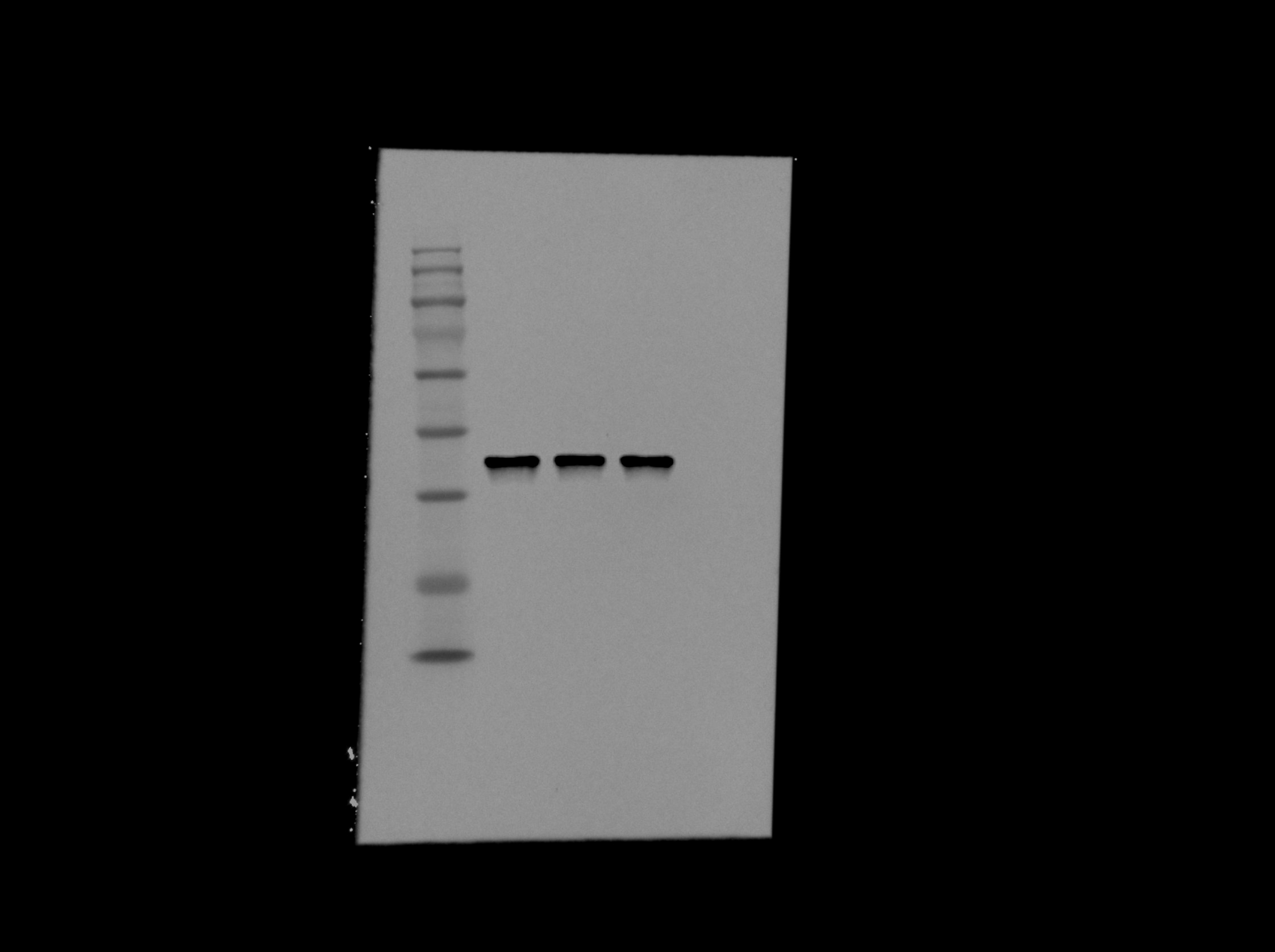
**

**Figure 7A (Supplementary Figure 3A)**

**
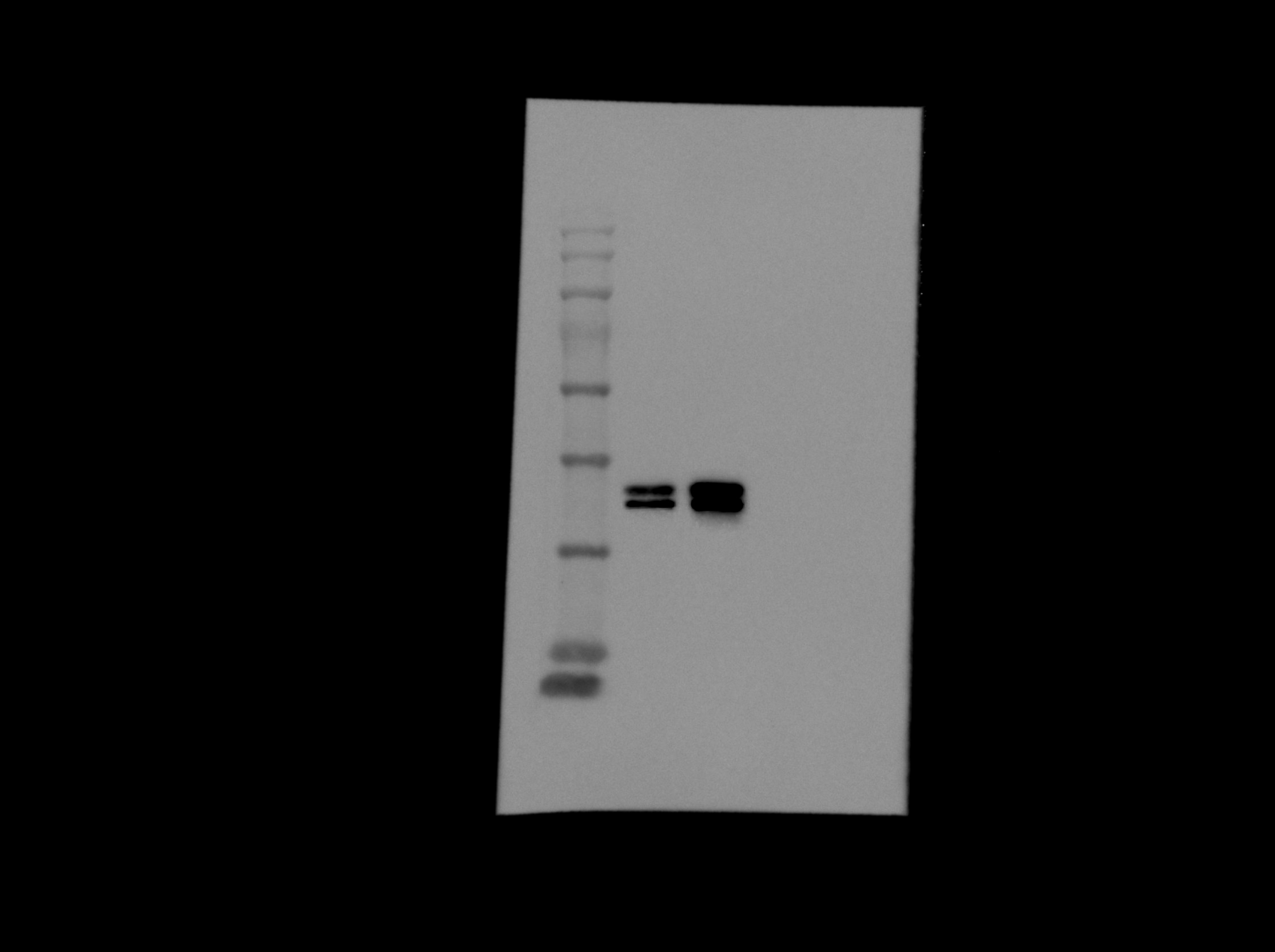
**

**
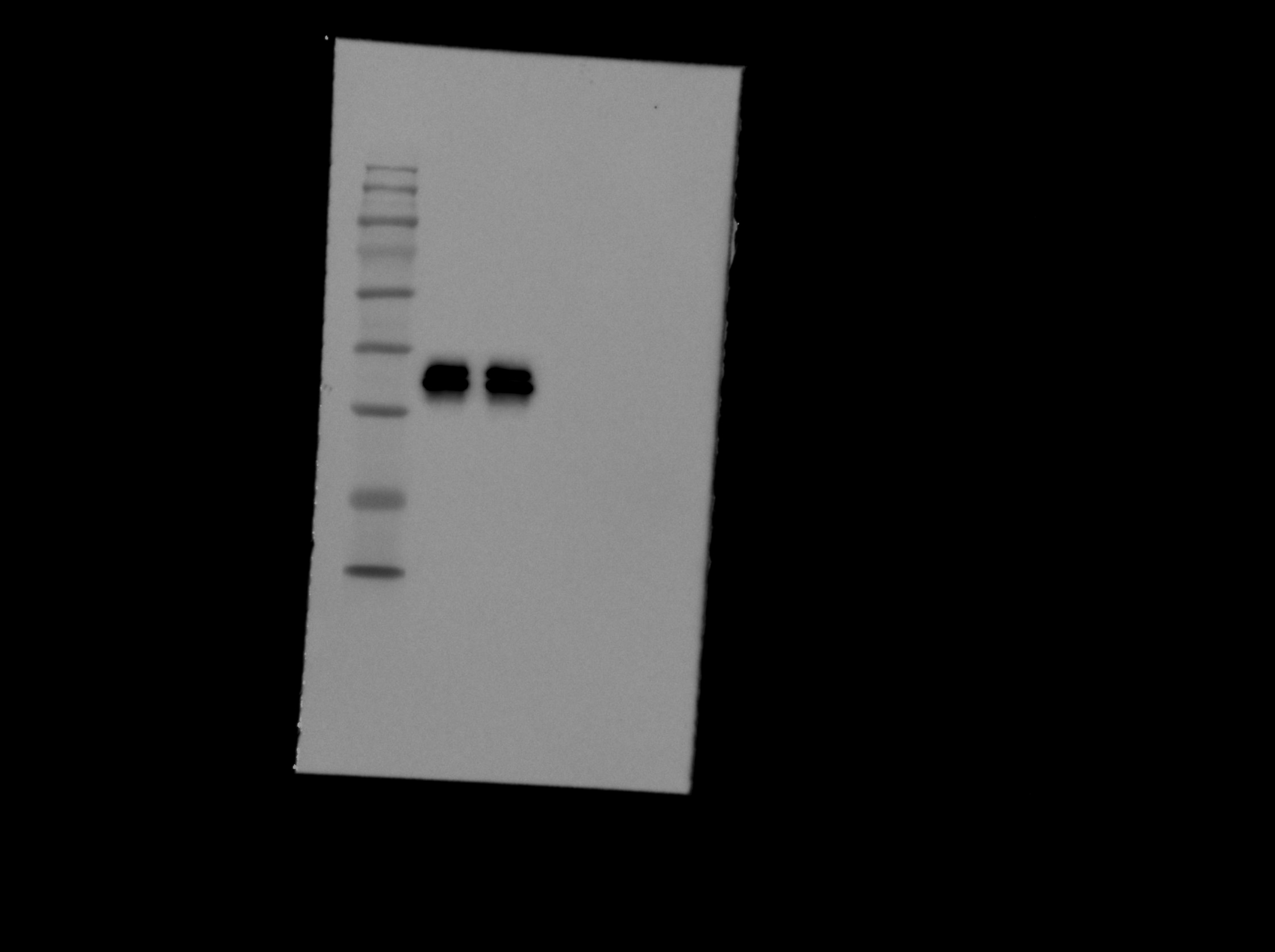
**

**
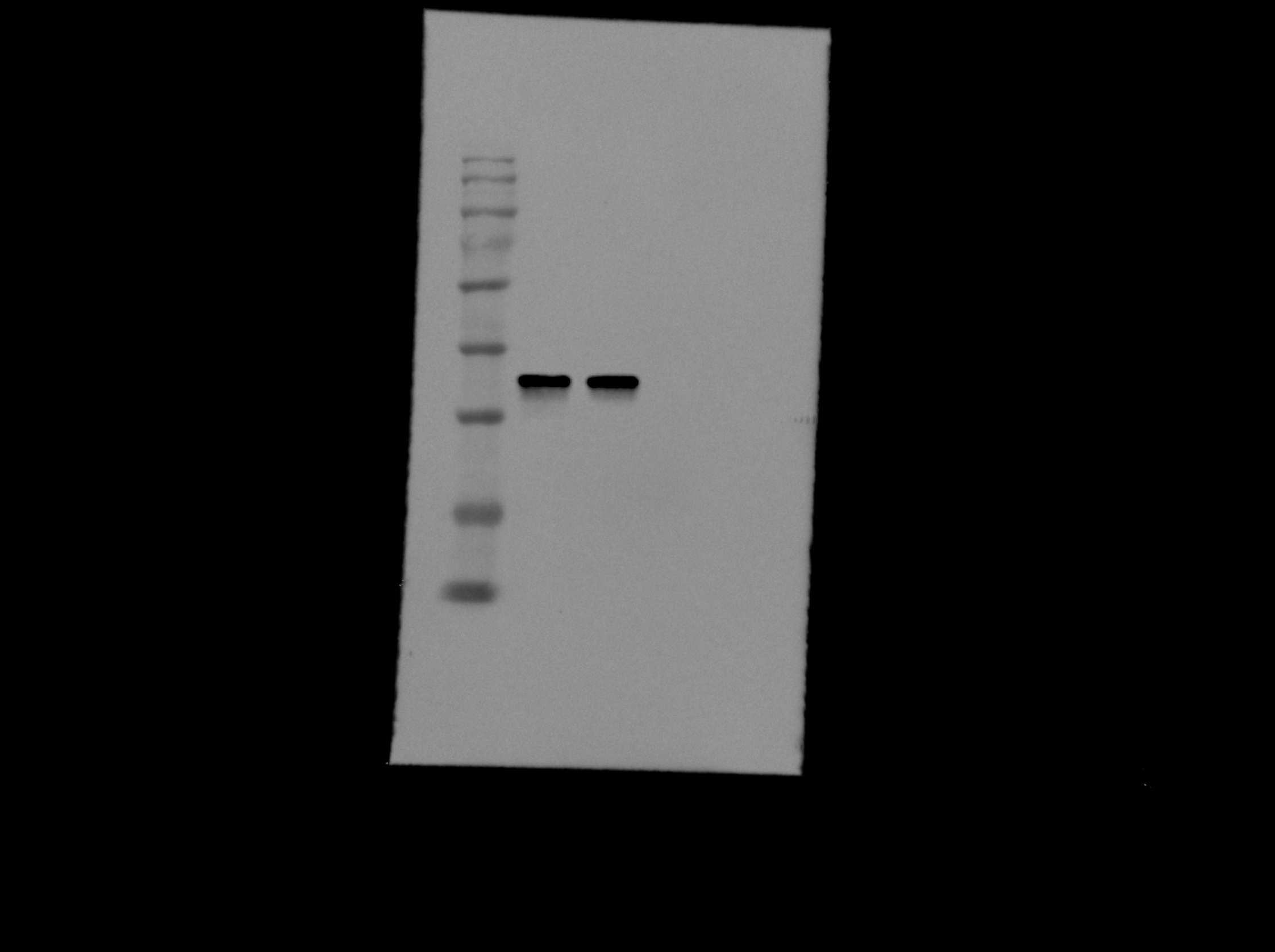
**

**Figure 7D (Supplementary Figure 3B)**

**
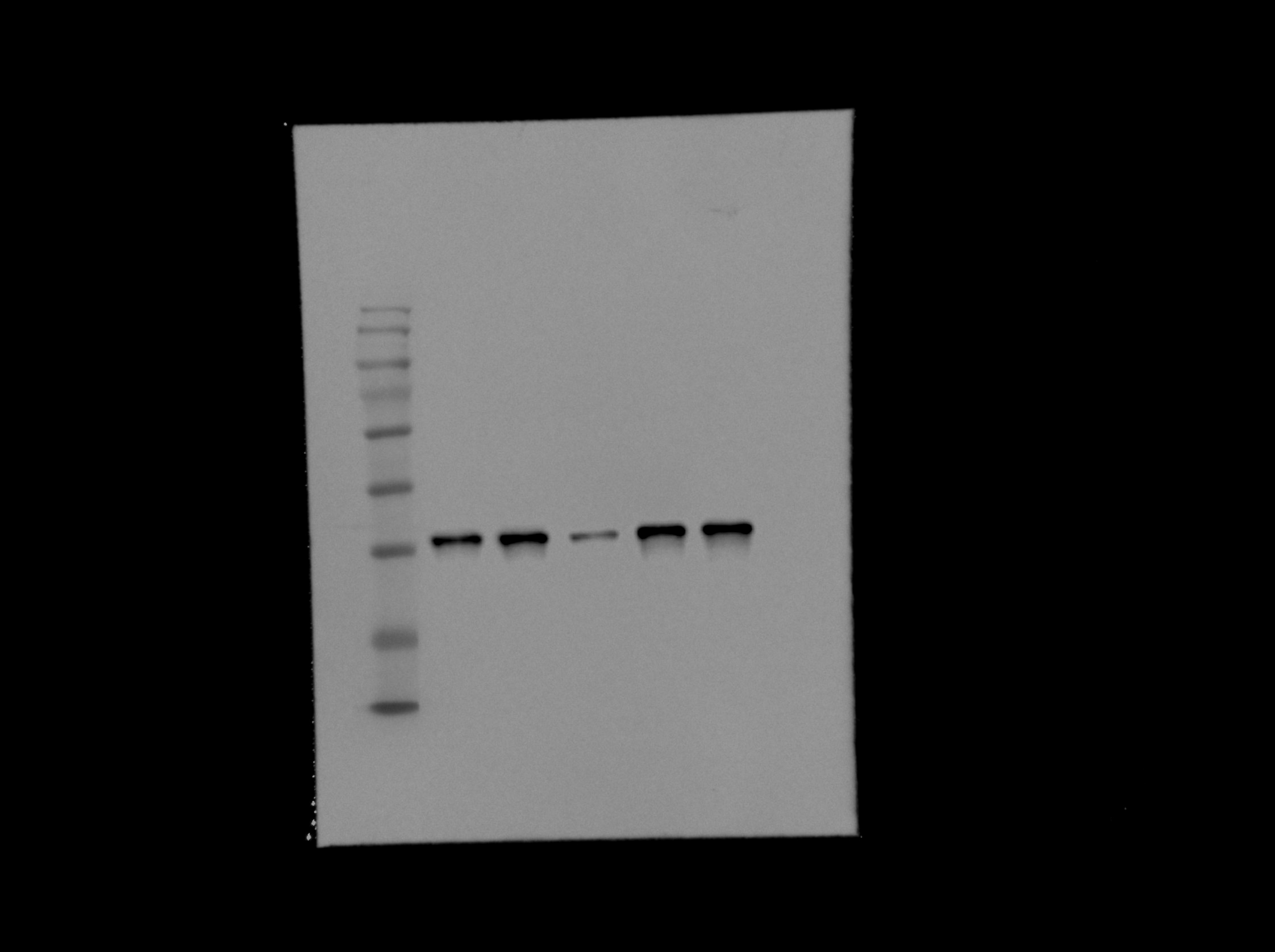
**

**
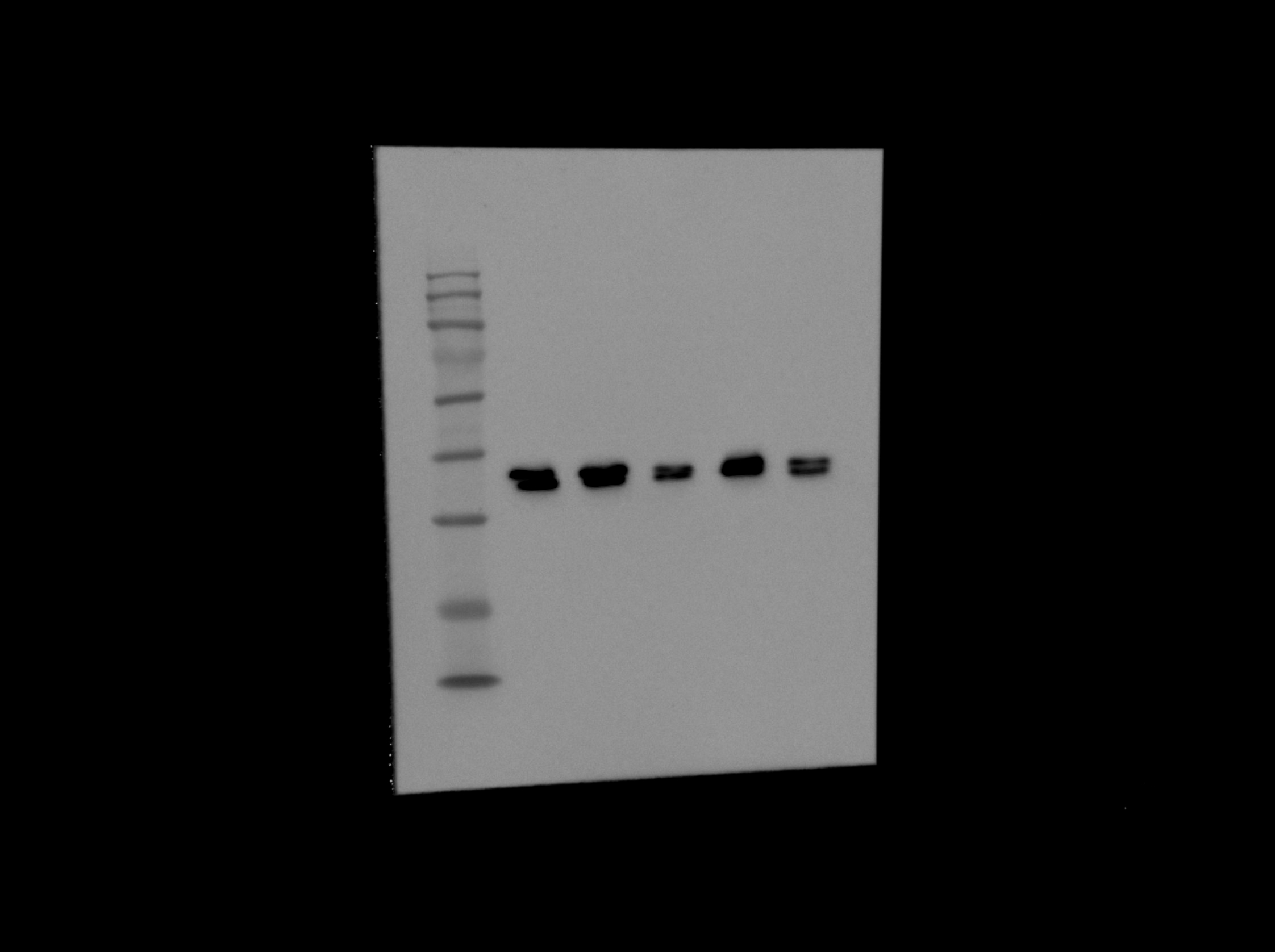
**

**
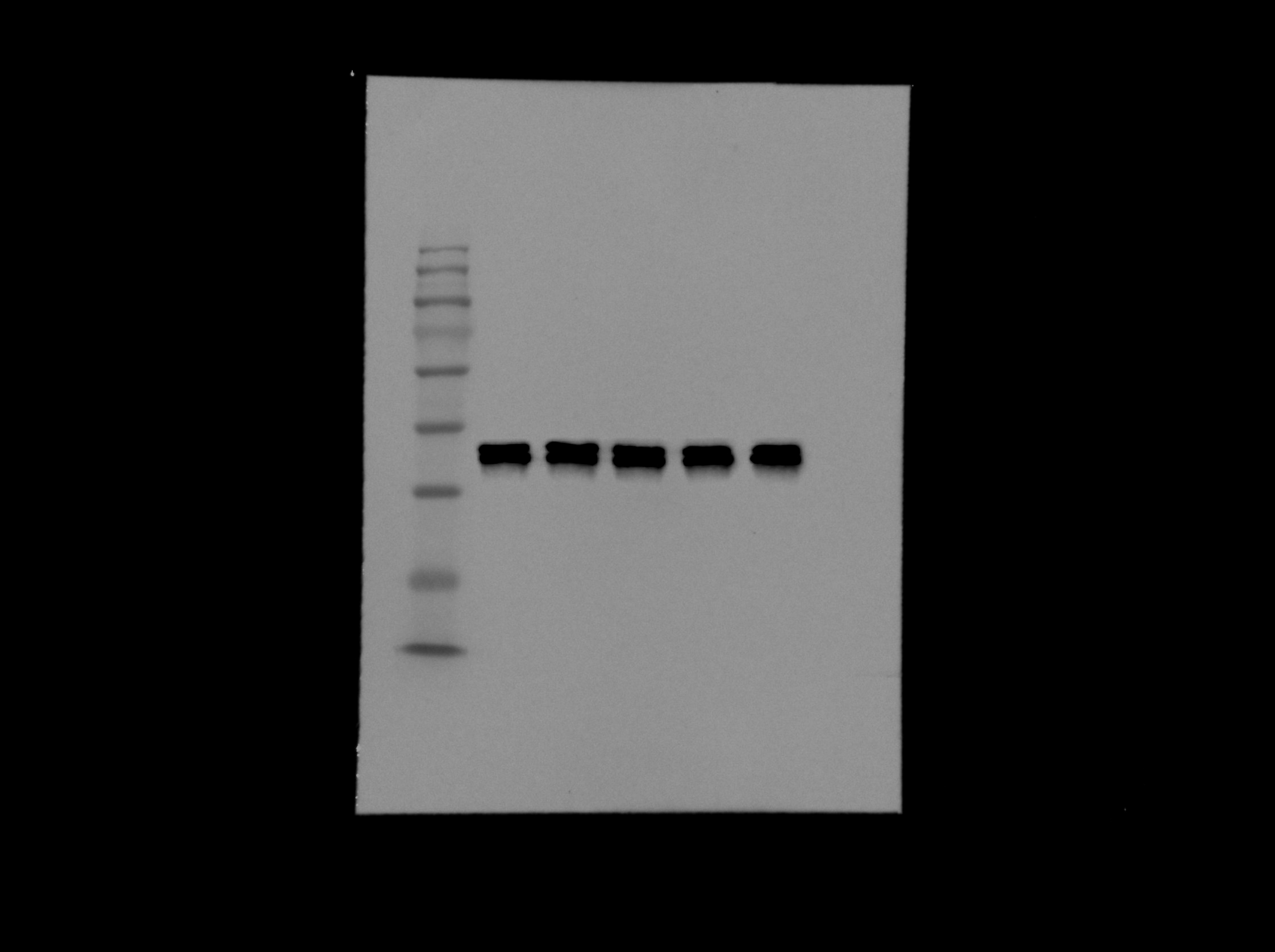
**

**
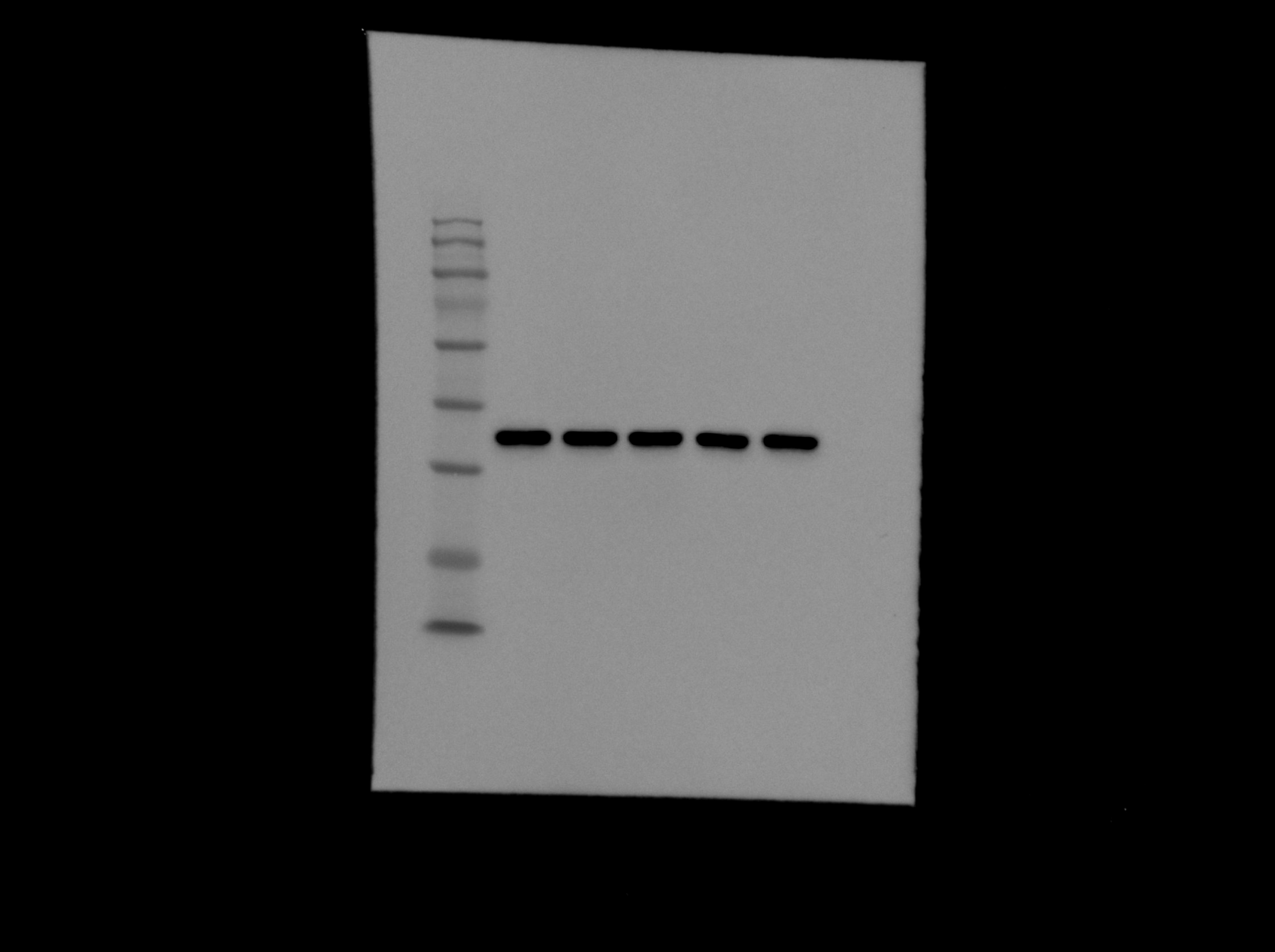
**

**Figure 7H (Supplementary Figure 3E)**

**
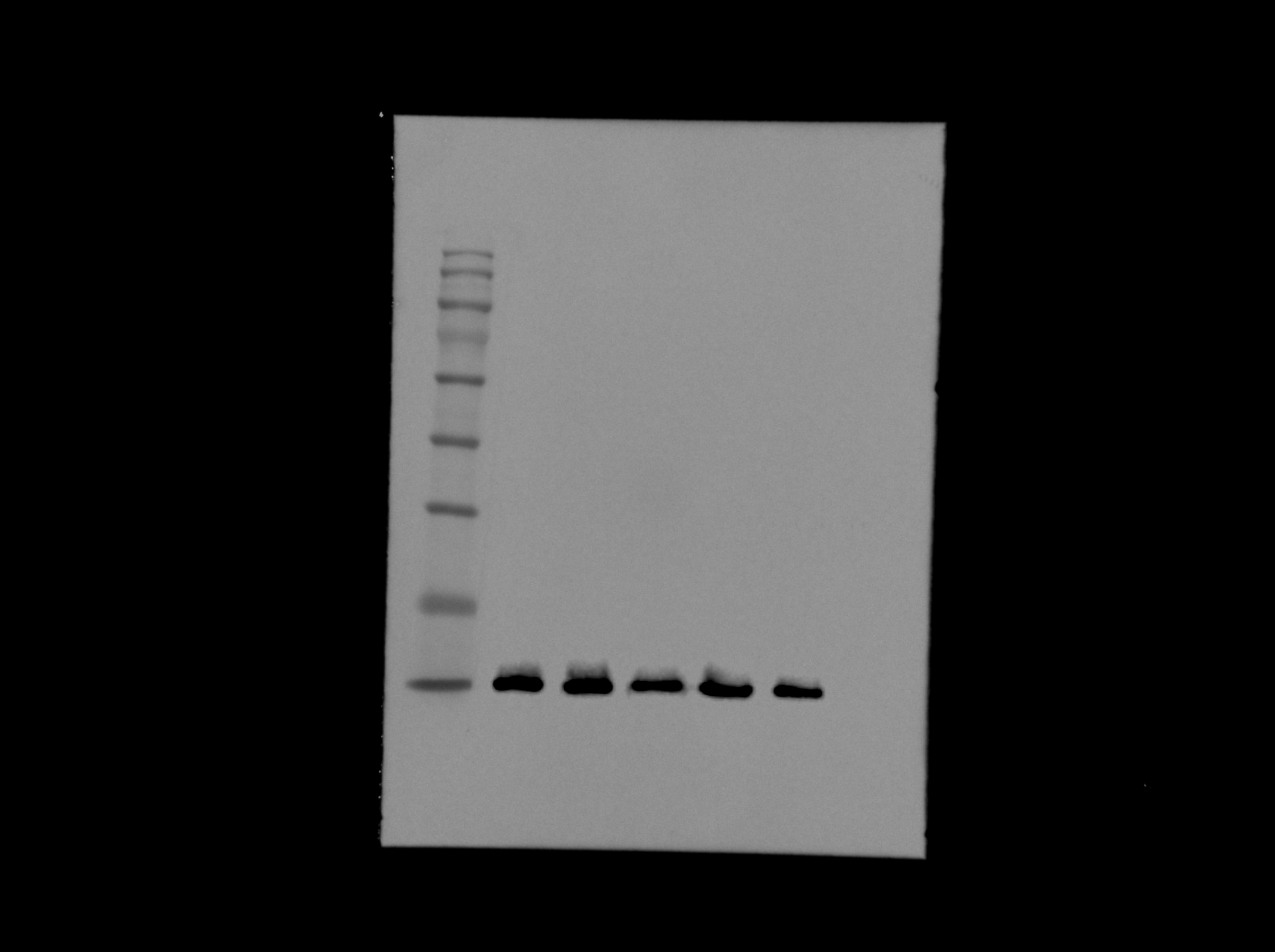
**

**
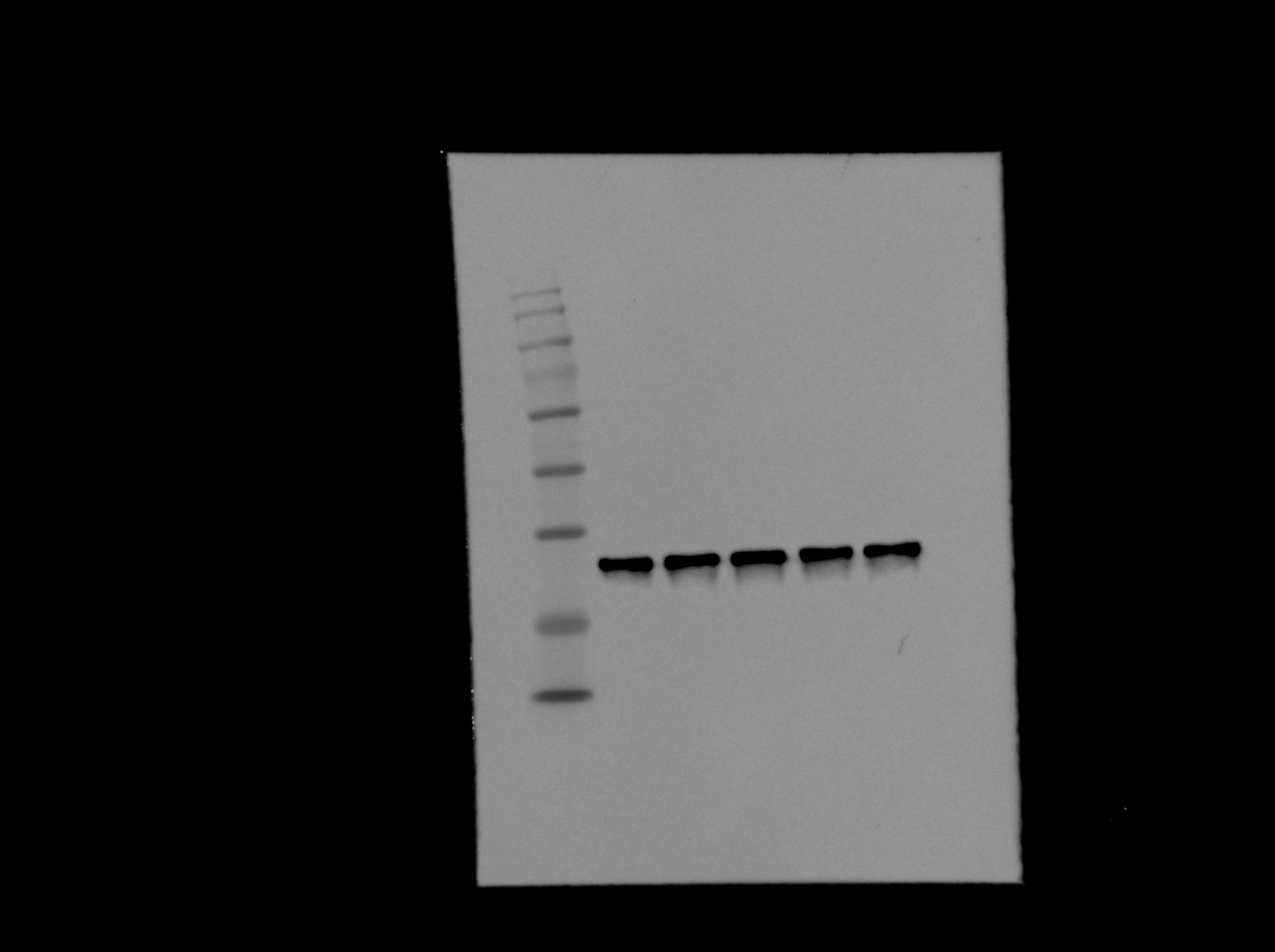
**

**
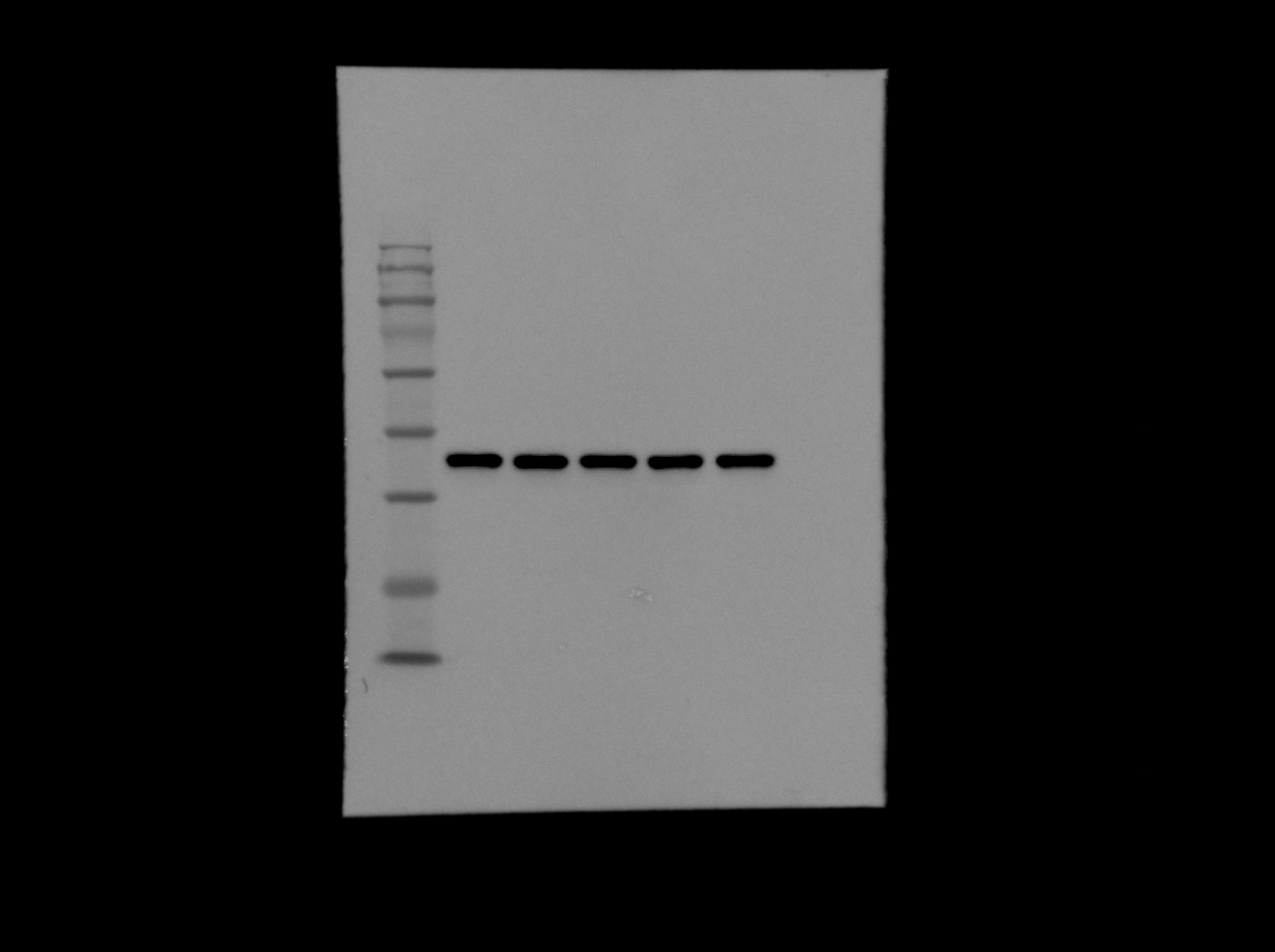
**

**Figure 8A (Supplementary Figure 4A)**

**
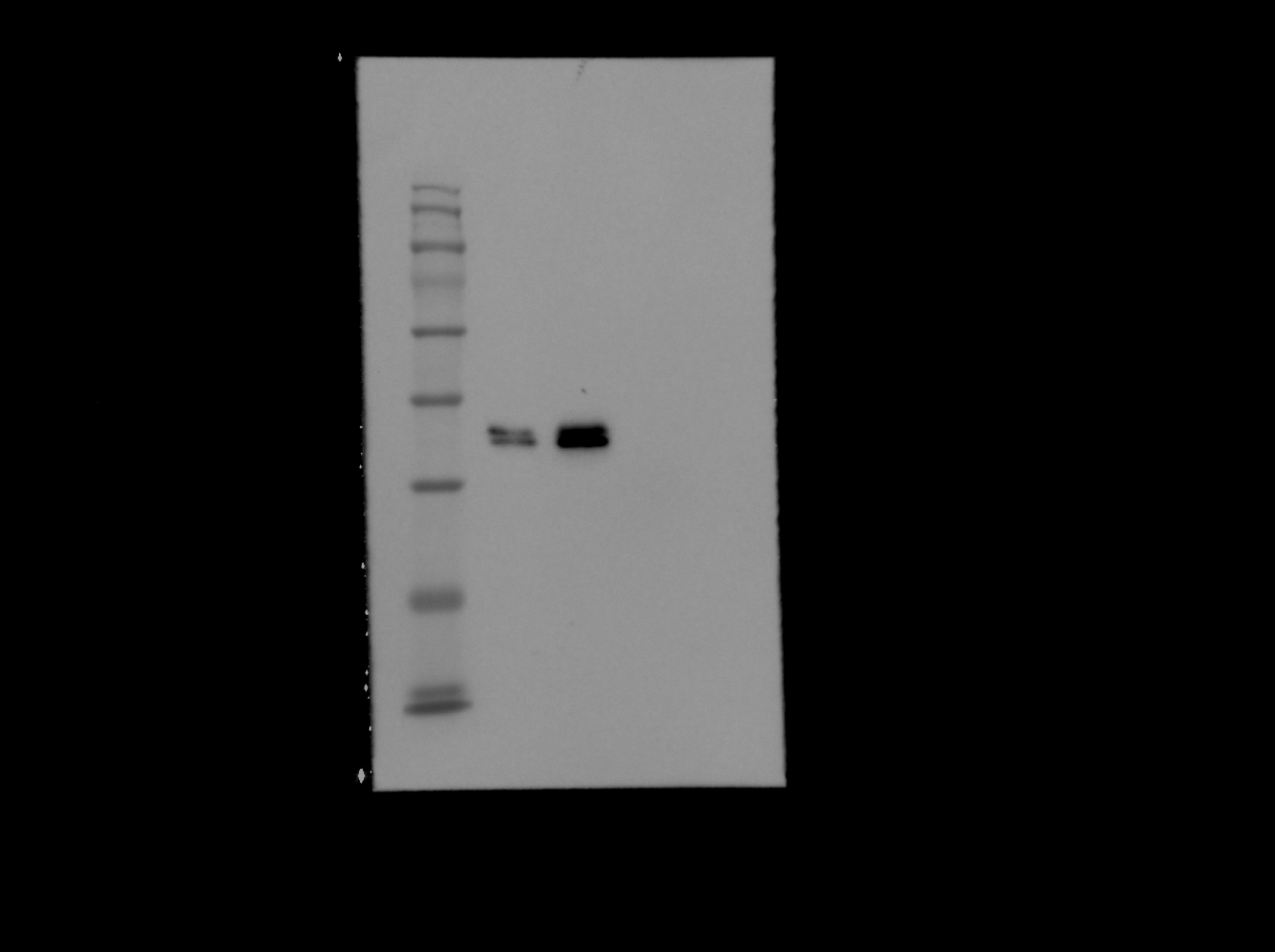
**

**
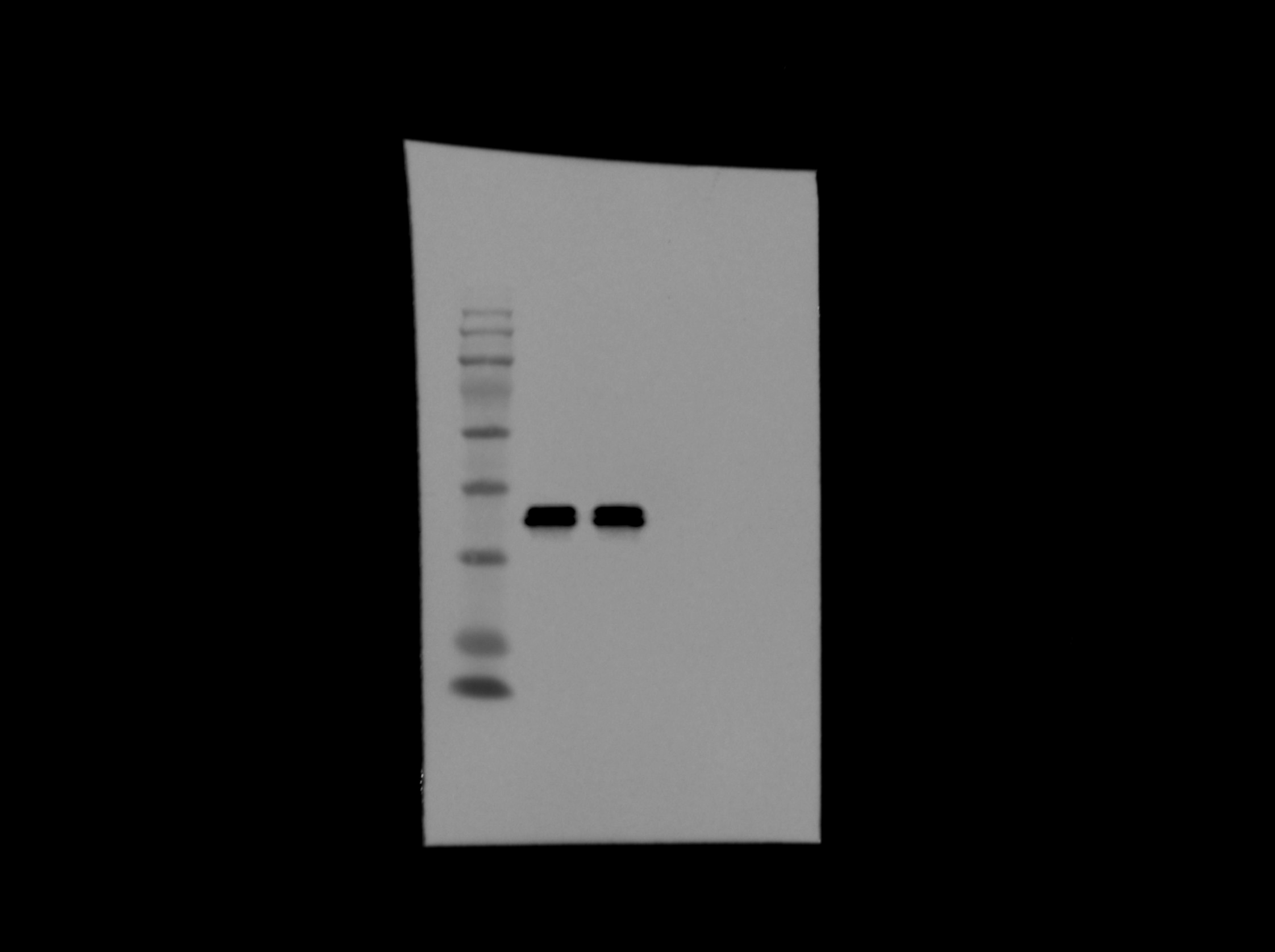
**

**
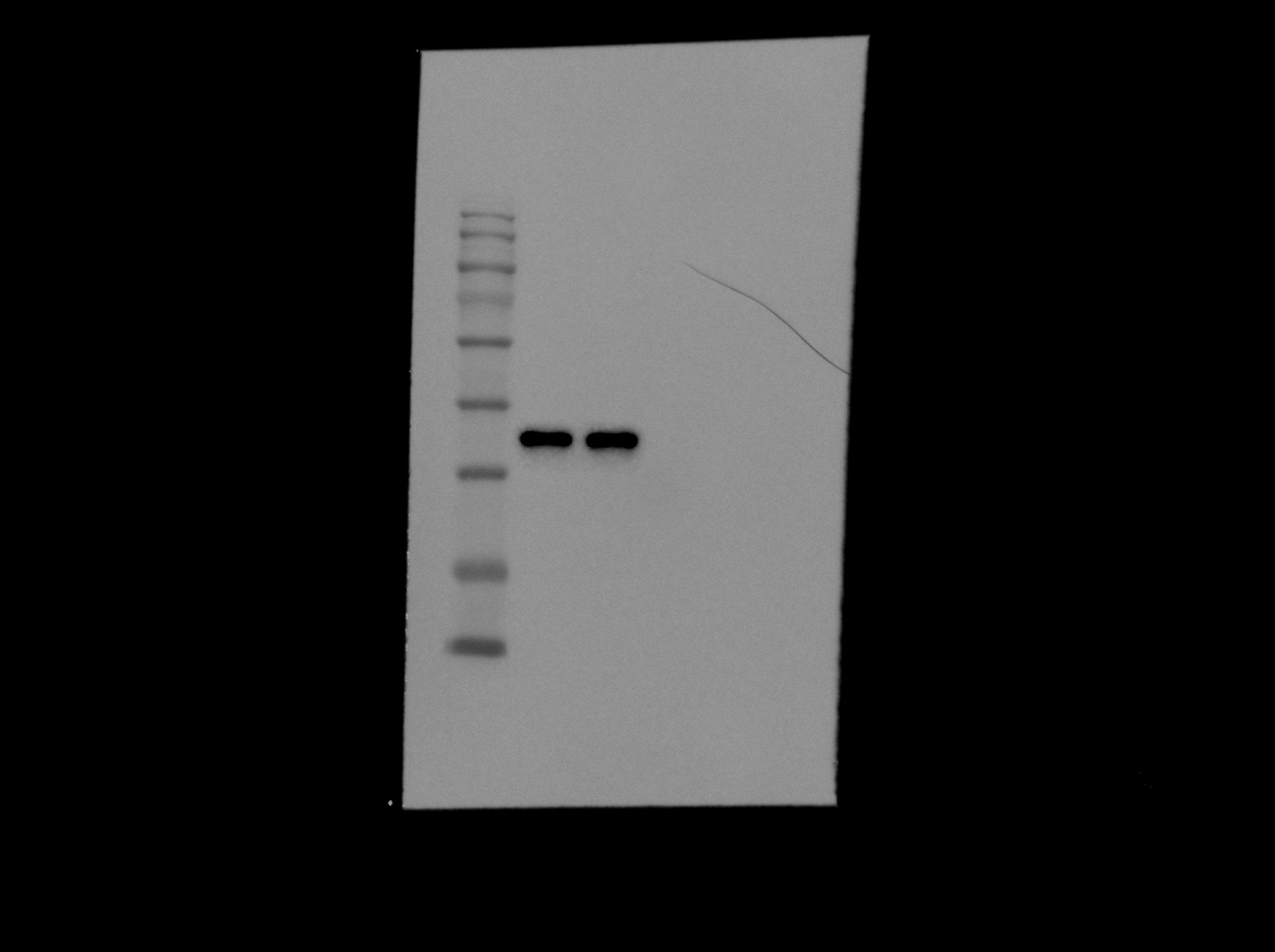
**

**Figure 8C (Supplementary Figure 4B)**

**
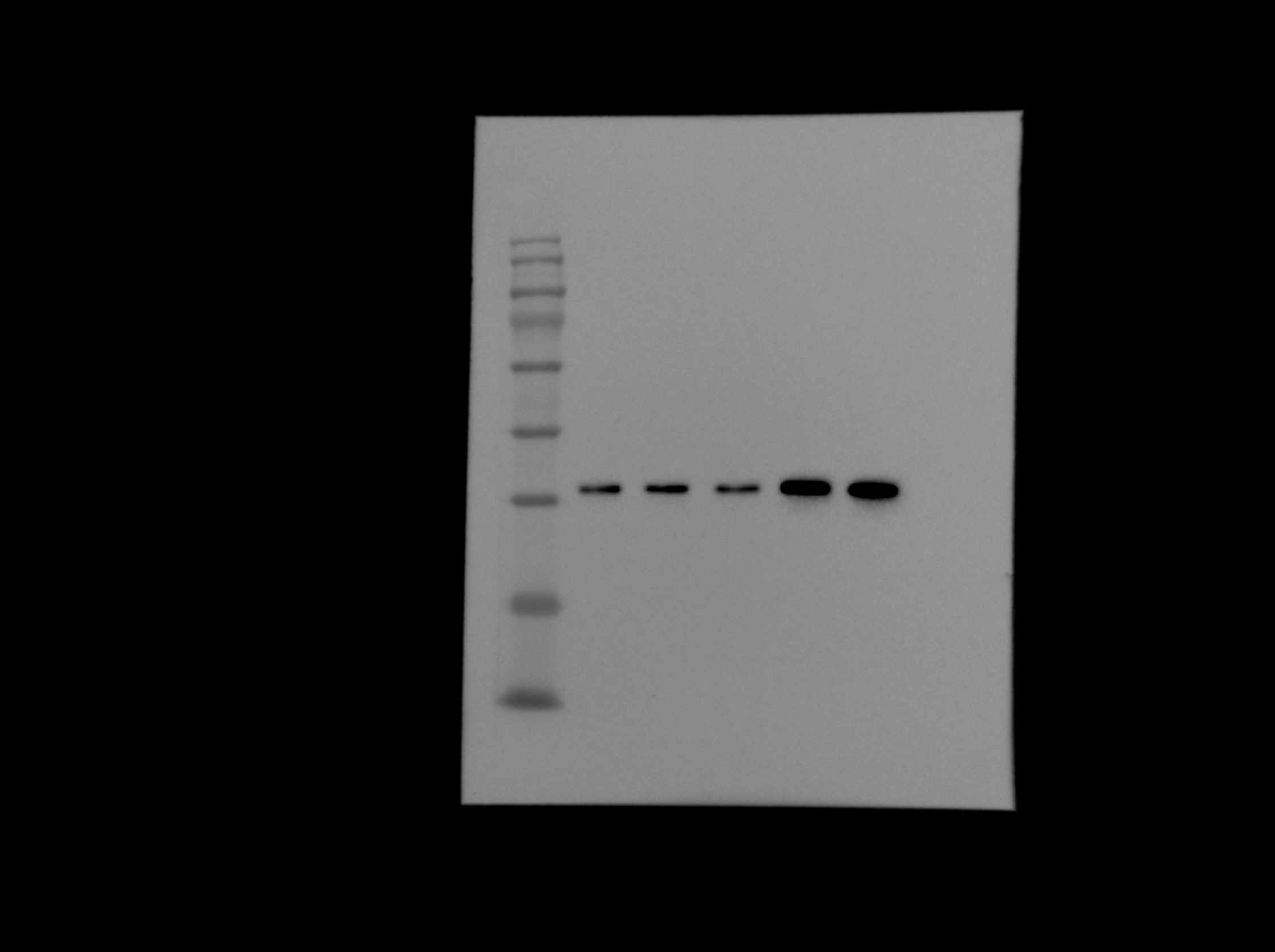
**

**
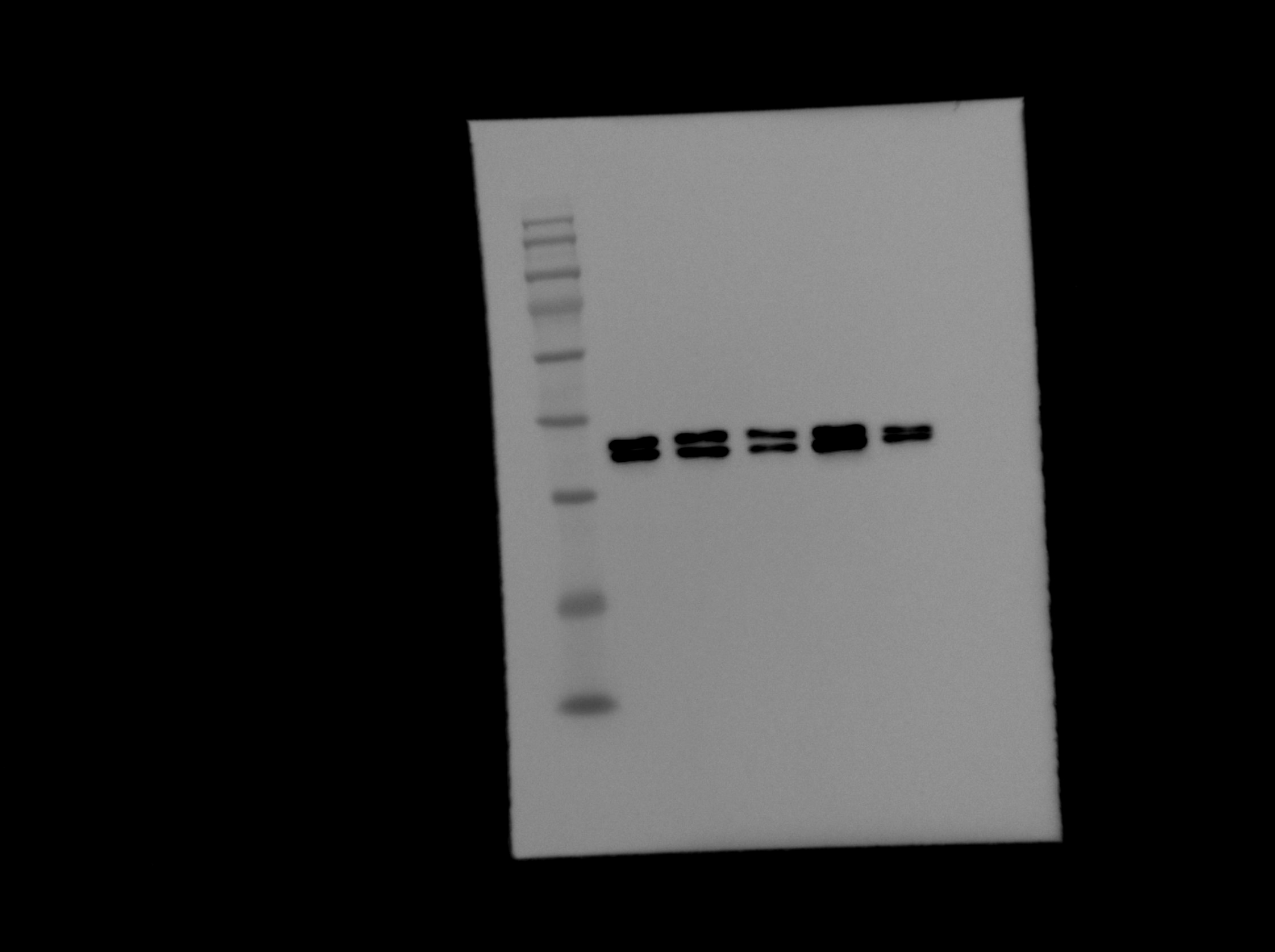
**

**
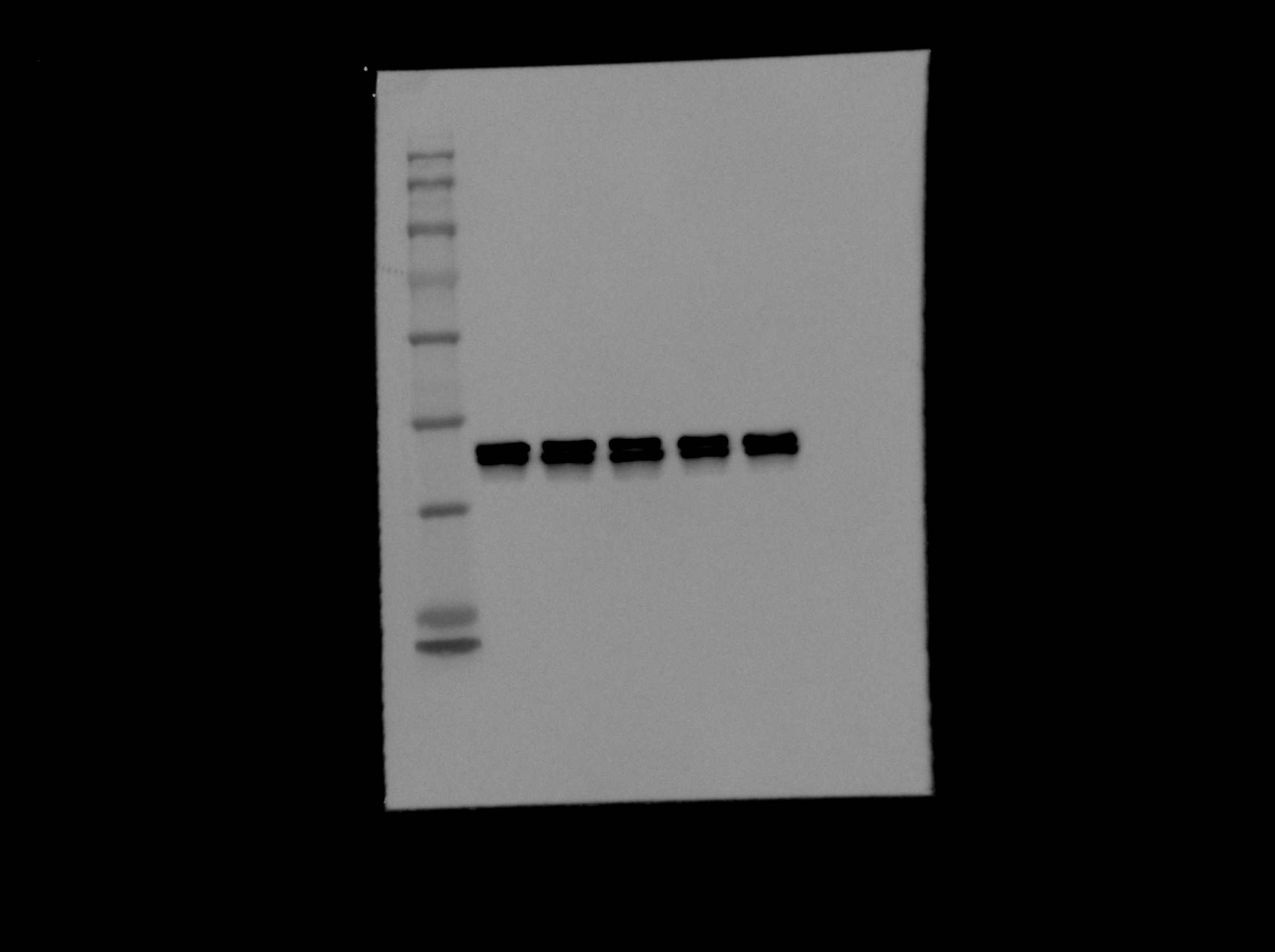
**

**
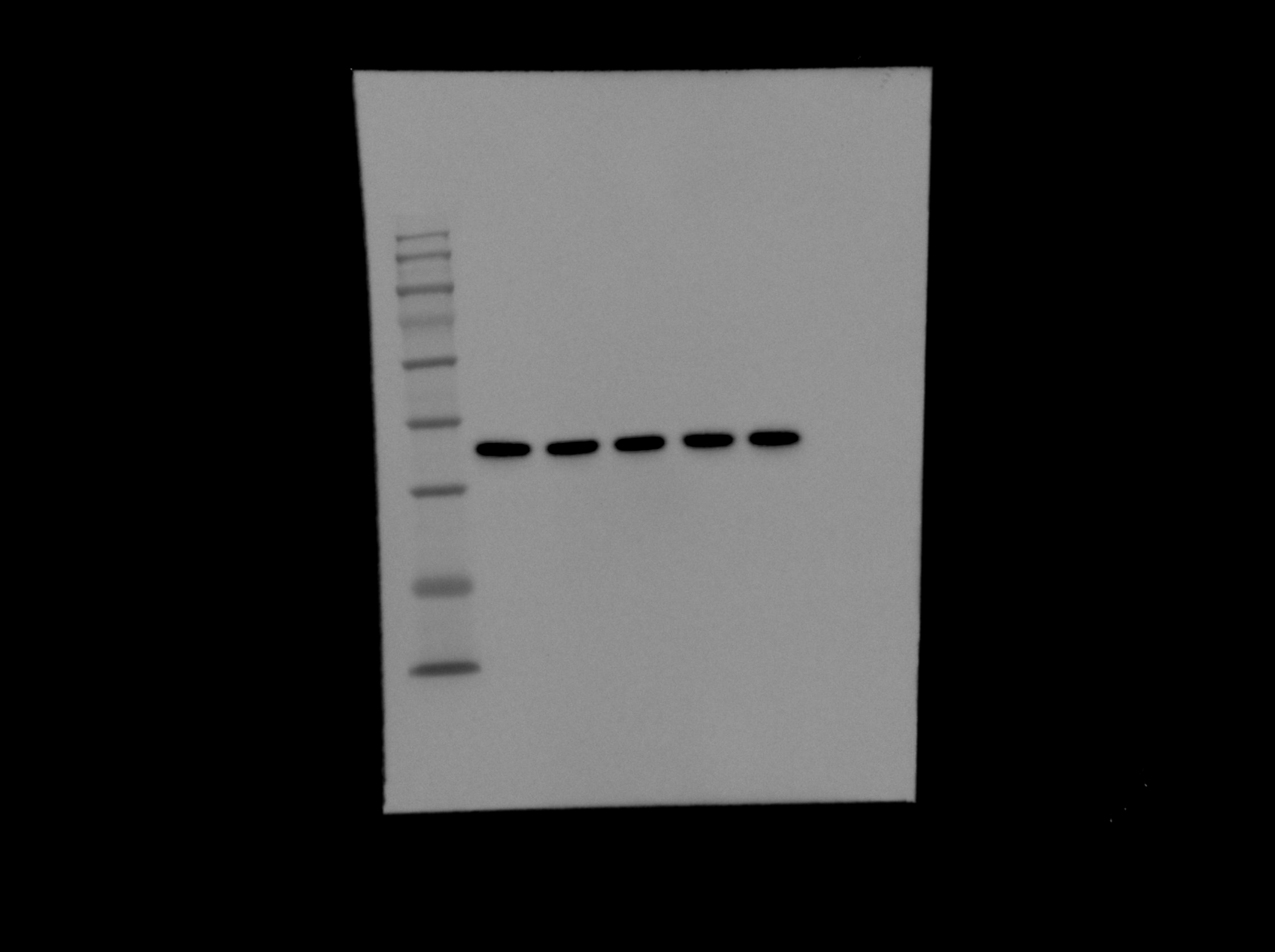
**
